# Supplementary material for: Health seeking behaviours and private sector delivery of care for non-communicable diseases in low- and middle-income countries: protocol for a systematic review
Source: BMJ Open. 2023 Aug 24;13(8):e066213. doi: 10.1136/bmjopen-2022-066213 (PMC10450129; doi:10.1136/bmjopen-2022-066213)
Supplement: Supplementary data [file bmjopen-2022-066213supp001.pdf]

Health seeking behaviours and private sector delivery of care for non-communicable diseases in low- and middle-income countries: protocol for a systematic review

Supplementary Material - Full Search Strategies

1. Embase

('non communicable disease'/mj/de OR neoplasm/mj/exp OR 'cancer patient'/mj/exp OR cardiology/mj/de OR 'cancer screening'/mj/de OR 'cancer surgery'/mj/de OR 'diabetes mellitus'/mj/exp OR 'cardiovascular disease'/mj/exp OR 'cerebrovascular disease'/mj/exp OR 'obstructive airway disease'/mj/exp OR 'chronic respiratory tract disease'/mj/de OR hypertension/mj/exp OR (((non-communicab* OR noncommunicab*) NEAR/3 disease*) OR cancer* OR ((heart OR cardiac OR cardiovascul* OR cerebrovascul* OR myocardial*) NEAR/3 (disease* OR patient* OR emergenc* OR attack* OR infarct*)) OR cardiolog* OR oncolog* OR diabet* OR (chronic NEAR/3 (respirator* OR lung* OR pulmonar*) NEAR/3 (failure OR disease*)) OR COPD OR asthma* OR bronchitis OR ((lung* OR pulmonar*) NEAR/3 (emphysem*)) OR ((cerebrovascul* OR cerebro-vascul*) NEAR/3 accident*) OR stroke OR hypertens* OR (surg* NEAR/3 emergenc*)):ti,kw) AND ('resource limited setting'/de OR 'low middle income country'/de OR 'low income country'/de OR 'middle income country'/de OR 'developing country'/de OR 'Africa'/exp OR 'Asia'/de OR 'South and Central America'/exp OR ((resource* NEAR/3 (limit* OR constrain* OR low)) OR ((low OR middle) NEAR/3 income* NEAR/3 countr*) OR LMIC* OR (developing NEAR/3 (countr* OR world* OR nation*)) OR third-world* OR 3rd-world* OR countries OR Afghan* OR albania* OR Algeria* OR American-somoa* OR Argentin* OR Armeni* OR asia OR AZERBAIJAN* OR Bangladesh* OR Belarus OR Belize OR Benin OR Bhutan* OR Bolivia* OR Bosnia* OR Botswan* OR Brazil* OR Bulgaria* OR Burkina-Faso OR Burundi OR Cabo-Verde* OR Central-African-Republic OR Chad OR China* OR Chinese* OR Colombia* OR Comoros OR Congo* OR Costa-Rica* OR Croatia* OR Cuba* OR Ecuador* OR Eritrea* OR Eswatini* OR Ethiopia* OR Fiji* OR Gabon* OR Gambia* OR Grenada* OR Guinea* OR Guatemal* OR Guyan* OR Haiti* OR Iran* OR Iraq* OR Jamaic* OR Jordan OR Kazakhst* OR DPR-Korea OR North-Korea OR Liberia* OR Latin-Americ* OR Madagasc* OR Malawi* OR Malaysia* OR Maldives* OR Mali OR malese OR Marshall-Island* OR Mexico* OR Mexican OR Micronesia* OR Mozambique* OR Nepal* OR Niger OR Palestin* OR Panama* OR Romania* OR Rwand* OR Samoa* OR Sao-Tome* OR Senegal* OR Sierra-Leon* OR Somali* OR South-Afric* OR Sudan* OR Syria* OR Tajiki* OR Tanzania* OR Togo* OR Tokolau* OR Tonga* OR Uganda* OR Yemen* OR Zimbabwe* OR Angol* OR Banglad* OR Bhutan* OR Bolivia* OR Cabo-Verd* OR Cambodia* OR Cameroon* OR Côte-d-Ivoir* OR Djibout* OR Dominica* OR Egypt* OR El-Salvador* OR (Georgia NOT (usa OR us OR united-states)) OR Ghan* OR Hondur* OR

India OR Indones* OR Kenya* OR Kiribati* OR Kosov* OR Kyrgyz* OR PDR-Lao OR Laotian* OR Lebanon* OR Lebanese* OR Lesoth* OR Libya* OR Mauritania* OR Mauritius* OR Micronesia* OR Moldova* OR Mongolia* OR Montenegr* OR Morocco* OR Myanmar* OR Namibia* OR Nauru* OR Nicaragua* OR Nigeria* OR Niue OR Macedoni* OR Pakistan* OR Papua* OR Paraguay* OR Peru OR Philippin* OR Russian-Feder* OR São-Tomé* OR Serbia* OR Solomon-Island* OR South-Afric* OR Sri-Lank* OR Saint-Lucia* OR Saint-Vincent* OR Sub-Sahara* OR Subsahara* OR Sudan* OR Surinam* OR Swaziland* OR Thailand* OR Timor-Leste OR Tunisia* OR Turkey OR Turkmenist* OR Tuvalu* OR Ukrain* OR Uzbeki* OR Vanuatu* OR Venezuel* OR Vietnam* OR West-Bank OR Gaza OR Zambia* OR africa):de,ab,ti,kw) AND ('health seeking behavior'/mj/de OR 'health care cost'/mj/exp OR 'health care quality'/mj/de OR 'affordability'/mj/de OR 'health care access'/mj/exp OR 'accessibility'/mj/de OR 'walking distance'/mj/exp OR 'health care delivery'/mj/de OR 'financial stress'/mj/exp OR 'reimbursement'/mj/de OR 'reputation'/de OR 'health care utilization'/mj/exp OR 'out of pocket cost'/de OR 'out of pocket expenditure'/de OR 'out of pocket payment'/de OR 'out of pocket spending'/de OR 'health inequalities'/de OR 'inequalities'/de OR 'cost effectiveness analysis'/mj/de OR 'financial deficit'/mj/exp OR 'patient empowerment'/mj/de OR ('empowerment'/mj/de AND 'patient'/mj/de) OR 'patient-reported outcome'/mj/de OR (((health) NEAR/3 (seeking* OR inequalit*)) OR ((health* OR hospital*) NEAR/3 (cost* OR expenditur* OR econom*)) OR ((health* OR care) NEAR/3 (qualit* OR evaluat* OR access* OR deliver* OR utilization* OR utilisation* OR "use")) OR affordab* OR access OR accessib* OR proximit* OR distance* OR ((financial* OR econom*) NEAR/3 (stress OR burden* OR distress* OR pressure* OR strain*)) OR ((opening* OR after OR out-of*) NEAR/3 (hour*)) OR in-network* OR reimbursement* OR reputation* OR out-of-pocket* OR ((indirect) NEAR/3 (cost* OR expendit*)) OR benefit-incidence* OR ((cost*) NEAR/3 (effectiv* OR efficien*)) OR debt* OR indebt* OR (empowerment* NEAR/3 (self OR patient*)) OR (patient-reported NEAR/3 (outcome* OR experience* OR satisfaction*))):ti,kw) AND ('health care facilities and services'/mj/exp OR 'health care personnel'/mj/exp OR 'private sector'/mj/exp OR 'hospitalization'/mj/exp OR 'pharmacy (shop)'/mj/exp OR 'clinical pharmacy'/mj/de OR (((health* OR care OR public* OR private* OR nonprofit* OR non-profit* OR faith*) NEAR/3 (facilit* OR provider* OR service* OR based OR sector)) OR hospital* OR clinic OR ((health*) NEAR/3 (center* OR centre* OR post)) OR ((family OR general) NEAR/3 (practitioner* OR doctor)) OR ((primary OR secondary OR tertiary OR nursing) NEAR/3 (care)) OR specialist OR specialists OR ((home) NEAR/3 (care OR nursing OR help OR service* OR treatment*)) OR homecare* OR cascade-of-care* OR pharmacy OR pharmacies):ti,kw) AND [2000-2030]/py NOT ('editorial'/de) NOT ([Conference Abstract]/lim OR [Conference Review]/lim) NOT ('case report'/de OR (case-report):ti)

2. Medline

(*Noncommunicable Diseases/ OR exp *Neoplasms/ OR exp *Cardiology/ OR exp *Early Detection of Cancer/ OR exp *Diabetes Mellitus/ OR exp *Cardiovascular Diseases/ OR exp *Cerebrovascular Disorders/ OR exp *"Pulmonary Disease, Chronic Obstructive"/ OR exp *Hypertension/ OR (((non-communicab* OR noncommunicab*) ADJ3 disease*) OR cancer* OR ((heart OR cardiac OR cardiovascul* OR cerebrovascul* OR myocardial*) ADJ3 (disease* OR patient* OR emergenc* OR attack* OR infarct*)) OR cardiolog* OR oncolog* OR diabet* OR (chronic ADJ3 (respirator* OR lung* OR pulmonar*) ADJ3 (failure OR disease*)) OR COPD OR asthma* OR bronchitis OR ((lung* OR pulmonar*) ADJ3 (emphysem*)) OR ((cerebrovascul* OR cerebro-vascul*) ADJ3 accident*) OR stroke OR hypertens* OR (surg* ADJ3 emergenc*)).ti,kf) AND (Developing Countries/ OR exp Africa/ OR Asia/ OR Latin America/ OR ((resource* ADJ3 (limit* OR constrain* OR low)) OR ((low OR middle) ADJ3 income* ADJ3 countr*) OR LMIC* OR (developing ADJ3 (countr* OR world* OR nation*)) OR third-world* OR 3rd-world* OR countries OR Afghan* OR alban* OR Algeria* OR American-somoa* OR Argentin* OR Armeni* OR asia OR AZERBAIJAN* OR Bangladesh* OR Belarus OR Belize OR Benin OR Bhutan* OR Bolivia* OR Bosnia* OR Botswan* OR Brazil* OR Bulgaria* OR Burkina-Faso OR Burundi OR Cabo-Verde* OR Central-African-Republic OR Chad OR China* OR Chinese* OR Colombia* OR Comoros OR Congo* OR Costa-Rica* OR Croatia* OR Cuba* OR Ecuador* OR Eritrea* OR Eswatini* OR Ethiopia* OR Fiji* OR Gabon* OR Gambia* OR Grenada* OR Guinea* OR Guatemal* OR Guyan* OR Haiti* OR Iran* OR Iraq* OR Jamaic* OR Jordan OR Kazakhst* OR DPR-Korea OR North-Korea OR Liberia* OR Latin-Americ* OR Madagasc* OR Malawi* OR Malaysia* OR Maldives* OR Mali OR malese OR Marshall-Island* OR Mexico* OR Mexican OR Micronesia* OR Mozambique* OR Nepal* OR Niger OR Palestin* OR Panama* OR Romania* OR Rwand* OR Samoa* OR Sao-Tome* OR Senegal* OR Sierra-Leon* OR Somali* OR South-Afric* OR Sudan* OR Syria* OR Tajiki* OR Tanzania* OR Togo* OR Tokolau* OR Tonga* OR Uganda* OR Yemen* OR Zimbabwe* OR Angol* OR Banglad* OR Bhutan* OR Bolivia* OR Cabo-Verd* OR Cambodia* OR Cameroon* OR Cote-d-Ivoir* OR Djibout* OR Dominica* OR Egypt* OR El-Salvador* OR (Georgia NOT (usa OR us OR united-states)) OR Ghan* OR Hondur* OR India OR Indones* OR Kenya* OR Kiribati* OR Kosov* OR Kyrgyz* OR PDR-Lao OR Laotian* OR Lebanon* OR Lebanese* OR Lesoth* OR Libya* OR Mauritania* OR Mauritius* OR Micronesia* OR Moldova* OR Mongolia* OR Montenegr* OR Morocco* OR Myanmar* OR Namibia* OR Nauru* OR Nicaragua* OR Nigeria* OR Niue OR Macedoni* OR Pakistan* OR Papua* OR Paraguay* OR Peru OR Philippin* OR Russian-Feder* OR Sao-Tome* OR Serbia* OR Solomon-Island* OR South-Afric* OR Sri-Lank* OR Saint-Lucia* OR Saint-Vincent* OR Sub-Sahara* OR Subsahara* OR Sudan* OR Surinam* OR Swaziland* OR Thailand* OR Timor-Leste OR Tunisia* OR Turkey OR Turkmenist* OR Tuvalu* OR Ukrain* OR Uzbeki* OR Vanuatu* OR Venezuel* OR Vietnam* OR West-Bank OR Gaza OR Zambia* OR africa).ab,ti,kf) AND (*"Patient Acceptance of Health Care"/ OR exp *Health Care Costs/OR *"Quality

of Health Care"/ OR *"Costs and Cost Analysis"/ OR exp *Health Services Accessibility/ OR *Delivery of Health Care/ OR *Financial Stress/ OR *"Insurance, Health, Reimbursement"/ OR *Health Expenditures/ OR *Healthcare Disparities/ OR *Cost-Benefit Analysis/ OR *Patient Participation/ OR (*Empowerment/ AND *Patients/) OR *Patient Reported Outcome Measures/ OR (((health) ADJ3 (seeking* OR inequalit*)) OR ((health* OR hospital*) ADJ3 (cost* OR expenditur* OR econom*)) OR ((health* OR care) ADJ3 (qualit* OR evaluat* OR access* OR deliver* OR utilization* OR utilisation* OR "use")) OR affordab* OR accessib* OR proximit* OR distance* OR ((financial* OR econom*) ADJ3 (stress OR burden* OR distress* OR pressure* OR strain*)) OR ((opening* OR after OR out-of*) ADJ3 (hour*)) OR in-network* OR reimbursement* OR reputation* OR out-of-pocket* OR ((indirect) ADJ3 (cost* OR expendit*)) OR benefit-incidence* OR ((cost*) ADJ3 (effectiv* OR efficien*)) OR debt* OR indebt* OR (empowerment* ADJ3 (self OR patient*)) OR (patient-reported ADJ3 (outcome* OR experience* OR satisfaction*))).ti,kf) AND (exp *"Health Care Facilities, Manpower, and Services"/ OR (((health* OR care OR public* OR private* OR nonprofit* OR non-profit* OR faith*) ADJ3 (facilit* OR provider* OR service* OR based OR sector)) OR hospital* OR clinic OR ((health*) ADJ3 (center* OR centre* OR post)) OR ((family OR general) ADJ3 (practitioner* OR doctor)) OR ((primary OR secondary OR tertiary OR nursing) ADJ3 (care)) OR specialist OR specialists OR ((home) ADJ3 (care OR nursing OR help OR service* OR treatment*)) OR homecare* OR cascade-of-care* OR pharmacy OR pharmacies).ti,kf) AND 2000:2030.(sa_year) NOT (Editorial/) NOT (news OR congres* OR abstract* OR book* OR chapter* OR dissertation abstract*).pt. NOT (Case Reports/ OR (case-report).ti.)

3. Web of Science

TI=(((((non-communicab* OR noncommunicab*) NEAR/2 disease*) OR cancer* OR ((heart OR cardiac OR cardiovascul* OR cerebrovascul* OR myocardial*) NEAR/2 (disease* OR patient* OR emergenc* OR attack* OR infarct*)) OR cardiolog* OR oncolog* OR diabet* OR (chronic NEAR/2 (respirator* OR lung* OR pulmonar*) NEAR/2 (failure OR disease*)) OR COPD OR asthma* OR bronchitis OR ((lung* OR pulmonar*) NEAR/2 (emphysem*)) OR ((cerebrovascul* OR cerebro-vascul*) NEAR/2 accident*) OR stroke OR hypertens* OR (surg* NEAR/2 emergenc*)))) AND TS=((((resource* NEAR/2 (limit* OR constrain* OR low)) OR ((low OR middle) NEAR/2 income* NEAR/2 countr*) OR LMIC* OR (developing NEAR/2 (countr* OR world* OR nation*)) OR third-world* OR 3rd-world* OR countries OR Afghan* OR albania* OR Algeria* OR American-somoa* OR Argentin* OR Armeni* OR asia OR AZERBAIJAN* OR Bangladesh* OR Belarus OR Belize OR Benin OR Bhutan* OR Bolivia* OR Bosnia* OR Botswan* OR Brazil* OR Bulgaria* OR Burkina-Faso OR Burundi OR Cabo-Verde* OR Central-African-Republic OR Chad OR China* OR Chinese* OR Colombia* OR Comoros OR Congo* OR Costa-Rica* OR Croatia* OR Cuba* OR Ecuador* OR Eritrea* OR Eswatini* OR Ethiopia* OR Fiji* OR Gabon* OR Gambia* OR

Grenada* OR Guinea* OR Guatemal* OR Guyan* OR Haiti* OR Iran* OR Iraq* OR Jamaic* OR Jordan OR Kazakhst* OR DPR-Korea OR North-Korea OR Liberia* OR Latin-Americ* OR Madagasc* OR Malawi* OR Malaysia* OR Maldives* OR Mali OR malese OR Marshall-Island* OR Mexico* OR Mexican OR Micronesia* OR Mozambique* OR Nepal* OR Niger OR Palestin* OR Panama* OR Romania* OR Rwand* OR Samoa* OR Sao-Tome* OR Senegal* OR Sierra-Leon* OR Somali* OR South-Afric* OR Sudan* OR Syria* OR Tajiki* OR Tanzania* OR Togo* OR Tokolau* OR Tonga* OR Uganda* OR Yemen* OR Zimbabwe* OR Angol* OR Banglad* OR Bhutan* OR Bolivia* OR Cabo-Verd* OR Cambodia* OR Cameroon* OR Cote-d-Ivoir* OR Djibout* OR Dominica* OR Egypt* OR El-Salvador* OR (Georgia NOT (usa OR us OR united-states)) OR Ghan* OR Hondur* OR India OR Indones* OR Kenya* OR Kiribati* OR Kosov* OR Kyrgyz* OR PDR-Lao OR Laotian* OR Lebanon* OR Lebanese* OR Lesoth* OR Libya* OR Mauritania* OR Mauritius* OR Micronesia* OR Moldova* OR Mongolia* OR Montenegr* OR Morocco* OR Myanmar* OR Namibia* OR Nauru* OR Nicaragua* OR Nigeria* OR Niue OR Macedoni* OR Pakistan* OR Papua* OR Paraguay* OR Peru OR Philippin* OR Russian-Feder* OR São-Tomé* OR Serbia* OR Solomon-Island* OR South-Afric* OR Sri-Lank* OR Saint-Lucia* OR Saint-Vincent* OR Sub-Sahara* OR Subsahara* OR Sudan* OR Surinam* OR Swaziland* OR Thailand* OR Timor-Leste OR Tunisia* OR Turkey OR Turkmenist* OR Tuvalu* OR Ukrain* OR Uzbeki* OR Vanuatu* OR Venezuel* OR Vietnam* OR West-Bank OR Gaza OR Zambia* OR africa))) AND TI=(((((health) NEAR/2 (seeking* OR inequalit*)) OR ((health* OR hospital*) NEAR/2 (cost* OR expenditur* OR econom*)) OR ((health* OR care) NEAR/2 (qualit* OR evaluat* OR access* OR deliver* OR utilization* OR utilisation* OR "use")) OR affordab* OR access OR accessib* OR proximit* OR distance* OR ((financial* OR econom*) NEAR/2 (stress OR burden* OR distress* OR pressure* OR strain*)) OR ((opening* OR after OR out-of*) NEAR/2 (hour*)) OR in-network* OR reimbursement* OR reputation* OR out-of-pocket* OR ((indirect) NEAR/2 (cost* OR expendit*)) OR benefit-incidence* OR ((cost*) NEAR/2 (effectiv* OR efficien*)) OR debt* OR indebt* OR (empowerment* NEAR/3 (self OR patient*)) OR (patient-reported NEAR/3 (outcome* OR experience* OR satisfaction*)))) AND ((((health* OR care OR public* OR private* OR nonprofit* OR non-profit* OR faith*) NEAR/2 (facilit* OR provider* OR service* OR based OR sector)) OR hospital* OR clinic OR ((health*) NEAR/2 (center* OR centre* OR post)) OR ((family OR general) NEAR/2 (practitioner* OR doctor)) OR ((primary OR secondary OR tertiary OR nursing) NEAR/2 (care)) OR specialist OR specialists OR ((home) NEAR/2 (care OR nursing OR help OR service* OR treatment*)) OR homecare* OR cascade-of-care* OR pharmacy OR pharmacies))) AND py=(2000-2030) AND DT=(Article OR Review OR Letter OR Early Access) NOT TI=(case-report)

4. Econlit

TI(non-communicab* OR noncommunicab* OR cancer* OR cardiac*OR cardiolog* OR oncolog* OR diabet* OR COPD OR asthma* OR bronchitis OR stroke OR hypertens*) **AND** ((((health* OR care OR

public* OR private* OR nonprofit* OR non-profit* OR faith*) N/2 (facilit* OR provider* OR service* OR based OR sector)) OR hospital* OR clinic OR ((health*) N/2 (center* OR centre* OR post)) OR ((family OR general) N/2 (practitioner* OR doctor)) OR ((primary OR secondary OR tertiary OR nursing) N/2 (care)) OR specialist OR specialists OR ((home) N/2 (care OR nursing OR help OR service* OR treatment*)) OR homecare* OR cascade-of-care* OR pharmacy OR pharmacies)) NOT PT (news OR comment* OR editorial* OR congres* OR abstract* OR book* OR chapter* OR dissertation abstract*) **AND** AB,TI((LMIC* OR third-world* OR 3rd-world* OR Afghan* OR albania* OR Algeria* OR American-somoa* OR Argentin* OR Armeni* OR asia OR AZERBAIJAN* OR Bangladesh* OR Belarus OR Belize OR Benin OR Bhutan* OR Bolivia* OR Bosnia* OR Botswan* OR Brazil* OR Bulgaria* OR Burkina-Faso OR Burundi OR Cabo-Verde* OR Central-African-Republic OR Chad OR China* OR Chinese* OR Colombia* OR Comoros OR Congo* OR Costa-Rica* OR Croatia* OR Cuba* OR Ecuador* OR Eritrea* OR Eswatini* OR Ethiopia* OR Fiji* OR Gabon* OR Gambia* OR Grenada* OR Guinea* OR Guatemal* OR Guyan* OR Haiti* OR Iran* OR Iraq* OR Jamaic* OR Jordan OR Kazakhst* OR DPR-Korea OR North-Korea OR Liberia* OR Latin-Americ* OR Madagasc* OR Malawi* OR Malaysia* OR Maldives* OR Mali OR malese OR Marshall-Island* OR Mexico* OR Mexican OR Micronesia* OR Mozambique* OR Nepal* OR Niger OR Palestin* OR Panama* OR Romania* OR Rwand* OR Samoa* OR Sao-Tome* OR Senegal* OR Sierra-Leon* OR Somali* OR South-Afric* OR Sudan* OR Syria* OR Tajiki* OR Tanzania* OR Togo* OR Tokolau* OR Tonga* OR Uganda* OR Yemen* OR Zimbabwe* OR Angol* OR Banglad* OR Bhutan* OR Bolivia* OR Cabo-Verd* OR Cambodia* OR Cameroon* OR Cote-d-Ivoir* OR Djibout* OR Dominica* OR Egypt* OR El-Salvador* OR (Georgia NOT (usa OR us OR united-states)) OR Ghan* OR Hondur* OR India OR Indones* OR Kenya* OR Kiribati* OR Kosov* OR Kyrgyz* OR PDR-Lao OR Laotian* OR Lebanon* OR Lebanese* OR Lesoth* OR Libya* OR Mauritania* OR Mauritius* OR Micronesia* OR Moldova* OR Mongolia* OR Montenegr* OR Morocco* OR Myanmar* OR Namibia* OR Nauru* OR Nicaragua* OR Nigeria* OR Niue OR Macedoni* OR Pakistan* OR Papua* OR Paraguay* OR Peru OR Philippin* OR Russian-Feder* OR São-Tomé* OR Serbia* OR Solomon-Island* OR South-Afric* OR Sri-Lank* OR Saint-Lucia* OR Saint-Vincent* OR Sub-Sahara* OR Subsahara* OR Sudan* OR Surinam* OR Swaziland* OR Thailand* OR Timor-Leste OR Tunisia* OR Turkey OR Turkmenist* OR Tuvalu* OR Ukrain* OR Uzbeki* OR Vanuatu* OR Venezuel* OR Vietnam* OR West-Bank OR Gaza OR Zambia* OR africa)) NOT PT (letter* OR news OR comment* OR editorial* OR congres* OR abstract* OR book* OR chapter* OR dissertation abstract*)

5. Global Index Medicus

TI:("non communicable" OR noncommunicable* OR cancer* OR cardiovascular* OR diabet* OR COPD OR asthma* OR bronchit* OR stroke OR hypertens* OR "heart disease" OR "cardiac disease" OR cerebrovascular* OR myocardial* OR "heart attack" OR "heart infarct" OR "cardiac attack" OR "cardiac

infarct" OR cardiolog* OR oncolog*) AND ti:("health seeking" OR "health inequality" OR "patient-acceptance" OR "health cost" OR "health evaluation" OR access OR affordabilit* OR "cost analysis" OR accessibility OR "financial stress" OR "patient reported" OR "walking distance" OR "out of pocket" OR "out-of-pocket" OR debt OR "patient empowerment" OR "self empowerment" OR "in network" OR "cost effectiveness" OR "cost efficiency" OR "patient reported outcome") AND TI:("health service" OR "health facility" OR "health provider" OR "health centre" OR "health center" OR "care service" OR "care facility" OR "care provider" OR "public service" OR "public facility" OR "public provider" OR "private service" OR "private facility" OR "private provider" OR hospital* OR clinic* OR specialist* OR homecare* OR non-profit* OR "family practitioner" OR "family doctor" OR "general practitioner")

6. Google Scholar

non-communicable|cancer|cardiovascular|diabetes|COPD|asthma|stroke|hypertension LMIC|'low middle income countries' 'health seeking|cost|quality|access|utilization' 'health provider|facility|center|centre|service'|hospital|clinic|pharmacy
