# Supplementary material for: Health seeking behaviours and private sector delivery of care for non-communicable diseases in low- and middle-income countries: protocol for a systematic review
Source: BMJ Open. 2023 Aug 24;13(8):e066213. doi: 10.1136/bmjopen-2022-066213 (PMC10450129; doi:10.1136/bmjopen-2022-066213)
Supplement: Author's manuscript [file bmjopen-2022-066213.draft_revisions.pdf]

BMJ Open

BMJ Open is committed to open peer review. As part of this commitment we make the peer review history of every article we publish publicly available.

When an article is published we post the peer reviewers' comments and the authors' responses online. We also post the versions of the paper that were used during peer review. These are the versions that the peer review comments apply to.

The versions of the paper that follow are the versions that were submitted during the peer review process. They are not the versions of record or the final published versions. They should not be cited or distributed as the published version of this manuscript.

BMJ Open is an open access journal and the full, final, typeset and author-corrected version of record of the manuscript is available on our site with no access controls, subscription charges or pay-per-view fees (http://bmjopen.bmj.com).

If you have any questions on BMJ Open's open peer review process please email info.bmjopen@bmj.com

BMJ Open

Health seeking behaviours and private sector delivery of care for non-communicable diseases in low- and middle-income countries: protocol for a systematic review

Journal:	*BMJ Open*
Manuscript ID	bmjopen-2022-066213
Article Type:	Protocol
Date Submitted by the Author:	30-Jun-2022
Complete List of Authors:	Brindley, Callum; Erasmus University Rotterdam<br>Wijemunige, Nilmini; Erasmus University Rotterdam; Institute for Health Policy<br>Dieteren, Charlotte; Erasmus University Rotterdam<br>Bom, Judith; Erasmus University Rotterdam<br>Engel, Maarten; Erasmus University Rotterdam<br>Meessen, Bruno; World Health Organization<br>Bonfrer, IEJ ; Erasmus Universiteit Rotterdam,
Keywords:	Health economics < HEALTH SERVICES ADMINISTRATION & MANAGEMENT, Health policy < HEALTH SERVICES ADMINISTRATION & MANAGEMENT, Organisation of health services < HEALTH SERVICES ADMINISTRATION & MANAGEMENT, Quality in health care < HEALTH SERVICES ADMINISTRATION & MANAGEMENT

SCHOLARONE™
Manuscripts

Health seeking behaviours and private sector delivery of care for non-communicable diseases in low- and middle-income countries: protocol for a systematic review

Authors list:

Callum Brindley[1,2]*, Nilmini Wijemunige[1,3], Charlotte Dieteren[1,2], Judith Bom[1,2], Maarten Engel[4], Bruno Meessen[5] and Igna Bonfrer[1,2]

Affiliations:

[1] Erasmus School of Health Policy and Management, Erasmus University Rotterdam, Rotterdam, The Netherlands

[2] Erasmus Centre for Health Economics, Erasmus University Rotterdam, Rotterdam, The Netherlands

[3] Institute for Health Policy, Colombo, Sri Lanka

[4] Medical Library, Erasmus Medical Centre, Erasmus University Rotterdam, Rotterdam, The Netherlands

[5] The World Health Organization, Geneva, Switzerland

*Correspondence author P.O. Box 1738, 3000 DR Rotterdam, The Netherlands. Email: brindley@eshpm.eur.nl

Tel: +31 10 408 8555

Word Count: 1915

For peer review only

Abstract

Introduction The burden of noncommunicable diseases (NCDs) has increased substantially in low- and middle-income countries (LMICs) and adapting health service delivery models to address this remains a challenge. Many NCD patients seek private care at different points in their encounters with the health system, but the determinants and consequences of these choices are insufficiently understood. The proposed systematic review will help inform the governance of mixed health systems towards achieving the goal of universal health coverage. This protocol details our intended methodological and analytical approaches, based on the Preferred Reporting Items for Systematic Reviews and Meta-Analyses (PRISMA)

Methods and analysis Following the PRISMA approach, this systematic review will develop a descriptive synthesis of the determinants and consequences of private health care utilisation for NCDs in LMICs. To identify relevant studies, Embase, Medline, Web of Science Core Collection, EconLit, Global Index Medicus and Google Scholar will be searched for the period 1 January 2010 and 30 June 2022 with additional searching of reference lists. The study selection process will involve a title-abstract and full text review, guided by clearly defined inclusion and exclusion criteria. A quality and risk of bias assessment will be done for each study using the Mixed Methods Appraisal Tool (MMAT).

Ethics and dissemination Ethical approval is not required because this review is based on data collected from publicly available materials. The results will be published in a peer-reviewed journal and presented at related scientific events.

PROSPERO registration number CRD42022340059

Keywords health seeking behaviour, private sector, noncommunicable diseases, cardiovascular disease, diabetes, respiratory diseases, cancer, low- and middle-income countries

Strengths and limitations of this study

- This is a systematic review protocol that proposes a timely contribution to the challenge of adapting health care service delivery to prevent, treat and manage NCDs in LMICs.
- We define the private sector broadly to capture its breadth and complexity.
- Although we will consider all LMICs, this systematic review is limited to studies published in English, so does not include relevant literature in other languages.
- We limit our attention to literature from the past decade which we contend is most relevant given recent changes in disease burden, risk factors and demographics in LMICs.
- We will not perform a meta-analysis because we expect highly diverse study characteristics, including design type, setting, intervention and outcome.

For peer review only - http://bmjopen.bmj.com/site/about/guidelines.xhtml

For peer review only

For peer review only - http://bmjopen.bmj.com/site/about/guidelines.xhtml

1. Introduction

The rising burden of non-communicable diseases (NCDs) in low- and middle-income countries (LMICs) [1,2] calls for policy responses that engage the whole health system, including private providers. While the appropriate role of the private sector in the delivery of health care has been heavily debated [3–6], it is well known that patients seek private care at different points in their encounters with the health system.[7–10] Research into the reasons for visiting private providers and the comparative quality of this care in LMICs has largely focused on infectious diseases, and maternal and child health.[11]

With regard to NCDs, the determinants and consequences of private health care utilisation in LMICs remains insufficiently understood. Importantly, NCDs have an etiology linked to behavioural risk factors and require an organisation of services and delivery of care that is different from that of acute illnesses. Specifically, a focus on prevention and primary care that empowers patients and prioritises information sharing and coordination across levels of care and providers.[12,13] This is imperative for the achievement of Sustainable Development Goal (SDG) target 3.4 – a one-third reduction in premature mortality from NCDs by 2030.

Adapting health service delivery models to respond to NCDs remains a challenge.[14] In many LMICs, national NCD strategies tend to focus on the public sector to the exclusion of the private sector despite the two being entwined elements of the whole health system. Many LMICs with universal health coverage (UHC) benefits packages also only offer limited coverage for NCDs, such that patients must pay out-of-pocket even in public health facilities. The risk is that this discourages and distorts health seeking behaviours, leading to ineffective care, poor health outcomes and catastrophic health spending.[15,16] Understanding better the reality of health seeking behaviours for NCDs, including the possible role already taken by private providers, will be key for the design and implementation of appropriate strategies and interventions, across the different building blocks of the health system.[17]

This systematic review has been initiated by the World Health Organization's (WHO) Department of Noncommunicable Disease, and Department of Health System Financing and Governance. It follows work by a WHO Advisory Group on Governance of the Private Sector and a systematic review of private sector delivery of quality care for maternal, newborn and child health in LMICs.[18,19] This review will contribute to an understanding of health seeking behaviour for NCDs in the private sector in LMICs, which will in turn help inform the governance of mixed health systems in the pursuit of UHC and the achievement of the SDGs.

For peer review only - http://bmjopen.bmj.com/site/about/guidelines.xhtml

2. Methods and analysis

2.1. Aim and research questions

Our systematic review aims to document the determinants and consequences of private health care utilisation for NCDs in LMICs. To this end, we will answer the following questions.

1. *What are the health care seeking behaviours of individuals with regard to NCDs in LMICs?*
    a. *From whom is health care obtained?*
    b. *What are the determinants that influence the choice of health care?*
    c. *What are the individual and collective consequences of these health care choices?*

2. *What are the major issues, knowledge gaps, opportunities and challenges that arise from private providers supplying health care services for NCDs in LMICs?*

2.2. Study design and protocol

This study is guided by the Preferred Reporting Items for Systematic Reviews and Meta-Analyses (PRISMA) and its extension for literature searches PRISMA-S.[20,21] PRISMA is a systematic approach to map and synthesise existing evidence, identify knowledge gaps and inform future research. This review was registered with PROSPERO (CRD42022340059) on 15 June 2022.

2.3. Inclusion/exclusion criteria

Table 1 outlines the Populations, Interventions, Control, Outcomes, Timeframe, Setting (PICOTS) criteria to be used. We focus on adults and define the scope of health care to encompass the prevention, diagnosis, treatment and/or management of disease. We restrict our search to four groups of NCDs responsible for over 80% of all premature NCD deaths and identified by SDG target 3.4. Specifically, cardiovascular diseases, cancers, respiratory diseases and diabetes. With regard to cancers, we limit our scope to the top five cancers with the greatest disease burden for each sex.[22] We use the World Health Organization's operational definition of the private health sector as the individuals and organizations that are neither owned nor directly controlled by governments, and are involved in the provision of health services (i.e. formal and informal providers as well as for-profit and non-profit entities.[23] Our outcomes of interest are the determinants and consequences of health

For peer review only - http://bmjopen.bmj.com/site/about/guidelines.xhtml

For peer review only

seeking behaviour at both the individual and collective level, which are described in more detailed under the search elements and key terms. We focus on the timeframe from January 2010 to June 2022 because this period coincides with the increase in the NCD disease burden in LMICs. This also avoids duplication of a broader systematic review into the private provision of health services in LMICs conducted in 2011.[24] Lastly, the study looks at settings in LMICs as defined by the World Bank classification for 2022 (i.e. countries with a gross national income per capita of $4,095 or less).[25]

Table 1 - PICOTS criteria used in the systematic review

Population	Adults and households at risk, or diagnosed with a non-communicable disease
Interventions	Private sector provision of health care services that involves the prevention, diagnosis, treatment and/or management of non-communicable disease
Control	Not applicable
Outcomes	Determinants and consequences of health care seeking behaviour, at the individual and population level
Timeframe	1 January 2010 to 30 June 2022
Setting	Low-income and middle-income countries

For inclusion, studies must adhere to all elements defined in Table 1. They must be in English and published in a peer-reviewed journal. We will include both qualitative and quantitative studies, including randomized and non-randomized study designs (i.e., cross-sectional, case-control and cohort observational studies). Editorials, commentaries, reviews, and protocols will be excluded.

Studies must address private sector health care and may do so in a comparison with the public sector. However, we will exclude studies that only report aggregated data (i.e., private and public health sector outcome data combined). Lastly, we will also exclude vaccination programmes associated with NCDs (e.g., human papillomavirus) because they are government-led, as well as settings with irregular contextual circumstances (e.g., conflict, economic and political crises) since they are less likely to offer generalisable insights.

2.4. Search strategy

Our search strategy was developed with the assistance of an information specialist (ME). The following electronic databases will be searched: Embase, Medline, Web of Science, Econlit, Global Index

For peer review only - http://bmjopen.bmj.com/site/about/guidelines.xhtml

Medicus. We will also search Google Scholar and download the 200 most relevant references in line with common practice.[26] Table 2 provides the search elements and key terms we will use. Specifically, it contains terms for 1) non communicable diseases, 2) health care providers in LMICs, and 3) the determinants and consequences of health seeking behaviour and related terms. The search was developed in Embase, optimized for sensitivity, then translated to other databases following the method described by Bramer et al 2018.[27] The search strategies for Embase and Medline use relevant thesaurus terms from Emtree and Medical Subject Headings (MeSH) respectively. In all databases, the terms will be searched in titles, author keywords and major index terms; the search element for setting (health care providers in LMICs) will also be searched in abstracts. The full search strategies of all databases are provided in the Supplementary Material. References will be imported into EndNote and duplicates removed.[28] Reference lists of revelvant reviews and all included articles will also be screened for potential additional sources missed by the search. We do not plan to contact authors or subject expects.

Table 2 - Search elements and key terms

Search element	Key terms
What: noncommunicable diseases	noncommunicable diseases; cardiovascular disease; heart attack; stroke; myocardial infarction; hypertension; diabetes; cancer (top 5 for each sex: lung, breast, colorectum, prostate, stomach, liver, cervix, thyroid); chronic respiratory disease; chronic obstructive pulmonary disease; asthma)
Where: health care providers in low- and middle-income countries	hospital, health clinic, community health centre, health centre, health post, family practitioner, general practitioner, primary care, secondary care, tertiary care, specialist, home care, nursing home, pharmacy, public provider, private provider, nonprofit provider, faith-based provider, low-income countries, middle-income countries, lower-middle income countries, upper-middle income countries, list of all LMIC countries

For peer review only - http://bmjopen.bmj.com/site/about/guidelines.xhtml

Why and what happens: determinants and consequences of health care decisions / health seeking behaviour	quality; cost; affordability; accessibility; proximity; distance; opening hours; in-network provider; reimbursement; reputation; health service delivery; health outcome; utilization; out-of-pocket payment; indirect costs; catastrophic health care expenditure; debt; health inequalities; health information; supply; fee for service; empowerment; patient reported experience

2.5. Screening process

The articles resulting from the search will be screened by a team of two pairs (CB-JB & NW-CD) working in the Rayyan Reference Manager. After removing duplicates, each article will be title-abstract screened independently by two individuals using the eligibility criteria described above. To reduce bias, author names will not be visible during this screening stage. Before commencing screening by pairs, we will also conduct a pilot phase in which a random selection of 100 articles will be reviewed and discussed jointly to harmonise our approach. Articles adhering to all criteria will be selected for full-text review. Title-abstracts will be screened in three lots. Any conflicts will be reviewed within the pair at the conclusion of reviewing each lot, to allow for further harmonization within the pair. If an inclusion/exclusion decision cannot be made based on the title and abstract, the article will be advanced to full-text screening. In the case of a conflicting inclusion/exclusion decision, the other pair in the team will also review the article and the supervising researcher (IB) will provide a final adjudication if needed. Articles selected for full-text screening will be reviewed by two individuals and a decision to exclude at this stage will be documented. Confirmed texts for inclusion in the systematic review will be advanced to the next stage of data extraction.

2.6. Data extraction

We will extract data using a framework, which we pilot and refine, organised into the following categories:

1. Study details (e.g., authors, title, year, journal)
2. Study characteristics (e.g., qualitative/quantitative, study design, research objectives, country(s), disease(s), sample population, provider, health care)
3. Determinants of health care decisions

For peer review only - http://bmjopen.bmj.com/site/about/guidelines.xhtml

   a. contextual characteristics (e.g., distance, affordability, quality)
   b. individual characteristics (e.g., demographic, education, wealth)
   c. health outcomes (e.g., effectiveness, satisfaction, empowerment)
4. Consequences of health care decisions
   a. financial protection (e.g., out-of-pocket payments, impoverishment)
   b. utilisation and quality (e.g., utilisation, morbidity, mortality)
   c. equity (e.g., inequalities, discrimination)

Each full-text article included in the systematic review will be read by two individuals with one person taking the lead to extract the data, and the second person reviewing this work.

2.7. Quality assessment

We will use the Mixed Methods Appraisal Tool (MMAT) to assess the methodological quality and risk of bias of the reviewed full-text articles.[29] MMAT is a critical appraisal tool intended for systematic reviews that include diverse study designs (i.e., quantitative, qualitative and mixed methods). Each full-text article will be assessed by a team of two pairs (CB-JB & NW-CD) with one person taking the lead and the second person reviewing for completeness and accuracy. Discrepancies and disagreements will be resolved through discussion.

2.8. Synthesis

The team will meet to discuss and analyse the data extracted, which we will consolidate using a descriptive synthesis including a summary of the evidence, gaps, and limitations. As described above in the data extraction, we will synthesise the results into three categories of determinants (contextual characteristics, individual characteristics, and health outcomes) and three categories of consequences (financial protection, utilisation and quality, and equity). We will also develop a synthesis of the results that concern the interaction of health seeking determinants and consequences. A flow diagram of the inclusion/exclusion pathways and the descriptive statistics of the included studies and their outcomes will be developed to complement the analysis.

2.9. Stakeholder consultation

For peer review only

The initial findings of this systematic review will be disseminated at a meeting in October 2022 on the health economics of NCDs in LMICs, which is to be jointly convened by the World Health Organization, the University of Lausanne and the Erasmus School of Health Policy and Management. Participants will include policy makers, academics, and health care practitioners from LMICs who will have the opportunity to discuss and validate the interpretation of the results.

2.10. Patient and public involvement

None

3. Ethics and dissemination

Ethical approval is not required because this is a review and collection of data based on publicly available materials. The results will be published in a topic relevant journal and presented at related scientific events. Understanding the reality of health seeking behaviours for NCDs will be key for the design and implementation of interventions to strengthen the building blocks of the health system: from regulation of the health workforce and provider payment reform to benefit package design and the digitalisation of patient files. This systematic review will be a timely contribution to the challenge of adapting health care service delivery to prevent, treat and manage NCDs in LMICs.

For peer review only

Acknowledgements We wish to acknowledge the valuable contributions of Annalise Belloni, Owen O'Donnell, Tom van Ourti and Eddy van Doorslaer who provided input for this systematic review protocol.

Contributions IB, BM, ME and CB conceptualised the protocol. CB wrote the first draft with input from NW, CD, JB, ME and IB. All authors contributed to subsequent revisions and approved the protocol prior to its submission. All authors are accountable for the research presented.

Funding This work was supported jointly by the Non-Communicable Disease Department, and Health System Financing and Governance Department of the World Health Organization. The content of this study is the sole responsibility of the authors and does not represent the official views of World Health Organization.

Competing interests None declared

Patient consent for publication Not applicable

Provenance and peer review Not commissioned, externally peer reviewed.

Data availability statement Data sharing not applicable as no datasets generated and/or analysed for this study

Supplementary material This content has been supplied by the author(s). It has not been vetted by BMJ Publishing Group Limited (BMJ) and may not have been peer-reviewed. Any opinions or recommendations discussed are solely those of the author(s) and are not endorsed by BMJ. BMJ disclaims all liability and responsibility arising from any reliance placed on the content. Where the content includes any translated material, BMJ does not warrant the accuracy and reliability of the translations (including but not limited to local regulations, clinical guidelines, terminology, drug names and drug dosages), and is not responsible for any error and/or omissions arising from translation and adaptation or otherwise.

Open access This is an open access article distributed in accordance with the Creative Commons Attribution Non Commercial (CC BY-NC 4.0) license, which permits others to distribute, remix, adapt, build upon this work non-commercially, and license their derivative works on different terms, provided the original work is properly cited, appropriate credit is given, any changes made indicated, and the use is non-commercial. See: http://creativecommons.org/licenses/by-nc/4.0/.

ORCID iDs

Callum Brindley https://orcid.org/0000-0002-5478-3974

Nilmini Wijemunige https://orcid.org/0000-0002-2241-3194

Charlotte Dieteren https://orcid.org/0000-0002-7809-1657

Judith Bom https://orcid.org/0000-0003-4477-7594

Maarten Engel https://orcid.org/0000-0002-1774-7042

Bruno Meessen https://orcid.org/0000-0002-0359-8621

For peer review only - http://bmjopen.bmj.com/site/about/guidelines.xhtml

Igna Bonfrer https://orcid.org/0000-0002-8570-0393

For peer review only

For peer review only - http://bmjopen.bmj.com/site/about/guidelines.xhtml

References

1 Bennett JE, Stevens GA, Mathers CD, *et al.* NCD Countdown 2030: worldwide trends in non-communicable disease mortality and progress towards Sustainable Development Goal target 3.4. *The Lancet* 2018;**392**:1072–88. doi:10.1016/S0140-6736(18)31992-5

2 Bukhman G. The Lancet NCDI Poverty Commission: bridging a gap in universal health coverage for the poorest billion. *The Lancet* 2020;**396**:991–1044. doi:10.1016/S0140-6736(20)31907-3

3 World Health Organization. *The World Health Report 2000. Health systems: improving performance*. Geneva, Switzerland: 2000.

4 World Bank. *World Development Report 2004: Making Services Work for Poor People*. Washington, DC, United States: : The World Bank 2003. doi:10.1596/0-8213-5468-X

5 Marriott A. Blind Optimism: Challenging the myths about private health care in poor countries. Oxfam International 2009. https://policy-practice.oxfam.org/resources/blind-optimism-challenging-the-myths-about-private-health-care-in-poor-countrie-114093/

6 Horton R, Clark S. The perils and possibilities of the private health sector. *The Lancet* 2016;**388**:540–1. doi:10.1016/S0140-6736(16)30774-7

7 Patouillard E, Goodman CA, Hanson KG, *et al.* Can working with the private for-profit sector improve utilization of quality health services by the poor? A systematic review of the literature. *Int J Equity Health* 2007;**6**:17. doi:10.1186/1475-9276-6-17

8 Berendes S, Heywood P, Oliver S, *et al.* Quality of private and public ambulatory health care in low and middle income countries: Systematic review of comparative studies. *PLoS Med* 2011;**8**:12. doi:10.1371/journal.pmed.1000433

9 Basu S, Andrews J, Kishore S, *et al.* Comparative Performance of Private and Public Healthcare Systems in Low- and Middle-Income Countries: A Systematic Review. *PLOS Med* 2012;**9**:e1001244. doi:10.1371/journal.pmed.1001244

10 Devi R, Kanitkar K, Narendhar R, *et al.* A Narrative Review of the Patient Journey Through the Lens of Non-communicable Diseases in Low- and Middle-Income Countries. *Adv Ther* 2020;**37**:4808–30. doi:10.1007/s12325-020-01519-3

For peer review only - http://bmjopen.bmj.com/site/about/guidelines.xhtml

11 Grépin KA. Private Sector An Important But Not Dominant Provider Of Key Health Services In Low- And Middle-Income Countries. *Health Aff (Millwood)* 2016;**35**:1214–21. doi:10.1377/hlthaff.2015.0862

12 World Health Organization. Global action plan for the prevention and control of noncommunicable diseases 2013-2020. Geneva, Switzerland: : World Health Organization 2013.

13 World Health Organization. *WHO package of essential noncommunicable (PEN) disease interventions for primary health care*. Geneva, Switzerland: : World Health Organization 2020. https://apps.who.int/iris/handle/10665/334186 (accessed 24 May 2022).

14 Kabir A, Karim MN, Islam RM, *et al.* Health system readiness for non-communicable diseases at the primary care level: a systematic review. *BMJ Open* 2022;**12**:e060387. doi:10.1136/bmjopen-2021-060387

15 Kankeu HT, Saksena P, Xu K, *et al.* The financial burden from non-communicable diseases in low- and middle-income countries: a literature review. *Health Res Policy Syst* 2013;**11**:31. doi:10.1186/1478-4505-11-31

16 Jan S, Laba T-L, Essue BM, *et al.* Action to address the household economic burden of non-communicable diseases. *The Lancet* 2018;**391**:2047–58. doi:10.1016/S0140-6736(18)30323-4

17 World Health Organization. Everybody's business - strengthening health systems to improve health outcomes : WHO's framework for action. World Health Organization 2007. https://apps.who.int/iris/handle/10665/43918 (accessed 15 Jun 2022).

18 World Health Organization. *Engaging the private health service delivery sector through governance in mixed health systems: strategy report of the WHO Advisory Group on the Governance of the Private Sector for Universal Health Coverage*. Geneva: : World Health Organization 2020. https://apps.who.int/iris/handle/10665/341057 (accessed 16 May 2022).

19 Lattof SR, Maliqi B. Private sector delivery of quality care for maternal, newborn and child health in low-income and middle-income countries: a mixed-methods systematic review protocol. *BMJ Open* 2020;**10**:e033141. doi:10.1136/bmjopen-2019-033141

20 Page MJ, McKenzie JE, Bossuyt PM, *et al.* The PRISMA 2020 statement: an updated guideline for reporting systematic reviews. *BMJ* 2021;:n71. doi:10.1136/bmj.n71

For peer review only - http://bmjopen.bmj.com/site/about/guidelines.xhtml

21 Rethlefsen ML, Kirtley S, Waffenschmidt S, *et al.* PRISMA-S: an extension to the PRISMA Statement for Reporting Literature Searches in Systematic Reviews. *Syst Rev* 2021;**10**:39. doi:10.1186/s13643-020-01542-z

22 Lim SS, Abbafati C, Abbas KM, *et al.* Global burden of 369 diseases and injuries in 204 countries and territories, 1990–2019: a systematic analysis for the Global Burden of Disease Study 2019. *The Lancet* 2020;**396**:1204–22. doi:10.1016/S0140-6736(20)30925-9

23 Klinton J. The Private Health Sector: An Operational Definition. Geneva, Switzerland: : World Health Organization 2020. https://www.who.int/docs/default-source/health-system-governance/private-health-sector-an-operational-definition.pdf?sfvrsn=5864e1f0_2&download=true (accessed 16 May 2022).

24 Montagu Dominic, Andrew Anglemyer, Mudita Tiwari, *et al.* Private versus public strategies for health service provision for improving health outcomes in resource-limited settings. San Francisco, United States: : University of California 2011. https://eppi.ioe.ac.uk/cms/Default.aspx?tabid=3307

25 World Bank. World Bank Country and Lending Groups. 2022.https://datahelpdesk-worldbank-org.eur.idm.oclc.org/knowledgebase/articles/906519-world-bank-country-and-lending-groups

26 Harzing A-W. *Publish or Perish*. https://harzing.com/resources/publish-or-perish (accessed 23 Jun 2022).

27 Bramer WM, de Jonge GB, Rethlefsen ML, *et al.* A systematic approach to searching: an efficient and complete method to develop literature searches. *J Med Libr Assoc JMLA* 2018;**106**:531–41. doi:10.5195/jmla.2018.283

28 Bramer WM, Giustini D, de Jonge GB, *et al.* De-duplication of database search results for systematic reviews in EndNote. *J Med Libr Assoc JMLA* 2016;**104**:240–3. doi:10.3163/1536-5050.104.3.014

29 Hong Q, Pluye P, Fàbregues S, *et al.* Mixed methods appraisal tool (MMAT), Version 2018. http://mixedmethodsappraisaltoolpublic.pbworks.com/w/file/fetch/127916259/MMAT_2018_criteria-manual_2018-08-01_ENG.pdf (accessed 9 Jun 2022).

For peer review only

Health seeking behaviours and private sector delivery of care for non-communicable diseases in low- and middle-income countries: protocol for a systematic review

Supplementary Material - Full Search Strategies

1. Embase

('non communicable disease'/mj/de OR neoplasm/mj/exp OR 'cancer patient'/mj/exp OR cardiology/mj/de OR 'cancer screening'/mj/de OR 'cancer surgery'/mj/de OR 'diabetes mellitus'/mj/exp OR 'cardiovascular disease'/mj/exp OR 'cerebrovascular disease'/mj/exp OR 'obstructive airway disease'/mj/exp OR 'chronic respiratory tract disease'/mj/de OR hypertension/mj/exp OR (((non-communicab* OR noncommunicab*) NEAR/3 disease*) OR cancer* OR ((heart OR cardiac OR cardiovascul* OR cerebrovascul* OR myocardial*) NEAR/3 (disease* OR patient* OR emergenc* OR attack* OR infarct*)) OR cardiolog* OR oncolog* OR diabet* OR (chronic NEAR/3 (respirator* OR lung* OR pulmonar*) NEAR/3 (failure OR disease*)) OR COPD OR asthma* OR bronchitis OR ((lung* OR pulmonar*) NEAR/3 (emphysem*)) OR ((cerebrovascul* OR cerebro-vascul*) NEAR/3 accident*) OR stroke OR hypertens* OR (surg* NEAR/3 emergenc*)):ti,kw) AND ('resource limited setting'/de OR 'low middle income country'/de OR 'low income country'/de OR 'middle income country'/de OR 'developing country'/de OR 'Africa'/exp OR 'Asia'/de OR 'South and Central America'/exp OR ((resource* NEAR/3 (limit* OR constrain* OR low)) OR ((low OR middle) NEAR/3 income* NEAR/3 countr*) OR LMIC* OR (developing NEAR/3 (countr* OR world* OR nation*)) OR third-world* OR 3rd-world* OR countries OR Afghan* OR albania* OR Algeria* OR American-somoa* OR Argentin* OR Armeni* OR asia OR AZERBAIJAN* OR Bangladesh* OR Belarus OR Belize OR Benin OR Bhutan* OR Bolivia* OR Bosnia* OR Botswan* OR Brazil* OR Bulgaria* OR Burkina-Faso OR Burundi OR Cabo-Verde* OR Central-African-Republic OR Chad OR China* OR Chinese* OR Colombia* OR Comoros OR Congo* OR Costa-Rica* OR Croatia* OR Cuba* OR Ecuador* OR Eritrea* OR Eswatini* OR Ethiopia* OR Fiji* OR Gabon* OR Gambia* OR Grenada* OR Guinea* OR Guatemal* OR Guyan* OR Haiti* OR Iran* OR Iraq* OR Jamaic* OR Jordan OR Kazakhst* OR DPR-Korea OR North-Korea OR Liberia* OR Latin-Americ* OR Madagasc* OR Malawi* OR Malaysia* OR Maldives* OR Mali OR malese OR Marshall-Island* OR Mexico* OR Mexican OR Micronesia* OR Mozambique* OR Nepal* OR Niger OR Palestin* OR Panama* OR Romania* OR Rwand* OR Samoa* OR Sao-Tome* OR Senegal* OR Sierra-Leon* OR Somali* OR South-Afric* OR Sudan* OR Syria* OR Tajiki* OR Tanzania* OR Togo* OR Tokolau* OR Tonga* OR Uganda* OR Yemen* OR Zimbabwe* OR Angol* OR Banglad* OR Bhutan* OR Bolivia* OR Cabo-Verd* OR Cambodia* OR Cameroon* OR Côte-d-Ivoir* OR Djibout* OR Dominica* OR Egypt* OR El-Salvador* OR (Georgia NOT (usa OR us OR united-states)) OR Ghan* OR Hondur* OR

For peer review only - http://bmjopen.bmj.com/site/about/guidelines.xhtml

India OR Indones* OR Kenya* OR Kiribati* OR Kosov* OR Kyrgyz* OR PDR-Lao OR Laotian* OR Lebanon* OR Lebanese* OR Lesoth* OR Libya* OR Mauritania* OR Mauritius* OR Micronesia* OR Moldova* OR Mongolia* OR Montenegr* OR Morocco* OR Myanmar* OR Namibia* OR Nauru* OR Nicaragua* OR Nigeria* OR Niue OR Macedoni* OR Pakistan* OR Papua* OR Paraguay* OR Peru OR Philippin* OR Russian-Feder* OR São-Tomé* OR Serbia* OR Solomon-Island* OR South-Afric* OR Sri-Lank* OR Saint-Lucia* OR Saint-Vincent* OR Sub-Sahara* OR Subsahara* OR Sudan* OR Surinam* OR Swaziland* OR Thailand* OR Timor-Leste OR Tunisia* OR Turkey OR Turkmenist* OR Tuvalu* OR Ukrain* OR Uzbeki* OR Vanuatu* OR Venezuel* OR Vietnam* OR West-Bank OR Gaza OR Zambia* OR africa):de,ab,ti,kw) AND ('health seeking behavior'/mj/de OR 'health care cost'/mj/exp OR 'health care quality'/mj/de OR 'affordability'/mj/de OR 'health care access'/mj/exp OR 'accessibility'/mj/de OR 'walking distance'/mj/exp OR 'health care delivery'/mj/de OR 'financial stress'/mj/exp OR 'reimbursement'/mj/de OR 'reputation'/de OR 'health care utilization'/mj/exp OR 'out of pocket cost'/de OR 'out of pocket expenditure'/de OR 'out of pocket payment'/de OR 'out of pocket spending'/de OR 'health inequalities'/de OR 'inequalities'/de OR 'cost effectiveness analysis'/mj/de OR 'financial deficit'/mj/exp OR 'patient empowerment'/mj/de OR ('empowerment'/mj/de AND 'patient'/mj/de) OR 'patient-reported outcome'/mj/de OR (((health) NEAR/3 (seeking* OR inequalit*)) OR ((health* OR hospital*) NEAR/3 (cost* OR expenditur* OR econom*)) OR ((health* OR care) NEAR/3 (qualit* OR evaluat* OR access* OR deliver* OR utilization* OR utilisation* OR "use")) OR affordab* OR access OR accessib* OR proximit* OR distance* OR ((financial* OR econom*) NEAR/3 (stress OR burden* OR distress* OR pressure* OR strain*)) OR ((opening* OR after OR out-of*) NEAR/3 (hour*)) OR in-network* OR reimbursement* OR reputation* OR out-of-pocket* OR ((indirect) NEAR/3 (cost* OR expendit*)) OR benefit-incidence* OR ((cost*) NEAR/3 (effectiv* OR efficien*)) OR debt* OR indebt* OR (empowerment* NEAR/3 (self OR patient*)) OR (patient-reported NEAR/3 (outcome* OR experience* OR satisfaction*))):ti,kw) AND ('health care facilities and services'/mj/exp OR 'health care personnel'/mj/exp OR 'private sector'/mj/exp OR 'hospitalization'/mj/exp OR 'pharmacy (shop)'/mj/exp OR 'clinical pharmacy'/mj/de OR (((health* OR care OR public* OR private* OR nonprofit* OR non-profit* OR faith*) NEAR/3 (facilit* OR provider* OR service* OR based OR sector)) OR hospital* OR clinic OR ((health*) NEAR/3 (center* OR centre* OR post)) OR ((family OR general) NEAR/3 (practitioner* OR doctor)) OR ((primary OR secondary OR tertiary OR nursing) NEAR/3 (care)) OR specialist OR specialists OR ((home) NEAR/3 (care OR nursing OR help OR service* OR treatment*)) OR homecare* OR cascade-of-care* OR pharmacy OR pharmacies):ti,kw) AND [2000-2030]/py NOT ('editorial'/de) NOT ([Conference Abstract]/lim OR [Conference Review]/lim) NOT ('case report'/de OR (case-report):ti)

For peer review only - http://bmjopen.bmj.com/site/about/guidelines.xhtml

2. Medline

(*Noncommunicable Diseases/ OR exp *Neoplasms/ OR exp *Cardiology/ OR exp *Early Detection of Cancer/ OR exp *Diabetes Mellitus/ OR exp *Cardiovascular Diseases/ OR exp *Cerebrovascular Disorders/ OR exp *"Pulmonary Disease, Chronic Obstructive"/ OR exp *Hypertension/ OR (((non-communicab* OR noncommunicab*) ADJ3 disease*) OR cancer* OR ((heart OR cardiac OR cardiovascul* OR cerebrovascul* OR myocardial*) ADJ3 (disease* OR patient* OR emergenc* OR attack* OR infarct*)) OR cardiolog* OR oncolog* OR diabet* OR (chronic ADJ3 (respirator* OR lung* OR pulmonar*) ADJ3 (failure OR disease*)) OR COPD OR asthma* OR bronchitis OR ((lung* OR pulmonar*) ADJ3 (emphysem*)) OR ((cerebrovascul* OR cerebro-vascul*) ADJ3 accident*) OR stroke OR hypertens* OR (surg* ADJ3 emergenc*)).ti,kf) AND (Developing Countries/ OR exp Africa/ OR Asia/ OR Latin America/ OR ((resource* ADJ3 (limit* OR constrain* OR low)) OR ((low OR middle) ADJ3 income* ADJ3 countr*) OR LMIC* OR (developing ADJ3 (countr* OR world* OR nation*)) OR third-world* OR 3rd-world* OR countries OR Afghan* OR alban* OR Algeria* OR American-somoa* OR Argentin* OR Armeni* OR asia OR AZERBAIJAN* OR Bangladesh* OR Belarus OR Belize OR Benin OR Bhutan* OR Bolivia* OR Bosnia* OR Botswan* OR Brazil* OR Bulgaria* OR Burkina-Faso OR Burundi OR Cabo-Verde* OR Central-African-Republic OR Chad OR China* OR Chinese* OR Colombia* OR Comoros OR Congo* OR Costa-Rica* OR Croatia* OR Cuba* OR Ecuador* OR Eritrea* OR Eswatini* OR Ethiopia* OR Fiji* OR Gabon* OR Gambia* OR Grenada* OR Guinea* OR Guatemal* OR Guyan* OR Haiti* OR Iran* OR Iraq* OR Jamaic* OR Jordan OR Kazakhst* OR DPR-Korea OR North-Korea OR Liberia* OR Latin-Americ* OR Madagasc* OR Malawi* OR Malaysia* OR Maldives* OR Mali OR malese OR Marshall-Island* OR Mexico* OR Mexican OR Micronesia* OR Mozambique* OR Nepal* OR Niger OR Palestin* OR Panama* OR Romania* OR Rwand* OR Samoa* OR Sao-Tome* OR Senegal* OR Sierra-Leon* OR Somali* OR South-Afric* OR Sudan* OR Syria* OR Tajiki* OR Tanzania* OR Togo* OR Tokolau* OR Tonga* OR Uganda* OR Yemen* OR Zimbabwe* OR Angol* OR Banglad* OR Bhutan* OR Bolivia* OR Cabo-Verd* OR Cambodia* OR Cameroon* OR Cote-d-Ivoir* OR Djibout* OR Dominica* OR Egypt* OR El-Salvador* OR (Georgia NOT (usa OR us OR united-states)) OR Ghan* OR Hondur* OR India OR Indones* OR Kenya* OR Kiribati* OR Kosov* OR Kyrgyz* OR PDR-Lao OR Laotian* OR Lebanon* OR Lebanese* OR Lesoth* OR Libya* OR Mauritania* OR Mauritius* OR Micronesia* OR Moldova* OR Mongolia* OR Montenegr* OR Morocco* OR Myanmar* OR Namibia* OR Nauru* OR Nicaragua* OR Nigeria* OR Niue OR Macedoni* OR Pakistan* OR Papua* OR Paraguay* OR Peru OR Philippin* OR Russian-Feder* OR Sao-Tome* OR Serbia* OR Solomon-Island* OR South-Afric* OR Sri-Lank* OR Saint-Lucia* OR Saint-Vincent* OR Sub-Sahara* OR Subsahara* OR Sudan* OR Surinam* OR Swaziland* OR Thailand* OR Timor-Leste OR Tunisia* OR Turkey OR Turkmenist* OR Tuvalu* OR Ukrain* OR Uzbeki* OR Vanuatu* OR Venezuel* OR Vietnam* OR West-Bank OR Gaza OR Zambia* OR africa).ab,ti,kf) AND (*"Patient Acceptance of Health Care"/ OR exp *Health Care Costs/OR *"Quality

For peer review only - http://bmjopen.bmj.com/site/about/guidelines.xhtml

of Health Care"/ OR *"Costs and Cost Analysis"/ OR exp *Health Services Accessibility/ OR *Delivery of Health Care/ OR *Financial Stress/ OR *"Insurance, Health, Reimbursement"/ OR *Health Expenditures/ OR *Healthcare Disparities/ OR *Cost-Benefit Analysis/ OR *Patient Participation/ OR (*Empowerment/ AND *Patients/) OR *Patient Reported Outcome Measures/ OR (((health) ADJ3 (seeking* OR inequalit*)) OR ((health* OR hospital*) ADJ3 (cost* OR expenditur* OR econom*)) OR ((health* OR care) ADJ3 (qualit* OR evaluat* OR access* OR deliver* OR utilization* OR utilisation* OR "use")) OR affordab* OR accessib* OR proximit* OR distance* OR ((financial* OR econom*) ADJ3 (stress OR burden* OR distress* OR pressure* OR strain*)) OR ((opening* OR after OR out-of*) ADJ3 (hour*)) OR in-network* OR reimbursement* OR reputation* OR out-of-pocket* OR ((indirect) ADJ3 (cost* OR expendit*)) OR benefit-incidence* OR ((cost*) ADJ3 (effectiv* OR efficien*)) OR debt* OR indebt* OR (empowerment* ADJ3 (self OR patient*)) OR (patient-reported ADJ3 (outcome* OR experience* OR satisfaction*))).ti,kf) AND (exp *"Health Care Facilities, Manpower, and Services"/ OR (((health* OR care OR public* OR private* OR nonprofit* OR non-profit* OR faith*) ADJ3 (facilit* OR provider* OR service* OR based OR sector)) OR hospital* OR clinic OR ((health*) ADJ3 (center* OR centre* OR post)) OR ((family OR general) ADJ3 (practitioner* OR doctor)) OR ((primary OR secondary OR tertiary OR nursing) ADJ3 (care)) OR specialist OR specialists OR ((home) ADJ3 (care OR nursing OR help OR service* OR treatment*)) OR homecare* OR cascade-of-care* OR pharmacy OR pharmacies).ti,kf) AND 2000:2030.(sa_year) NOT (Editorial/) NOT (news OR congres* OR abstract* OR book* OR chapter* OR dissertation abstract*).pt. NOT (Case Reports/ OR (case-report).ti.)

3. Web of Science

TI=(((((non-communicab* OR noncommunicab*) NEAR/2 disease*) OR cancer* OR ((heart OR cardiac OR cardiovascul* OR cerebrovascul* OR myocardial*) NEAR/2 (disease* OR patient* OR emergenc* OR attack* OR infarct*)) OR cardiolog* OR oncolog* OR diabet* OR (chronic NEAR/2 (respirator* OR lung* OR pulmonar*) NEAR/2 (failure OR disease*)) OR COPD OR asthma* OR bronchitis OR ((lung* OR pulmonar*) NEAR/2 (emphysem*)) OR ((cerebrovascul* OR cerebro-vascul*) NEAR/2 accident*) OR stroke OR hypertens* OR (surg* NEAR/2 emergenc*)))) AND TS=((((resource* NEAR/2 (limit* OR constrain* OR low)) OR ((low OR middle) NEAR/2 income* NEAR/2 countr*) OR LMIC* OR (developing NEAR/2 (countr* OR world* OR nation*)) OR third-world* OR 3rd-world* OR countries OR Afghan* OR albania* OR Algeria* OR American-somoa* OR Argentin* OR Armeni* OR asia OR AZERBAIJAN* OR Bangladesh* OR Belarus OR Belize OR Benin OR Bhutan* OR Bolivia* OR Bosnia* OR Botswan* OR Brazil* OR Bulgaria* OR Burkina-Faso OR Burundi OR Cabo-Verde* OR Central-African-Republic OR Chad OR China* OR Chinese* OR Colombia* OR Comoros OR Congo* OR Costa-Rica* OR Croatia* OR Cuba* OR Ecuador* OR Eritrea* OR Eswatini* OR Ethiopia* OR Fiji* OR Gabon* OR Gambia* OR

For peer review only - http://bmjopen.bmj.com/site/about/guidelines.xhtml

Grenada* OR Guinea* OR Guatemal* OR Guyan* OR Haiti* OR Iran* OR Iraq* OR Jamaic* OR Jordan OR Kazakhst* OR DPR-Korea OR North-Korea OR Liberia* OR Latin-Americ* OR Madagasc* OR Malawi* OR Malaysia* OR Maldives* OR Mali OR malese OR Marshall-Island* OR Mexico* OR Mexican OR Micronesia* OR Mozambique* OR Nepal* OR Niger OR Palestin* OR Panama* OR Romania* OR Rwand* OR Samoa* OR Sao-Tome* OR Senegal* OR Sierra-Leon* OR Somali* OR South-Afric* OR Sudan* OR Syria* OR Tajiki* OR Tanzania* OR Togo* OR Tokolau* OR Tonga* OR Uganda* OR Yemen* OR Zimbabwe* OR Angol* OR Banglad* OR Bhutan* OR Bolivia* OR Cabo-Verd* OR Cambodia* OR Cameroon* OR Cote-d-Ivoir* OR Djibout* OR Dominica* OR Egypt* OR El-Salvador* OR (Georgia NOT (usa OR us OR united-states)) OR Ghan* OR Hondur* OR India OR Indones* OR Kenya* OR Kiribati* OR Kosov* OR Kyrgyz* OR PDR-Lao OR Laotian* OR Lebanon* OR Lebanese* OR Lesoth* OR Libya* OR Mauritania* OR Mauritius* OR Micronesia* OR Moldova* OR Mongolia* OR Montenegr* OR Morocco* OR Myanmar* OR Namibia* OR Nauru* OR Nicaragua* OR Nigeria* OR Niue OR Macedoni* OR Pakistan* OR Papua* OR Paraguay* OR Peru OR Philippin* OR Russian-Feder* OR São-Tomé* OR Serbia* OR Solomon-Island* OR South-Afric* OR Sri-Lank* OR Saint-Lucia* OR Saint-Vincent* OR Sub-Sahara* OR Subsahara* OR Sudan* OR Surinam* OR Swaziland* OR Thailand* OR Timor-Leste OR Tunisia* OR Turkey OR Turkmenist* OR Tuvalu* OR Ukrain* OR Uzbeki* OR Vanuatu* OR Venezuel* OR Vietnam* OR West-Bank OR Gaza OR Zambia* OR africa))) AND TI=(((((health) NEAR/2 (seeking* OR inequalit*)) OR ((health* OR hospital*) NEAR/2 (cost* OR expenditur* OR econom*)) OR ((health* OR care) NEAR/2 (qualit* OR evaluat* OR access* OR deliver* OR utilization* OR utilisation* OR "use")) OR affordab* OR access OR accessib* OR proximit* OR distance* OR ((financial* OR econom*) NEAR/2 (stress OR burden* OR distress* OR pressure* OR strain*)) OR ((opening* OR after OR out-of*) NEAR/2 (hour*)) OR in-network* OR reimbursement* OR reputation* OR out-of-pocket* OR ((indirect) NEAR/2 (cost* OR expendit*)) OR benefit-incidence* OR ((cost*) NEAR/2 (effectiv* OR efficien*)) OR debt* OR indebt* OR (empowerment* NEAR/3 (self OR patient*)) OR (patient-reported NEAR/3 (outcome* OR experience* OR satisfaction*)))) AND ((((health* OR care OR public* OR private* OR nonprofit* OR non-profit* OR faith*) NEAR/2 (facilit* OR provider* OR service* OR based OR sector)) OR hospital* OR clinic OR ((health*) NEAR/2 (center* OR centre* OR post)) OR ((family OR general) NEAR/2 (practitioner* OR doctor)) OR ((primary OR secondary OR tertiary OR nursing) NEAR/2 (care)) OR specialist OR specialists OR ((home) NEAR/2 (care OR nursing OR help OR service* OR treatment*)) OR homecare* OR cascade-of-care* OR pharmacy OR pharmacies))) AND py=(2000-2030) AND DT=(Article OR Review OR Letter OR Early Access) NOT TI=(case-report)

4. Econlit

TI(non-communicab* OR noncommunicab* OR cancer* OR cardiac*OR cardiolog* OR oncolog* OR diabet* OR COPD OR asthma* OR bronchitis OR stroke OR hypertens*) **AND** ((((health* OR care OR

For peer review only - http://bmjopen.bmj.com/site/about/guidelines.xhtml

For peer review only

public* OR private* OR nonprofit* OR non-profit* OR faith*) N/2 (facilit* OR provider* OR service* OR based OR sector)) OR hospital* OR clinic OR ((health*) N/2 (center* OR centre* OR post)) OR ((family OR general) N/2 (practitioner* OR doctor)) OR ((primary OR secondary OR tertiary OR nursing) N/2 (care)) OR specialist OR specialists OR ((home) N/2 (care OR nursing OR help OR service* OR treatment*)) OR homecare* OR cascade-of-care* OR pharmacy OR pharmacies)) NOT PT (news OR comment* OR editorial* OR congres* OR abstract* OR book* OR chapter* OR dissertation abstract*) **AND** AB,TI((LMIC* OR third-world* OR 3rd-world* OR Afghan* OR albania* OR Algeria* OR American-somoa* OR Argentin* OR Armeni* OR asia OR AZERBAIJAN* OR Bangladesh* OR Belarus OR Belize OR Benin OR Bhutan* OR Bolivia* OR Bosnia* OR Botswan* OR Brazil* OR Bulgaria* OR Burkina-Faso OR Burundi OR Cabo-Verde* OR Central-African-Republic OR Chad OR China* OR Chinese* OR Colombia* OR Comoros OR Congo* OR Costa-Rica* OR Croatia* OR Cuba* OR Ecuador* OR Eritrea* OR Eswatini* OR Ethiopia* OR Fiji* OR Gabon* OR Gambia* OR Grenada* OR Guinea* OR Guatemal* OR Guyan* OR Haiti* OR Iran* OR Iraq* OR Jamaic* OR Jordan OR Kazakhst* OR DPR-Korea OR North-Korea OR Liberia* OR Latin-Americ* OR Madagasc* OR Malawi* OR Malaysia* OR Maldives* OR Mali OR malese OR Marshall-Island* OR Mexico* OR Mexican OR Micronesia* OR Mozambique* OR Nepal* OR Niger OR Palestin* OR Panama* OR Romania* OR Rwand* OR Samoa* OR Sao-Tome* OR Senegal* OR Sierra-Leon* OR Somali* OR South-Afric* OR Sudan* OR Syria* OR Tajiki* OR Tanzania* OR Togo* OR Tokolau* OR Tonga* OR Uganda* OR Yemen* OR Zimbabwe* OR Angol* OR Banglad* OR Bhutan* OR Bolivia* OR Cabo-Verd* OR Cambodia* OR Cameroon* OR Cote-d-Ivoir* OR Djibout* OR Dominica* OR Egypt* OR El-Salvador* OR (Georgia NOT (usa OR us OR united-states)) OR Ghan* OR Hondur* OR India OR Indones* OR Kenya* OR Kiribati* OR Kosov* OR Kyrgyz* OR PDR-Lao OR Laotian* OR Lebanon* OR Lebanese* OR Lesoth* OR Libya* OR Mauritania* OR Mauritius* OR Micronesia* OR Moldova* OR Mongolia* OR Montenegr* OR Morocco* OR Myanmar* OR Namibia* OR Nauru* OR Nicaragua* OR Nigeria* OR Niue OR Macedoni* OR Pakistan* OR Papua* OR Paraguay* OR Peru OR Philippin* OR Russian-Feder* OR São-Tomé* OR Serbia* OR Solomon-Island* OR South-Afric* OR Sri-Lank* OR Saint-Lucia* OR Saint-Vincent* OR Sub-Sahara* OR Subsahara* OR Sudan* OR Surinam* OR Swaziland* OR Thailand* OR Timor-Leste OR Tunisia* OR Turkey OR Turkmenist* OR Tuvalu* OR Ukrain* OR Uzbeki* OR Vanuatu* OR Venezuel* OR Vietnam* OR West-Bank OR Gaza OR Zambia* OR africa)) NOT PT (letter* OR news OR comment* OR editorial* OR congres* OR abstract* OR book* OR chapter* OR dissertation abstract*)

5. Global Index Medicus

TI:("non communicable" OR noncommunicable* OR cancer* OR cardiovascular* OR diabet* OR COPD OR asthma* OR bronchit* OR stroke OR hypertens* OR "heart disease" OR "cardiac disease" OR cerebrovascular* OR myocardial* OR "heart attack" OR "heart infarct" OR "cardiac attack" OR "cardiac

For peer review only - http://bmjopen.bmj.com/site/about/guidelines.xhtml

infarct" OR cardiolog* OR oncolog*) AND ti:("health seeking" OR "health inequality" OR "patient-acceptance" OR "health cost" OR "health evaluation" OR access OR affordabilit* OR "cost analysis" OR accessibility OR "financial stress" OR "patient reported" OR "walking distance" OR "out of pocket" OR "out-of-pocket" OR debt OR "patient empowerment" OR "self empowerment" OR "in network" OR "cost effectiveness" OR "cost efficiency" OR "patient reported outcome") AND TI:("health service" OR "health facility" OR "health provider" OR "health centre" OR "health center" OR "care service" OR "care facility" OR "care provider" OR "public service" OR "public facility" OR "public provider" OR "private service" OR "private facility" OR "private provider" OR hospital* OR clinic* OR specialist* OR homecare* OR non-profit* OR "family practitioner" OR "family doctor" OR "general practitioner")

6. Google Scholar

non-communicable|cancer|cardiovascular|diabetes|COPD|asthma|stroke|hypertension LMIC|'low middle income countries' 'health seeking|cost|quality|access|utilization' 'health provider|facility|center|centre|service'|hospital|clinic|pharmacy

For peer review only - http://bmjopen.bmj.com/site/about/guidelines.xhtml

PRISMA 2020 Checklist

Section and Topic	Item #	Checklist item	Location where item is reported
**TITLE**			
Title	1	Identify the report as a systematic review.	Yes
**ABSTRACT**			
Abstract	2	See the PRISMA 2020 for Abstracts checklist.	Yes
**INTRODUCTION**			
Rationale	3	Describe the rationale for the review in the context of existing knowledge.	Yes
Objectives	4	Provide an explicit statement of the objective(s) or question(s) the review addresses.	Yes
**METHODS**			
Eligibility criteria	5	Specify the inclusion and exclusion criteria for the review and how studies were grouped for the syntheses.	Yes
Information sources	6	Specify all databases, registers, websites, organisations, reference lists and other sources searched or consulted to identify studies. Specify the date when each source was last searched or consulted.	Yes
Search strategy	7	Present the full search strategies for all databases, registers and websites, including any filters and limits used.	Yes
Selection process	8	Specify the methods used to decide whether a study met the inclusion criteria of the review, including how many reviewers screened each record and each report retrieved, whether they worked independently, and if applicable, details of automation tools used in the process.	Yes
Data collection process	9	Specify the methods used to collect data from reports, including how many reviewers collected data from each report, whether they worked independently, any processes for obtaining or confirming data from study investigators, and if applicable, details of automation tools used in the process.	Yes
Data items	10a	List and define all outcomes for which data were sought. Specify whether all results that were compatible with each outcome domain in each study were sought (e.g. for all measures, time points, analyses), and if not, the methods used to decide which results to collect.	Yes
	10b	List and define all other variables for which data were sought (e.g. participant and intervention characteristics, funding sources). Describe any assumptions made about any missing or unclear information.	Yes
Study risk of bias assessment	11	Specify the methods used to assess risk of bias in the included studies, including details of the tool(s) used, how many reviewers assessed each study and whether they worked independently, and if applicable, details of automation tools used in the process.	Yes
Effect measures	12	Specify for each outcome the effect measure(s) (e.g. risk ratio, mean difference) used in the synthesis or presentation of results.	Yes
Synthesis methods	13a	Describe the processes used to decide which studies were eligible for each synthesis (e.g. tabulating the study intervention characteristics and comparing against the planned groups for each synthesis (item #5)).	Yes
	13b	Describe any methods required to prepare the data for presentation or synthesis, such as handling of missing summary statistics, or data conversions.	Yes
	13c	Describe any methods used to tabulate or visually display results of individual studies and syntheses.	Yes
	13d	Describe any methods used to synthesize results and provide a rationale for the choice(s). If meta-analysis was performed, describe the model(s), method(s) to identify the presence and extent of statistical heterogeneity, and software package(s) used.	Yes
	13e	Describe any methods used to explore possible causes of heterogeneity among study results (e.g. subgroup analysis, meta-regression).	NA
	13f	Describe any sensitivity analyses conducted to assess robustness of the synthesized results.	NA
Reporting bias assessment	14	Describe any methods used to assess risk of bias due to missing results in a synthesis (arising from reporting biases).	Yes
Certainty	15	Describe any methods used to assess certainty (or confidence) in the body of evidence for an outcome.	Yes

For peer review only - http://bmjopen.bmj.com/site/about/guidelines.xhtml

PRISMA 2020 Checklist

Section and Topic	Item #	Checklist item	Location where item is reported
assessment			
**RESULTS**			
Study selection	16a	Describe the results of the search and selection process, from the number of records identified in the search to the number of studies included in the review, ideally using a flow diagram.	
	16b	Cite studies that might appear to meet the inclusion criteria, but which were excluded, and explain why they were excluded.	
Study characteristics	17	Cite each included study and present its characteristics.	
Risk of bias in studies	18	Present assessments of risk of bias for each included study.	
Results of individual studies	19	For all outcomes, present, for each study: (a) summary statistics for each group (where appropriate) and (b) an effect estimate and its precision (e.g. confidence/credible interval), ideally using structured tables or plots.	
Results of syntheses	20a	For each synthesis, briefly summarise the characteristics and risk of bias among contributing studies.	
	20b	Present results of all statistical syntheses conducted. If meta-analysis was done, present for each the summary estimate and its precision (e.g. confidence/credible interval) and measures of statistical heterogeneity. If comparing groups, describe the direction of the effect.	
	20c	Present results of all investigations of possible causes of heterogeneity among study results.	
	20d	Present results of all sensitivity analyses conducted to assess the robustness of the synthesized results.	
Reporting biases	21	Present assessments of risk of bias due to missing results (arising from reporting biases) for each synthesis assessed.	
Certainty of evidence	22	Present assessments of certainty (or confidence) in the body of evidence for each outcome assessed.	
**DISCUSSION**			
Discussion	23a	Provide a general interpretation of the results in the context of other evidence.	
	23b	Discuss any limitations of the evidence included in the review.	
	23c	Discuss any limitations of the review processes used.	
	23d	Discuss implications of the results for practice, policy, and future research.	
**OTHER INFORMATION**			
Registration and protocol	24a	Provide registration information for the review, including register name and registration number, or state that the review was not registered.	Yes
	24b	Indicate where the review protocol can be accessed, or state that a protocol was not prepared.	Yes
	24c	Describe and explain any amendments to information provided at registration or in the protocol.	Yes
Support	25	Describe sources of financial or non-financial support for the review, and the role of the funders or sponsors in the review.	Yes
Competing interests	26	Declare any competing interests of review authors.	Yes
Availability of data, code and other materials	27	Report which of the following are publicly available and where they can be found: template data collection forms; data extracted from included studies; data used for all analyses; analytic code; any other materials used in the review.	Yes

For peer review only - http://bmjopen.bmj.com/site/about/guidelines.xhtml

From: Page MJ, McKenzie JE, Bossuyt PM, Boutron I, Hoffmann TC, Mulrow CD, et al. The PRISMA 2020 statement: an updated guideline for reporting systematic reviews. BMJ 2021;372:n71. doi: 10.1136/bmj.n71

PRISMA 2020 Checklist

For more information, visit: http://www.prisma-statement.org/

For peer review only

BMJ Open

Health seeking behaviours and private sector delivery of care for non-communicable diseases in low- and middle-income countries: protocol for a systematic review

Journal:	*BMJ Open*
Manuscript ID	bmjopen-2022-066213.R1
Article Type:	Protocol
Date Submitted by the Author:	19-Apr-2023
Complete List of Authors:	Brindley, Callum; Erasmus University Rotterdam<br>Wijemunige, Nilmini; Erasmus University Rotterdam; Institute for Health Policy<br>Dieteren, Charlotte; Erasmus University Rotterdam<br>Bom, Judith; Erasmus University Rotterdam<br>Engel, Maarten; Erasmus University Rotterdam<br>Meessen, Bruno; World Health Organization<br>Bonfrer, IEJ ; Erasmus Universiteit Rotterdam,
**Primary Subject Heading**:	Global health
Secondary Subject Heading:	Health economics, Health services research
Keywords:	Health economics < HEALTH SERVICES ADMINISTRATION & MANAGEMENT, Health policy < HEALTH SERVICES ADMINISTRATION & MANAGEMENT, Organisation of health services < HEALTH SERVICES ADMINISTRATION & MANAGEMENT, Quality in health care < HEALTH SERVICES ADMINISTRATION & MANAGEMENT

SCHOLARONE™
Manuscripts

Health seeking behaviours and private sector delivery of care for non-communicable diseases in low- and middle-income countries: protocol for a systematic review

Authors list:

Callum Brindley[1,2]*, Nilmini Wijemunige[1,3], Charlotte Dieteren[1,2], Judith Bom[1,2], Maarten Engel[4], Bruno Meessen[5] and Igna Bonfrer[1,2]

Affiliations:

[1] Erasmus School of Health Policy and Management, Erasmus University Rotterdam, Rotterdam, The Netherlands

[2] Erasmus Centre for Health Economics, Erasmus University Rotterdam, Rotterdam, The Netherlands

[3] Institute for Health Policy, Colombo, Sri Lanka

[4] Medical Library, Erasmus Medical Centre, Erasmus University Rotterdam, Rotterdam, The Netherlands

[5] The World Health Organization, Geneva, Switzerland

*Correspondence author P.O. Box 1738, 3000 DR Rotterdam, The Netherlands. Email: brindley@eshpm.eur.nl

Tel: +31 10 408 8555

Word Count: 1915

For peer review only

Abstract

Introduction The burden of noncommunicable diseases (NCDs) has increased substantially in low- and middle-income countries (LMICs) and adapting health service delivery models to address this remains a challenge. Many NCD patients seek private care at different points in their encounters with the health system, but the determinants and outcomesof these choices are insufficiently understood. The proposed systematic review will help inform the governance of mixed health systems towards achieving the goal of universal health coverage. This protocol details our intended methodological and analytical approaches, based on the Preferred Reporting Items for Systematic Reviews and Meta-Analyses (PRISMA)

Methods and analysis Following the PRISMA approach, this systematic review will develop a descriptive synthesis of the determinants and outcomesof private health care utilisation for NCDs in LMICs. The databases Embase, Medline, Web of Science Core Collection, EconLit, Global Index Medicus and Google Scholar will be searched for relevant studies published in English between period 1 January 2010 and 30 June 2022 with additional searching of reference lists. The study selection process will involve a title-abstract and full text review, guided by clearly defined inclusion and exclusion criteria. A quality and risk of bias assessment will be done for each study using the Mixed Methods Appraisal Tool (MMAT).

Ethics and dissemination Ethical approval is not required because this review is based on data collected from publicly available materials. The results will be published in a peer-reviewed journal and presented at related scientific events.

PROSPERO registration number CRD42022340059

Keywords health seeking behaviour, private sector, noncommunicable diseases, cardiovascular disease, diabetes, respiratory diseases, cancer, low- and middle-income countries

Strengths and limitations of this study

- This is a systematic review protocol that proposes a timely contribution to the challenge of adapting health care service delivery to prevent, treat and manage NCDs in LMICs.
- We define the private sector broadly to capture its breadth and complexity.
- Although we will consider all LMICs, this systematic review is limited to studies published in English, so does not include relevant literature in other languages.
- We limit our attention to literature from the past decade which we contend is most relevant given recent changes in disease burden, risk factors and demographics in LMICs.
- We will not perform a meta-analysis because we expect highly diverse study characteristics, including design type, setting, intervention and outcome.

For peer review only - http://bmjopen.bmj.com/site/about/guidelines.xhtml

For peer review only

For peer review only - http://bmjopen.bmj.com/site/about/guidelines.xhtml

1. Introduction

The rising burden of non-communicable diseases (NCDs) in low- and middle-income countries (LMICs) [1,2] calls for policy responses that engage the whole health system, including private providers. While the appropriate role of the private sector in the delivery of health care has been heavily debated [3–6], it is well known that patients seek private care at different points in their encounters with the health system.[7–10] Research into the reasons for visiting private providers and the comparative quality of this care in LMICs has largely focused on infectious diseases, and maternal and child health.[11]

With regard to NCDs, the determinants and outcomesof private health care utilisation in LMICs remains insufficiently understood. Importantly, NCDs have multiple etiologies (including behavioural, environmental, and genetic) that require an organisation of services and delivery of care that is different from that of acute illnesses.[12] Specifically, a focus on prevention and primary care that empowers patients and prioritises information sharing and coordination across levels of care and providers.[13,12] This is imperative for the achievement of Sustainable Development Goal (SDG) target 3.4 – a one-third reduction in premature mortality from NCDs by 2030.

Adapting health service delivery models to respond to NCDs remains a challenge.[14] In many LMICs, national NCD strategies tend to focus on the public sector to the exclusion of the private sector despite the two being entwined elements of the whole health system.[15–17] Many LMICs with universal health coverage (UHC) benefits packages also only offer limited coverage for NCDs, such that patients must pay out-of-pocket even in public health facilities.[18] The risk is that this discourages and distorts health seeking behaviours, leading to ineffective care, poor health outcomes and catastrophic health spending.[18,19] Understanding better the reality of health seeking behaviours for NCDs, including the possible role already taken by private providers, will be key for the design and implementation of appropriate strategies and interventions, across the different building blocks of the health system.[20]

This study aims to synthesize the scattered evidence on how people with NCDs choose their healthcare providers in LMICs, and the outcomes of these choices, with a focus on the understudied private sector. In particular, we concentrate on the contextual and individual factors that influence provider choice, patterns of utilisation, quality of care and financial protection. We use the notion of the patient journey and draw on existing conceptual frameworks of health seeking behaviour and the facilitators and barriers to accessing healthcare.[21–24] This systematic review has been initiated by the World Health Organization's (WHO) Department of Noncommunicable Disease, and Department of Health System Financing and Governance. It follows work by a WHO Advisory Group on Governance of the

For peer review only - http://bmjopen.bmj.com/site/about/guidelines.xhtml

Private Sector and a systematic review of private sector delivery of quality care for maternal, newborn and child health in LMICs.[25,26] This review will contribute to an understanding of health seeking behaviour for NCDs in the private sector in LMICs, which will in turn help inform the governance of mixed health systems in the pursuit of UHC and the achievement of the SDGs.

2. Methods and analysis

2.1. Aim and research questions

This systematic review on the determinants and outcomes of private health care utilisation is built around the questions – *What factors influence private health care seeking behaviour for individuals with NCDs in LMICs? From whom is health care obtained, what are the patterns of utilization, what are the determinants that influence the use of private as opposed to public health care, and what are the outcomes of this health care seeking?* This review allows us to determine the size and nature of the current literature and to identify major knowledge gaps that relate to mixed health care systems with entwined public and private providers of NCD care in LMICs.

2.2. Study design and protocol

This study is guided by the Preferred Reporting Items for Systematic Reviews and Meta-Analyses (PRISMA) and its extension for literature searches PRISMA-S.[27,28] PRISMA is a systematic approach to map and synthesise existing evidence, identify knowledge gaps and inform future research. This review was registered with PROSPERO (CRD42022340059) on 15 June 2022. We checked that there were no current or in-progress systematic reviews on the same topic by searching PROSPERO, the Research Registry, and the Open Science Framework.

2.3. Inclusion/exclusion criteria

Table 1 outlines the Populations, Interventions, Control, Outcomes, Timeframe, Setting (PICOTS) criteria to be used. We focus on adults aged 18 years or older and define the scope of health care to encompass the prevention, diagnosis, treatment and/or management of disease at all level (e.g. primary health clinic to hospital). We restrict our search to four groups of NCDs responsible for over 80% of all premature NCD deaths and identified by SDG target 3.4. Specifically, cardiovascular

For peer review only - http://bmjopen.bmj.com/site/about/guidelines.xhtml

For peer review only

diseases, cancers, respiratory diseases and diabetes. With regard to cancers, we limit our scope to the top five cancers with the greatest disease burden for each sex.[29] We use the World Health Organization's operational definition of the private health sector as the individuals and organizations that are neither owned nor directly controlled by governments, and are involved in the provision of health services (i.e. formal and informal providers as well as for-profit and non-profit entities.[30] Our outcomes of interest are the determinants and outcomesof health seeking behaviour at both the individual and collective level, which are described in more detailed under the search elements and key terms. We focus on the timeframe from January 2010 to June 2022, which is defined by large increases in the NCD disease burden in LMICs and the first High-Level Meeting of the UN General Assembly on the Prevention and Control of Non-communicable Diseases.[29,31] This also avoids duplication of a broader systematic review into the private provision of health services in LMICs conducted in 2011.[32] Lastly, the study looks at settings in LMICs as defined by the World Bank classification for 2022 (i.e. countries with a gross national income per capita of $4,095 or less).[33]

Table 1 - PICOTS criteria used in the systematic review

Population	Adults aged 18 years or older and households at risk, or diagnosed with at least one of the following non-communicable diseases: cardiovascular diseases, diabetes, chronic respiratory diseases or cancers (tracheal, bronchus and lung; colon and rectum; pancreas; stomach; breast and prostate)
Interventions	Private sector provision of health care services all levels (e.g. primary health clinic to hospital) that involves the prevention, diagnosis, treatment and/or management of non-communicable disease
Control	Not applicable
Outcomes	Determinants and outcomes of health care seeking behaviour, at the individual and population level
Timeframe	1 January 2010 to 30 June 2022
Setting	Low-income and middle-income countries following the World Bank 2022 classification [33]

For inclusion, studies must adhere to all elements defined in Table 1. They must be in English and published in a peer-reviewed journal. We will include both qualitative and quantitative studies, including randomized and non-randomized study designs (i.e., cross-sectional, case-control and cohort observational studies). Editorials, commentaries, reviews, and protocols will be excluded.

For peer review only - http://bmjopen.bmj.com/site/about/guidelines.xhtml

Studies must address private sector health care and may do so in a comparison with the public sector. However, we will exclude studies that only report aggregated data (i.e., private and public health sector outcome data combined). Lastly, we will also exclude vaccination programmes associated with NCDs (e.g., human papillomavirus) because they are government-led, as well as settings with irregular contextual circumstances (e.g., conflict, economic and political crises) since they are less likely to offer generalisable insights.

2.4. Search strategy

Our search strategy was developed with the assistance of an information specialist (ME). The following electronic databases will be searched: Embase, Medline, Web of Science, Econlit, Global Index Medicus.[34] We will also search Google Scholar and download the 200 most relevant references in line with common practice.[35] Table 2 provides the search elements and key terms we will use. Specifically, it contains terms for 1) non communicable diseases, 2) health care providers in LMICs, and 3) the determinants and outcomesof health seeking behaviour and related terms. The search was developed in Embase, optimized for sensitivity, then translated to other databases following the method described by Bramer et al 2018.[36] The search strategies for Embase and Medline use relevant thesaurus terms from Emtree and Medical Subject Headings (MeSH) respectively. In all databases, the terms will be searched in titles, author keywords and major index terms; the search element for setting (health care providers in LMICs) will also be searched in abstracts. The full search strategies of all databases are provided in the Supplementary Material. References will be imported into EndNote and duplicates removed.[37] Reference lists of revelvant reviews and all included articles will also be screened for potential additional sources missed by the search. We do not plan to contact authors or subject expects.

Table 2 - Search elements and key terms

Search element	Key terms
What: noncommunicable diseases	noncommunicable diseases; cardiovascular disease; heart attack; stroke; myocardial infarction; hypertension; diabetes; cancer (top 5 for each sex: lung, breast, colorectum, prostate, stomach, liver, cervix, thyroid); chronic respiratory disease; chronic obstructive pulmonary disease; asthma)

For peer review only - http://bmjopen.bmj.com/site/about/guidelines.xhtml

Where: health care providers in low- and middle-income countries	hospital, health clinic, community health centre, health centre, health post, family practitioner, general practitioner, primary care, secondary care, tertiary care, specialist, home care, nursing home, pharmacy, public provider, private provider, nonprofit provider, faith-based provider, low-income countries, middle-income countries, lower-middle income countries, upper-middle income countries, list of all LMIC countries
Why and what happens: determinants and outcomesof health care decisions / health seeking behaviour	quality; cost; affordability; accessibility; proximity; distance; opening hours; in-network provider; reimbursement; reputation; health service delivery; health outcomes; utilization; out-of-pocket payment; indirect costs; catastrophic health care expenditure; debt; health inequalities; health information; supply; fee for service; empowerment; patient reported experience

2.5. Screening process

The articles resulting from the search will be screened by a team of two pairs (CB-JB & NW-CD) working in the Rayyan Reference Manager.[38,39] After removing duplicates, each article will be title-abstract screened independently by two individuals using the eligibility criteria described above. To reduce bias, author names will not be visible during this screening stage. Before commencing screening by pairs, we will also conduct a pilot phase in which a random selection of 100 articles will be reviewed and discussed jointly to harmonise our approach. Articles adhering to all criteria will be selected for full-text review. Title-abstracts will be screened in three lots. Any conflicts will be reviewed within the pair at the conclusion of reviewing each lot, to allow for further harmonization within the pair. If an inclusion/exclusion decision cannot be made based on the title and abstract, the article will be advanced to full-text screening. In the case of a conflicting inclusion/exclusion decision, the other pair in the team will also review the article and the supervising researcher (IB) will provide a final adjudication if needed. Articles selected for full-text screening will be reviewed by two individuals and a decision to exclude at this stage will be documented. Confirmed texts for inclusion in the systematic review will be advanced to the next stage of data extraction.

For peer review only - http://bmjopen.bmj.com/site/about/guidelines.xhtml

2.6. Data extraction

We will pilot and refine a data extraction framework including **study details** such as authors, title and year; and **study characteristics** such as research design, sample size, studied country, disease and provider type. We will extract the **determinants of selecting a health care provider**, grouped into three categories: 1) *individual factors* such as demographics and health; 2) *contextual factors* such availability and accessibility of health care; and 3) *perception of providers* such as quality and competence. Lastly, we extract **outcomes of selecting a health care provider**, which we will group into three categories: 1) *patterns of utilization* including characteristics of patients and equity issues; 2) *quality of care* including the Donabedian model[40] domains of process and outcomes as well as patient satisfaction and empowerment; and 3) *spending and financial protection* as captured by indicators such as catastrophic health spending and impoverishment. Each full-text article included in the systematic review will be read by two individuals with one person taking the lead to extract the data, and the second person reviewing this work.

In the sub-section 'Quality assessment', we explain how we will use the Mixed Methods Appraisal Tool to assess the methodological quality

2.7. Quality assessment

We will use the Mixed Methods Appraisal Tool (MMAT) to assess the methodological quality and risk of bias of the reviewed full-text articles.[41] MMAT is a critical appraisal tool intended for systematic reviews that include diverse study designs (i.e., quantitative, qualitative and mixed methods). Each full-text article will be assessed by a team of two pairs (CB-JB & NW-CD) with one person taking the lead and the second person reviewing for completeness and accuracy. Discrepancies and disagreements will be resolved through discussion.

2.8. Synthesis

The team will meet to discuss and analyse the data extracted, which we will consolidate using a descriptive synthesis including a summary of the evidence, gaps, and limitations. As described above in the data extraction, we will synthesise the results into three categories of determinants of selecting a health care provider (individual factors, contextual factors and perception of providers) and three categories of outcomes of selecting a health care provider(patterns of utilization, quality of care, and spending and financial protection). We will also develop a synthesis of the results that concern the

For peer review only - http://bmjopen.bmj.com/site/about/guidelines.xhtml

interaction of health seeking determinants andoutcomes. A flow diagram of the inclusion/exclusion pathways and the descriptive statistics of the included studies and their outcomes will be developed to complement the analysis. We will not perform a meta-analysis because we expect highly diverse study characteristics, including design type, setting, intervention and outcome.

2.9. Patient and public involvement

None

3. Ethics and dissemination

Ethical approval is not required because this is a review and collection of data based on publicly available materials. The results will be presented at events attended by policy makers, academics and health care practitioners from LMICs, and later published in a topic relevant journal. Understanding the reality of health seeking behaviours for NCDs will be key for the design and implementation of interventions to strengthen the building blocks of the health system: from regulation of the health workforce and provider payment reform to benefit package design and the digitalisation of patient files. This systematic review will be a timely contribution to the challenge of adapting health care service delivery to prevent, treat and manage NCDs in LMICs.

For peer review only - http://bmjopen.bmj.com/site/about/guidelines.xhtml

For peer review only

Acknowledgements We wish to acknowledge the valuable contributions of Annalise Belloni, Owen O'Donnell, Tom van Ourti and Eddy van Doorslaer who provided input for this systematic review protocol.

Contributions IB, BM, ME and CB conceptualised the protocol. CB wrote the first draft with input from NW, CD, JB, ME and IB. All authors contributed to subsequent revisions and approved the protocol prior to its submission. All authors are accountable for the research presented.

Funding This work was supported jointly by the Non-Communicable Disease Department, and Health System Financing and Governance Department of the World Health Organization (Grant number 202758205). The content of this study is the sole responsibility of the authors and does not represent the official views of World Health Organization.

Competing interests None declared

Patient consent for publication Not applicable

Provenance and peer review Not commissioned, externally peer reviewed.

Data availability statement Data sharing not applicable as no datasets generated and/or analysed for this study

Supplementary material This content has been supplied by the author(s). It has not been vetted by BMJ Publishing Group Limited (BMJ) and may not have been peer-reviewed. Any opinions or recommendations discussed are solely those of the author(s) and are not endorsed by BMJ. BMJ disclaims all liability and responsibility arising from any reliance placed on the content. Where the content includes any translated material, BMJ does not warrant the accuracy and reliability of the translations (including but not limited to local regulations, clinical guidelines, terminology, drug names and drug dosages), and is not responsible for any error and/or omissions arising from translation and adaptation or otherwise.

Open access This is an open access article distributed in accordance with the Creative Commons Attribution Non Commercial (CC BY-NC 4.0) license, which permits others to distribute, remix, adapt, build upon this work non-commercially, and license their derivative works on different terms, provided the original work is properly cited, appropriate credit is given, any changes made indicated, and the use is non-commercial. See: http://creativecommons.org/licenses/by-nc/4.0/.

ORCID iDs

Callum Brindley https://orcid.org/0000-0002-5478-3974

Nilmini Wijemunige https://orcid.org/0000-0002-2241-3194

Charlotte Dieteren https://orcid.org/0000-0002-7809-1657

Judith Bom https://orcid.org/0000-0003-4477-7594

Maarten Engel https://orcid.org/0000-0002-1774-7042

Bruno Meessen https://orcid.org/0000-0002-0359-8621

Igna Bonfrer https://orcid.org/0000-0002-8570-0393

For peer review only

References

1 Bennett JE, Stevens GA, Mathers CD, *et al.* NCD Countdown 2030: worldwide trends in non-communicable disease mortality and progress towards Sustainable Development Goal target 3.4. *The Lancet* 2018;**392**:1072–88. doi:10.1016/S0140-6736(18)31992-5

2 Bukhman G. The Lancet NCDI Poverty Commission: bridging a gap in universal health coverage for the poorest billion. *The Lancet* 2020;**396**:991–1044. doi:10.1016/S0140-6736(20)31907-3

3 World Health Organization. *The World Health Report 2000. Health systems: improving performance*. Geneva, Switzerland: 2000.

4 World Bank. *World Development Report 2004: Making Services Work for Poor People*. Washington, DC, United States: : The World Bank 2003. doi:10.1596/0-8213-5468-X

5 Marriott A. Blind Optimism: Challenging the myths about private health care in poor countries. Oxfam International 2009. https://policy-practice.oxfam.org/resources/blind-optimism-challenging-the-myths-about-private-health-care-in-poor-countrie-114093/

6 Horton R, Clark S. The perils and possibilities of the private health sector. *The Lancet* 2016;**388**:540–1. doi:10.1016/S0140-6736(16)30774-7

7 Patouillard E, Goodman CA, Hanson KG, *et al.* Can working with the private for-profit sector improve utilization of quality health services by the poor? A systematic review of the literature. *Int J Equity Health* 2007;**6**:17. doi:10.1186/1475-9276-6-17

8 Berendes S, Heywood P, Oliver S, *et al.* Quality of private and public ambulatory health care in low and middle income countries: Systematic review of comparative studies. *PLoS Med* 2011;**8**:12. doi:10.1371/journal.pmed.1000433

9 Basu S, Andrews J, Kishore S, *et al.* Comparative Performance of Private and Public Healthcare Systems in Low- and Middle-Income Countries: A Systematic Review. *PLOS Med* 2012;**9**:e1001244. doi:10.1371/journal.pmed.1001244

10 Devi R, Kanitkar K, Narendhar R, *et al.* A Narrative Review of the Patient Journey Through the Lens of Non-communicable Diseases in Low- and Middle-Income Countries. *Adv Ther* 2020;**37**:4808–30. doi:10.1007/s12325-020-01519-3

For peer review only - http://bmjopen.bmj.com/site/about/guidelines.xhtml

11 Grépin KA. Private Sector An Important But Not Dominant Provider Of Key Health Services In Low- And Middle-Income Countries. *Health Aff (Millwood)* 2016;**35**:1214–21. doi:10.1377/hlthaff.2015.0862

12 World Health Organization. *WHO package of essential noncommunicable (PEN) disease interventions for primary health care*. Geneva, Switzerland: : World Health Organization 2020. https://apps.who.int/iris/handle/10665/334186 (accessed 24 May 2022).

13 World Health Organization. Global action plan for the prevention and control of noncommunicable diseases 2013-2020. Geneva, Switzerland: : World Health Organization 2013.

14 Kabir A, Karim MN, Islam RM, *et al.* Health system readiness for non-communicable diseases at the primary care level: a systematic review. *BMJ Open* 2022;**12**:e060387. doi:10.1136/bmjopen-2021-060387

15 Rani M, Nusrat S, Hawken LH. A qualitative study of governance of evolving response to non-communicable diseases in low-and middle- income countries: current status, risks and options. *BMC Public Health* 2012;**12**:877. doi:10.1186/1471-2458-12-877

16 Nyaaba GN, Stronks K, de-Graft Aikins A, *et al.* Tracing Africa's progress towards implementing the Non-Communicable Diseases Global action plan 2013-2020: a synthesis of WHO country profile reports. *BMC Public Health* 2017;**17**:297. doi:10.1186/s12889-017-4199-6

17 Boudreaux C, Noble C, Coates MM, *et al.* Noncommunicable Disease (NCD) strategic plans in low- and lower-middle income Sub-Saharan Africa: framing and policy response. *Glob Health Action* 2020;**13**:1805165. doi:10.1080/16549716.2020.1805165

18 Jan S, Laba T-L, Essue BM, *et al.* Action to address the household economic burden of non-communicable diseases. *The Lancet* 2018;**391**:2047–58. doi:10.1016/S0140-6736(18)30323-4

19 Kankeu HT, Saksena P, Xu K, *et al.* The financial burden from non-communicable diseases in low- and middle-income countries: a literature review. *Health Res Policy Syst* 2013;**11**:31. doi:10.1186/1478-4505-11-31

20 World Health Organization. Everybody's business - strengthening health systems to improve health outcomes : WHO's framework for action. World Health Organization 2007. https://apps.who.int/iris/handle/10665/43918 (accessed 15 Jun 2022).

21 Andersen RM. Revisiting the Behavioral Model and Access to Medical Care: Does it Matter? *J Health Soc Behav* 1995;**36**:1. doi:10.2307/2137284

For peer review only - http://bmjopen.bmj.com/site/about/guidelines.xhtml

22 Manderson L, Smith-Morris C. Introduction: Chronicity and the Experience of Illness. In: *Introduction: Chronicity and the Experience of Illness*. Rutgers University Press 2010. 1–18. doi:10.36019/9780813549736-002

23 Levesque J-F, Harris MF, Russell G. Patient-centred access to health care: conceptualising access at the interface of health systems and populations. *Int J Equity Health* 2013;**12**:18. doi:10.1186/1475-9276-12-18

24 Brathwaite R, Hutchinson E, McKee M, *et al.* The Long and Winding Road: A Systematic Literature Review Conceptualising Pathways for Hypertension Care and Control in Low- and Middle-Income Countries. *Int J Health Policy Manag* 2020;:1. doi:10.34172/ijhpm.2020.105

25 World Health Organization. *Engaging the private health service delivery sector through governance in mixed health systems: strategy report of the WHO Advisory Group on the Governance of the Private Sector for Universal Health Coverage*. Geneva: : World Health Organization 2020. https://apps.who.int/iris/handle/10665/341057 (accessed 16 May 2022).

26 Lattof SR, Maliqi B. Private sector delivery of quality care for maternal, newborn and child health in low-income and middle-income countries: a mixed-methods systematic review protocol. *BMJ Open* 2020;**10**:e033141. doi:10.1136/bmjopen-2019-033141

27 Page MJ, McKenzie JE, Bossuyt PM, *et al.* The PRISMA 2020 statement: an updated guideline for reporting systematic reviews. *BMJ* 2021;:n71. doi:10.1136/bmj.n71

28 Rethlefsen ML, Kirtley S, Waffenschmidt S, *et al.* PRISMA-S: an extension to the PRISMA Statement for Reporting Literature Searches in Systematic Reviews. *Syst Rev* 2021;**10**:39. doi:10.1186/s13643-020-01542-z

29 Lim SS, Abbafati C, Abbas KM, *et al.* Global burden of 369 diseases and injuries in 204 countries and territories, 1990–2019: a systematic analysis for the Global Burden of Disease Study 2019. *The Lancet* 2020;**396**:1204–22. doi:10.1016/S0140-6736(20)30925-9

30 Klinton J. The Private Health Sector: An Operational Definition. Geneva, Switzerland: : World Health Organization 2020. https://www.who.int/docs/default-source/health-system-governance/private-health-sector-an-operational-definition.pdf?sfvrsn=5864e1f0_2&download=true (accessed 16 May 2022).

31 United Nations General Assembly. Political Declaration of the High-level Meeting of the General Assembly on the Prevention and Control of Non-communicable Diseases. New York, United States: : United Nations 2011. https://documents-dds-ny-un-

For peer review only - http://bmjopen.bmj.com/site/about/guidelines.xhtml

org.eur.idm.oclc.org/doc/UNDOC/LTD/N11/497/77/PDF/N1149777.pdf?OpenElement (accessed 8 Aug 2022).

32 Montagu Dominic, Andrew Anglemyer, Mudita Tiwari, *et al.* Private versus public strategies for health service provision for improving health outcomes in resource-limited settings. San Francisco, United States: : University of California 2011. https://eppi.ioe.ac.uk/cms/Default.aspx?tabid=3307

33 World Bank. World Bank Country and Lending Groups. 2022.https://datahelpdesk-worldbank-org.eur.idm.oclc.org/knowledgebase/articles/906519-world-bank-country-and-lending-groups

34 Bramer WM, Rethlefsen ML, Kleijnen J, *et al.* Optimal database combinations for literature searches in systematic reviews: a prospective exploratory study. *Syst Rev* 2017;**6**:245. doi:10.1186/s13643-017-0644-y

35 Harzing A-W. *Publish or Perish*. https://harzing.com/resources/publish-or-perish (accessed 23 Jun 2022).

36 Bramer WM, de Jonge GB, Rethlefsen ML, *et al.* A systematic approach to searching: an efficient and complete method to develop literature searches. *J Med Libr Assoc JMLA* 2018;**106**:531–41. doi:10.5195/jmla.2018.283

37 Bramer WM, Giustini D, de Jonge GB, *et al.* De-duplication of database search results for systematic reviews in EndNote. *J Med Libr Assoc JMLA* 2016;**104**:240–3. doi:10.3163/1536-5050.104.3.014

38 Van der Mierden S, Tsaioun K, Bleich A, *et al.* Software tools for literature screening in systematic reviews in biomedical research. *ALTEX* 2019;**36**:508–17. doi:10.14573/altex.1902131

39 Harrison H, Griffin SJ, Kuhn I, *et al.* Software tools to support title and abstract screening for systematic reviews in healthcare: an evaluation. *BMC Med Res Methodol* 2020;**20**:7. doi:10.1186/s12874-020-0897-3

40 Donabedian A. *The definition of quality and approaches to its assessment*. Ann Arbor, Mich: : Health Administration Press 1980.

41 Hong Q, Pluye P, Fàbregues S, *et al.* Mixed methods appraisal tool (MMAT), Version 2018. http://mixedmethodsappraisaltoolpublic.pbworks.com/w/file/fetch/127916259/MMAT_2018_criteria-manual_2018-08-01_ENG.pdf (accessed 9 Jun 2022).

For peer review only - http://bmjopen.bmj.com/site/about/guidelines.xhtml

Health seeking behaviours and private sector delivery of care for non-communicable diseases in low- and middle-income countries: protocol for a systematic review

Supplementary Material - Full Search Strategies

1. Embase

('non communicable disease'/mj/de OR neoplasm/mj/exp OR 'cancer patient'/mj/exp OR cardiology/mj/de OR 'cancer screening'/mj/de OR 'cancer surgery'/mj/de OR 'diabetes mellitus'/mj/exp OR 'cardiovascular disease'/mj/exp OR 'cerebrovascular disease'/mj/exp OR 'obstructive airway disease'/mj/exp OR 'chronic respiratory tract disease'/mj/de OR hypertension/mj/exp OR (((non-communicab* OR noncommunicab*) NEAR/3 disease*) OR cancer* OR ((heart OR cardiac OR cardiovascul* OR cerebrovascul* OR myocardial*) NEAR/3 (disease* OR patient* OR emergenc* OR attack* OR infarct*)) OR cardiolog* OR oncolog* OR diabet* OR (chronic NEAR/3 (respirator* OR lung* OR pulmonar*) NEAR/3 (failure OR disease*)) OR COPD OR asthma* OR bronchitis OR ((lung* OR pulmonar*) NEAR/3 (emphysem*)) OR ((cerebrovascul* OR cerebro-vascul*) NEAR/3 accident*) OR stroke OR hypertens* OR (surg* NEAR/3 emergenc*)):ti,kw) AND ('resource limited setting'/de OR 'low middle income country'/de OR 'low income country'/de OR 'middle income country'/de OR 'developing country'/de OR 'Africa'/exp OR 'Asia'/de OR 'South and Central America'/exp OR ((resource* NEAR/3 (limit* OR constrain* OR low)) OR ((low OR middle) NEAR/3 income* NEAR/3 countr*) OR LMIC* OR (developing NEAR/3 (countr* OR world* OR nation*)) OR third-world* OR 3rd-world* OR countries OR Afghan* OR albania* OR Algeria* OR American-somoa* OR Argentin* OR Armeni* OR asia OR AZERBAIJAN* OR Bangladesh* OR Belarus OR Belize OR Benin OR Bhutan* OR Bolivia* OR Bosnia* OR Botswan* OR Brazil* OR Bulgaria* OR Burkina-Faso OR Burundi OR Cabo-Verde* OR Central-African-Republic OR Chad OR China* OR Chinese* OR Colombia* OR Comoros OR Congo* OR Costa-Rica* OR Croatia* OR Cuba* OR Ecuador* OR Eritrea* OR Eswatini* OR Ethiopia* OR Fiji* OR Gabon* OR Gambia* OR Grenada* OR Guinea* OR Guatemal* OR Guyan* OR Haiti* OR Iran* OR Iraq* OR Jamaic* OR Jordan OR Kazakhst* OR DPR-Korea OR North-Korea OR Liberia* OR Latin-Americ* OR Madagasc* OR Malawi* OR Malaysia* OR Maldives* OR Mali OR malese OR Marshall-Island* OR Mexico* OR Mexican OR Micronesia* OR Mozambique* OR Nepal* OR Niger OR Palestin* OR Panama* OR Romania* OR Rwand* OR Samoa* OR Sao-Tome* OR Senegal* OR Sierra-Leon* OR Somali* OR South-Afric* OR Sudan* OR Syria* OR Tajiki* OR Tanzania* OR Togo* OR Tokolau* OR Tonga* OR Uganda* OR Yemen* OR Zimbabwe* OR Angol* OR Banglad* OR Bhutan* OR Bolivia* OR Cabo-Verd* OR Cambodia* OR Cameroon* OR Côte-d-Ivoir* OR Djibout* OR Dominica* OR Egypt* OR El-Salvador* OR (Georgia NOT (usa OR us OR united-states)) OR Ghan* OR Hondur* OR

For peer review only

India OR Indones* OR Kenya* OR Kiribati* OR Kosov* OR Kyrgyz* OR PDR-Lao OR Laotian* OR Lebanon* OR Lebanese* OR Lesoth* OR Libya* OR Mauritania* OR Mauritius* OR Micronesia* OR Moldova* OR Mongolia* OR Montenegr* OR Morocco* OR Myanmar* OR Namibia* OR Nauru* OR Nicaragua* OR Nigeria* OR Niue OR Macedoni* OR Pakistan* OR Papua* OR Paraguay* OR Peru OR Philippin* OR Russian-Feder* OR São-Tomé* OR Serbia* OR Solomon-Island* OR South-Afric* OR Sri-Lank* OR Saint-Lucia* OR Saint-Vincent* OR Sub-Sahara* OR Subsahara* OR Sudan* OR Surinam* OR Swaziland* OR Thailand* OR Timor-Leste OR Tunisia* OR Turkey OR Turkmenist* OR Tuvalu* OR Ukrain* OR Uzbeki* OR Vanuatu* OR Venezuel* OR Vietnam* OR West-Bank OR Gaza OR Zambia* OR africa):de,ab,ti,kw) AND ('health seeking behavior'/mj/de OR 'health care cost'/mj/exp OR 'health care quality'/mj/de OR 'affordability'/mj/de OR 'health care access'/mj/exp OR 'accessibility'/mj/de OR 'walking distance'/mj/exp OR 'health care delivery'/mj/de OR 'financial stress'/mj/exp OR 'reimbursement'/mj/de OR 'reputation'/de OR 'health care utilization'/mj/exp OR 'out of pocket cost'/de OR 'out of pocket expenditure'/de OR 'out of pocket payment'/de OR 'out of pocket spending'/de OR 'health inequalities'/de OR 'inequalities'/de OR 'cost effectiveness analysis'/mj/de OR 'financial deficit'/mj/exp OR 'patient empowerment'/mj/de OR ('empowerment'/mj/de AND 'patient'/mj/de) OR 'patient-reported outcome'/mj/de OR (((health) NEAR/3 (seeking* OR inequalit*)) OR ((health* OR hospital*) NEAR/3 (cost* OR expenditur* OR econom*)) OR ((health* OR care) NEAR/3 (qualit* OR evaluat* OR access* OR deliver* OR utilization* OR utilisation* OR "use")) OR affordab* OR access OR accessib* OR proximit* OR distance* OR ((financial* OR econom*) NEAR/3 (stress OR burden* OR distress* OR pressure* OR strain*)) OR ((opening* OR after OR out-of*) NEAR/3 (hour*)) OR in-network* OR reimbursement* OR reputation* OR out-of-pocket* OR ((indirect) NEAR/3 (cost* OR expendit*)) OR benefit-incidence* OR ((cost*) NEAR/3 (effectiv* OR efficien*)) OR debt* OR indebt* OR (empowerment* NEAR/3 (self OR patient*)) OR (patient-reported NEAR/3 (outcome* OR experience* OR satisfaction*))):ti,kw) AND ('health care facilities and services'/mj/exp OR 'health care personnel'/mj/exp OR 'private sector'/mj/exp OR 'hospitalization'/mj/exp OR 'pharmacy (shop)'/mj/exp OR 'clinical pharmacy'/mj/de OR (((health* OR care OR public* OR private* OR nonprofit* OR non-profit* OR faith*) NEAR/3 (facilit* OR provider* OR service* OR based OR sector)) OR hospital* OR clinic OR ((health*) NEAR/3 (center* OR centre* OR post)) OR ((family OR general) NEAR/3 (practitioner* OR doctor)) OR ((primary OR secondary OR tertiary OR nursing) NEAR/3 (care)) OR specialist OR specialists OR ((home) NEAR/3 (care OR nursing OR help OR service* OR treatment*)) OR homecare* OR cascade-of-care* OR pharmacy OR pharmacies):ti,kw) AND [2000-2030]/py NOT ('editorial'/de) NOT ([Conference Abstract]/lim OR [Conference Review]/lim) NOT ('case report'/de OR (case-report):ti)

For peer review only - http://bmjopen.bmj.com/site/about/guidelines.xhtml

2. Medline

(*Noncommunicable Diseases/ OR exp *Neoplasms/ OR exp *Cardiology/ OR exp *Early Detection of Cancer/ OR exp *Diabetes Mellitus/ OR exp *Cardiovascular Diseases/ OR exp *Cerebrovascular Disorders/ OR exp *"Pulmonary Disease, Chronic Obstructive"/ OR exp *Hypertension/ OR (((non-communicab* OR noncommunicab*) ADJ3 disease*) OR cancer* OR ((heart OR cardiac OR cardiovascul* OR cerebrovascul* OR myocardial*) ADJ3 (disease* OR patient* OR emergenc* OR attack* OR infarct*)) OR cardiolog* OR oncolog* OR diabet* OR (chronic ADJ3 (respirator* OR lung* OR pulmonar*) ADJ3 (failure OR disease*)) OR COPD OR asthma* OR bronchitis OR ((lung* OR pulmonar*) ADJ3 (emphysem*)) OR ((cerebrovascul* OR cerebro-vascul*) ADJ3 accident*) OR stroke OR hypertens* OR (surg* ADJ3 emergenc*)).ti,kf) AND (Developing Countries/ OR exp Africa/ OR Asia/ OR Latin America/ OR ((resource* ADJ3 (limit* OR constrain* OR low)) OR ((low OR middle) ADJ3 income* ADJ3 countr*) OR LMIC* OR (developing ADJ3 (countr* OR world* OR nation*)) OR third-world* OR 3rd-world* OR countries OR Afghan* OR alban* OR Algeria* OR American-somoa* OR Argentin* OR Armeni* OR asia OR AZERBAIJAN* OR Bangladesh* OR Belarus OR Belize OR Benin OR Bhutan* OR Bolivia* OR Bosnia* OR Botswan* OR Brazil* OR Bulgaria* OR Burkina-Faso OR Burundi OR Cabo-Verde* OR Central-African-Republic OR Chad OR China* OR Chinese* OR Colombia* OR Comoros OR Congo* OR Costa-Rica* OR Croatia* OR Cuba* OR Ecuador* OR Eritrea* OR Eswatini* OR Ethiopia* OR Fiji* OR Gabon* OR Gambia* OR Grenada* OR Guinea* OR Guatemal* OR Guyan* OR Haiti* OR Iran* OR Iraq* OR Jamaic* OR Jordan OR Kazakhst* OR DPR-Korea OR North-Korea OR Liberia* OR Latin-Americ* OR Madagasc* OR Malawi* OR Malaysia* OR Maldives* OR Mali OR malese OR Marshall-Island* OR Mexico* OR Mexican OR Micronesia* OR Mozambique* OR Nepal* OR Niger OR Palestin* OR Panama* OR Romania* OR Rwand* OR Samoa* OR Sao-Tome* OR Senegal* OR Sierra-Leon* OR Somali* OR South-Afric* OR Sudan* OR Syria* OR Tajiki* OR Tanzania* OR Togo* OR Tokolau* OR Tonga* OR Uganda* OR Yemen* OR Zimbabwe* OR Angol* OR Banglad* OR Bhutan* OR Bolivia* OR Cabo-Verd* OR Cambodia* OR Cameroon* OR Cote-d-Ivoir* OR Djibout* OR Dominica* OR Egypt* OR El-Salvador* OR (Georgia NOT (usa OR us OR united-states)) OR Ghan* OR Hondur* OR India OR Indones* OR Kenya* OR Kiribati* OR Kosov* OR Kyrgyz* OR PDR-Lao OR Laotian* OR Lebanon* OR Lebanese* OR Lesoth* OR Libya* OR Mauritania* OR Mauritius* OR Micronesia* OR Moldova* OR Mongolia* OR Montenegr* OR Morocco* OR Myanmar* OR Namibia* OR Nauru* OR Nicaragua* OR Nigeria* OR Niue OR Macedoni* OR Pakistan* OR Papua* OR Paraguay* OR Peru OR Philippin* OR Russian-Feder* OR Sao-Tome* OR Serbia* OR Solomon-Island* OR South-Afric* OR Sri-Lank* OR Saint-Lucia* OR Saint-Vincent* OR Sub-Sahara* OR Subsahara* OR Sudan* OR Surinam* OR Swaziland* OR Thailand* OR Timor-Leste OR Tunisia* OR Turkey OR Turkmenist* OR Tuvalu* OR Ukrain* OR Uzbeki* OR Vanuatu* OR Venezuel* OR Vietnam* OR West-Bank OR Gaza OR Zambia* OR africa).ab,ti,kf) AND (*"Patient Acceptance of Health Care"/ OR exp *Health Care Costs/OR *"Quality

For peer review only - http://bmjopen.bmj.com/site/about/guidelines.xhtml

of Health Care"/ OR *"Costs and Cost Analysis"/ OR exp *Health Services Accessibility/ OR *Delivery of Health Care/ OR *Financial Stress/ OR *"Insurance, Health, Reimbursement"/ OR *Health Expenditures/ OR *Healthcare Disparities/ OR *Cost-Benefit Analysis/ OR *Patient Participation/ OR (*Empowerment/ AND *Patients/) OR *Patient Reported Outcome Measures/ OR (((health) ADJ3 (seeking* OR inequalit*)) OR ((health* OR hospital*) ADJ3 (cost* OR expenditur* OR econom*)) OR ((health* OR care) ADJ3 (qualit* OR evaluat* OR access* OR deliver* OR utilization* OR utilisation* OR "use")) OR affordab* OR accessib* OR proximit* OR distance* OR ((financial* OR econom*) ADJ3 (stress OR burden* OR distress* OR pressure* OR strain*)) OR ((opening* OR after OR out-of*) ADJ3 (hour*)) OR in-network* OR reimbursement* OR reputation* OR out-of-pocket* OR ((indirect) ADJ3 (cost* OR expendit*)) OR benefit-incidence* OR ((cost*) ADJ3 (effectiv* OR efficien*)) OR debt* OR indebt* OR (empowerment* ADJ3 (self OR patient*)) OR (patient-reported ADJ3 (outcome* OR experience* OR satisfaction*))).ti,kf) AND (exp *"Health Care Facilities, Manpower, and Services"/ OR (((health* OR care OR public* OR private* OR nonprofit* OR non-profit* OR faith*) ADJ3 (facilit* OR provider* OR service* OR based OR sector)) OR hospital* OR clinic OR ((health*) ADJ3 (center* OR centre* OR post)) OR ((family OR general) ADJ3 (practitioner* OR doctor)) OR ((primary OR secondary OR tertiary OR nursing) ADJ3 (care)) OR specialist OR specialists OR ((home) ADJ3 (care OR nursing OR help OR service* OR treatment*)) OR homecare* OR cascade-of-care* OR pharmacy OR pharmacies).ti,kf) AND 2000:2030.(sa_year) NOT (Editorial/) NOT (news OR congres* OR abstract* OR book* OR chapter* OR dissertation abstract*).pt. NOT (Case Reports/ OR (case-report).ti.)

3. Web of Science

TI=(((((non-communicab* OR noncommunicab*) NEAR/2 disease*) OR cancer* OR ((heart OR cardiac OR cardiovascul* OR cerebrovascul* OR myocardial*) NEAR/2 (disease* OR patient* OR emergenc* OR attack* OR infarct*)) OR cardiolog* OR oncolog* OR diabet* OR (chronic NEAR/2 (respirator* OR lung* OR pulmonar*) NEAR/2 (failure OR disease*)) OR COPD OR asthma* OR bronchitis OR ((lung* OR pulmonar*) NEAR/2 (emphysem*)) OR ((cerebrovascul* OR cerebro-vascul*) NEAR/2 accident*) OR stroke OR hypertens* OR (surg* NEAR/2 emergenc*)))) AND TS=((((resource* NEAR/2 (limit* OR constrain* OR low)) OR ((low OR middle) NEAR/2 income* NEAR/2 countr*) OR LMIC* OR (developing NEAR/2 (countr* OR world* OR nation*)) OR third-world* OR 3rd-world* OR countries OR Afghan* OR albania* OR Algeria* OR American-somoa* OR Argentin* OR Armeni* OR asia OR AZERBAIJAN* OR Bangladesh* OR Belarus OR Belize OR Benin OR Bhutan* OR Bolivia* OR Bosnia* OR Botswan* OR Brazil* OR Bulgaria* OR Burkina-Faso OR Burundi OR Cabo-Verde* OR Central-African-Republic OR Chad OR China* OR Chinese* OR Colombia* OR Comoros OR Congo* OR Costa-Rica* OR Croatia* OR Cuba* OR Ecuador* OR Eritrea* OR Eswatini* OR Ethiopia* OR Fiji* OR Gabon* OR Gambia* OR

For peer review only - http://bmjopen.bmj.com/site/about/guidelines.xhtml

Grenada* OR Guinea* OR Guatemal* OR Guyan* OR Haiti* OR Iran* OR Iraq* OR Jamaic* OR Jordan OR Kazakhst* OR DPR-Korea OR North-Korea OR Liberia* OR Latin-Americ* OR Madagasc* OR Malawi* OR Malaysia* OR Maldives* OR Mali OR malese OR Marshall-Island* OR Mexico* OR Mexican OR Micronesia* OR Mozambique* OR Nepal* OR Niger OR Palestin* OR Panama* OR Romania* OR Rwand* OR Samoa* OR Sao-Tome* OR Senegal* OR Sierra-Leon* OR Somali* OR South-Afric* OR Sudan* OR Syria* OR Tajiki* OR Tanzania* OR Togo* OR Tokolau* OR Tonga* OR Uganda* OR Yemen* OR Zimbabwe* OR Angol* OR Banglad* OR Bhutan* OR Bolivia* OR Cabo-Verd* OR Cambodia* OR Cameroon* OR Cote-d-Ivoir* OR Djibout* OR Dominica* OR Egypt* OR El-Salvador* OR (Georgia NOT (usa OR us OR united-states)) OR Ghan* OR Hondur* OR India OR Indones* OR Kenya* OR Kiribati* OR Kosov* OR Kyrgyz* OR PDR-Lao OR Laotian* OR Lebanon* OR Lebanese* OR Lesoth* OR Libya* OR Mauritania* OR Mauritius* OR Micronesia* OR Moldova* OR Mongolia* OR Montenegr* OR Morocco* OR Myanmar* OR Namibia* OR Nauru* OR Nicaragua* OR Nigeria* OR Niue OR Macedoni* OR Pakistan* OR Papua* OR Paraguay* OR Peru OR Philippin* OR Russian-Feder* OR São-Tomé* OR Serbia* OR Solomon-Island* OR South-Afric* OR Sri-Lank* OR Saint-Lucia* OR Saint-Vincent* OR Sub-Sahara* OR Subsahara* OR Sudan* OR Surinam* OR Swaziland* OR Thailand* OR Timor-Leste OR Tunisia* OR Turkey OR Turkmenist* OR Tuvalu* OR Ukrain* OR Uzbeki* OR Vanuatu* OR Venezuel* OR Vietnam* OR West-Bank OR Gaza OR Zambia* OR africa))) AND TI=(((((health) NEAR/2 (seeking* OR inequalit*)) OR ((health* OR hospital*) NEAR/2 (cost* OR expenditur* OR econom*)) OR ((health* OR care) NEAR/2 (qualit* OR evaluat* OR access* OR deliver* OR utilization* OR utilisation* OR "use")) OR affordab* OR access OR accessib* OR proximit* OR distance* OR ((financial* OR econom*) NEAR/2 (stress OR burden* OR distress* OR pressure* OR strain*)) OR ((opening* OR after OR out-of*) NEAR/2 (hour*)) OR in-network* OR reimbursement* OR reputation* OR out-of-pocket* OR ((indirect) NEAR/2 (cost* OR expendit*)) OR benefit-incidence* OR ((cost*) NEAR/2 (effectiv* OR efficien*)) OR debt* OR indebt* OR (empowerment* NEAR/3 (self OR patient*)) OR (patient-reported NEAR/3 (outcome* OR experience* OR satisfaction*)))) AND ((((health* OR care OR public* OR private* OR nonprofit* OR non-profit* OR faith*) NEAR/2 (facilit* OR provider* OR service* OR based OR sector)) OR hospital* OR clinic OR ((health*) NEAR/2 (center* OR centre* OR post)) OR ((family OR general) NEAR/2 (practitioner* OR doctor)) OR ((primary OR secondary OR tertiary OR nursing) NEAR/2 (care)) OR specialist OR specialists OR ((home) NEAR/2 (care OR nursing OR help OR service* OR treatment*)) OR homecare* OR cascade-of-care* OR pharmacy OR pharmacies))) AND py=(2000-2030) AND DT=(Article OR Review OR Letter OR Early Access) NOT TI=(case-report)

4. Econlit

TI(non-communicab* OR noncommunicab* OR cancer* OR cardiac*OR cardiolog* OR oncolog* OR diabet* OR COPD OR asthma* OR bronchitis OR stroke OR hypertens*) **AND** ((((health* OR care OR

For peer review only - http://bmjopen.bmj.com/site/about/guidelines.xhtml

public* OR private* OR nonprofit* OR non-profit* OR faith*) N/2 (facilit* OR provider* OR service* OR based OR sector)) OR hospital* OR clinic OR ((health*) N/2 (center* OR centre* OR post)) OR ((family OR general) N/2 (practitioner* OR doctor)) OR ((primary OR secondary OR tertiary OR nursing) N/2 (care)) OR specialist OR specialists OR ((home) N/2 (care OR nursing OR help OR service* OR treatment*)) OR homecare* OR cascade-of-care* OR pharmacy OR pharmacies)) NOT PT (news OR comment* OR editorial* OR congres* OR abstract* OR book* OR chapter* OR dissertation abstract*) **AND** AB,TI((LMIC* OR third-world* OR 3rd-world* OR Afghan* OR albania* OR Algeria* OR American-somoa* OR Argentin* OR Armeni* OR asia OR AZERBAIJAN* OR Bangladesh* OR Belarus OR Belize OR Benin OR Bhutan* OR Bolivia* OR Bosnia* OR Botswan* OR Brazil* OR Bulgaria* OR Burkina-Faso OR Burundi OR Cabo-Verde* OR Central-African-Republic OR Chad OR China* OR Chinese* OR Colombia* OR Comoros OR Congo* OR Costa-Rica* OR Croatia* OR Cuba* OR Ecuador* OR Eritrea* OR Eswatini* OR Ethiopia* OR Fiji* OR Gabon* OR Gambia* OR Grenada* OR Guinea* OR Guatemal* OR Guyan* OR Haiti* OR Iran* OR Iraq* OR Jamaic* OR Jordan OR Kazakhst* OR DPR-Korea OR North-Korea OR Liberia* OR Latin-Americ* OR Madagasc* OR Malawi* OR Malaysia* OR Maldives* OR Mali OR malese OR Marshall-Island* OR Mexico* OR Mexican OR Micronesia* OR Mozambique* OR Nepal* OR Niger OR Palestin* OR Panama* OR Romania* OR Rwand* OR Samoa* OR Sao-Tome* OR Senegal* OR Sierra-Leon* OR Somali* OR South-Afric* OR Sudan* OR Syria* OR Tajiki* OR Tanzania* OR Togo* OR Tokolau* OR Tonga* OR Uganda* OR Yemen* OR Zimbabwe* OR Angol* OR Banglad* OR Bhutan* OR Bolivia* OR Cabo-Verd* OR Cambodia* OR Cameroon* OR Cote-d-Ivoir* OR Djibout* OR Dominica* OR Egypt* OR El-Salvador* OR (Georgia NOT (usa OR us OR united-states)) OR Ghan* OR Hondur* OR India OR Indones* OR Kenya* OR Kiribati* OR Kosov* OR Kyrgyz* OR PDR-Lao OR Laotian* OR Lebanon* OR Lebanese* OR Lesoth* OR Libya* OR Mauritania* OR Mauritius* OR Micronesia* OR Moldova* OR Mongolia* OR Montenegr* OR Morocco* OR Myanmar* OR Namibia* OR Nauru* OR Nicaragua* OR Nigeria* OR Niue OR Macedoni* OR Pakistan* OR Papua* OR Paraguay* OR Peru OR Philippin* OR Russian-Feder* OR São-Tomé* OR Serbia* OR Solomon-Island* OR South-Afric* OR Sri-Lank* OR Saint-Lucia* OR Saint-Vincent* OR Sub-Sahara* OR Subsahara* OR Sudan* OR Surinam* OR Swaziland* OR Thailand* OR Timor-Leste OR Tunisia* OR Turkey OR Turkmenist* OR Tuvalu* OR Ukrain* OR Uzbeki* OR Vanuatu* OR Venezuel* OR Vietnam* OR West-Bank OR Gaza OR Zambia* OR africa)) NOT PT (letter* OR news OR comment* OR editorial* OR congres* OR abstract* OR book* OR chapter* OR dissertation abstract*)

5. Global Index Medicus

TI:("non communicable" OR noncommunicable* OR cancer* OR cardiovascular* OR diabet* OR COPD OR asthma* OR bronchit* OR stroke OR hypertens* OR "heart disease" OR "cardiac disease" OR cerebrovascular* OR myocardial* OR "heart attack" OR "heart infarct" OR "cardiac attack" OR "cardiac

For peer review only - http://bmjopen.bmj.com/site/about/guidelines.xhtml

infarct" OR cardiolog* OR oncolog*) AND ti:("health seeking" OR "health inequality" OR "patient-acceptance" OR "health cost" OR "health evaluation" OR access OR affordabilit* OR "cost analysis" OR accessibility OR "financial stress" OR "patient reported" OR "walking distance" OR "out of pocket" OR "out-of-pocket" OR debt OR "patient empowerment" OR "self empowerment" OR "in network" OR "cost effectiveness" OR "cost efficiency" OR "patient reported outcome") AND TI:("health service" OR "health facility" OR "health provider" OR "health centre" OR "health center" OR "care service" OR "care facility" OR "care provider" OR "public service" OR "public facility" OR "public provider" OR "private service" OR "private facility" OR "private provider" OR hospital* OR clinic* OR specialist* OR homecare* OR non-profit* OR "family practitioner" OR "family doctor" OR "general practitioner")

6. Google Scholar

non-communicable|cancer|cardiovascular|diabetes|COPD|asthma|stroke|hypertension LMIC|'low middle income countries' 'health seeking|cost|quality|access|utilization' 'health provider|facility|center|centre|service'|hospital|clinic|pharmacy

For peer review only - http://bmjopen.bmj.com/site/about/guidelines.xhtml

PRISMA-P (Preferred Reporting Items for Systematic review and Meta-Analysis Protocols) 2015 checklist: recommended items to address in a systematic review protocol*

Section and topic	Item No	Checklist item	Page
**ADMINISTRATIVE INFORMATION**			
Title:			
Identification	1a	Identify the report as a protocol of a systematic review	1
Update	1b	If the protocol is for an update of a previous systematic review, identify as such	NA
Registration	2	If registered, provide the name of the registry (such as PROSPERO) and registration number	2
Authors:			
Contact	3a	Provide name, institutional affiliation, e-mail address of all protocol authors; provide physical mailing address of corresponding author	1
Contributions	3b	Describe contributions of protocol authors and identify the guarantor of the review	10
Amendments	4	If the protocol represents an amendment of a previously completed or published protocol, identify as such and list changes; otherwise, state plan for documenting important protocol amendments	NA
Support:			
Sources	5a	Indicate sources of financial or other support for the review	10
Sponsor	5b	Provide name for the review funder and/or sponsor	10
Role of sponsor or funder	5c	Describe roles of funder(s), sponsor(s), and/or institution(s), if any, in developing the protocol	10
**INTRODUCTION**			
Rationale	6	Describe the rationale for the review in the context of what is already known	1-2
Objectives	7	Provide an explicit statement of the question(s) the review will address with reference to participants, interventions, comparators, and outcomes (PICO)	4-5
**METHODS**			
Eligibility criteria	8	Specify the study characteristics (such as PICO, study design, setting, time frame) and report characteristics (such as years considered, language, publication status) to be used as criteria for eligibility for the review	4-5
Information sources	9	Describe all intended information sources (such as electronic databases, contact with study authors, trial registers or other grey literature sources) with planned dates of coverage	6
Search strategy	10	Present draft of search strategy to be used for at least one electronic database, including planned limits, such that it could be repeated	6-7
Study records:			7
Data management	11a	Describe the mechanism(s) that will be used to manage records and data throughout the review	

For peer review only

Selection process	11b	State the process that will be used for selecting studies (such as two independent reviewers) through each phase of the review (that is, screening, eligibility and inclusion in meta-analysis)	7
Data collection process	11c	Describe planned method of extracting data from reports (such as piloting forms, done independently, in duplicate), any processes for obtaining and confirming data from investigators	8
Data items	12	List and define all variables for which data will be sought (such as PICO items, funding sources), any pre-planned data assumptions and simplifications	8
Outcomes and prioritization	13	List and define all outcomes for which data will be sought, including prioritization of main and additional outcomes, with rationale	8
Risk of bias in individual studies	14	Describe anticipated methods for assessing risk of bias of individual studies, including whether this will be done at the outcome or study level, or both; state how this information will be used in data synthesis	8
Data synthesis	15a	Describe criteria under which study data will be quantitatively synthesised	8-9
	15b	If data are appropriate for quantitative synthesis, describe planned summary measures, methods of handling data and methods of combining data from studies, including any planned exploration of consistency (such as I^2, Kendall's τ)	NA
	15c	Describe any proposed additional analyses (such as sensitivity or subgroup analyses, meta-regression)	NA
	15d	If quantitative synthesis is not appropriate, describe the type of summary planned	8
Meta-bias(es)	16	Specify any planned assessment of meta-bias(es) (such as publication bias across studies, selective reporting within studies)	NA
Confidence in cumulative evidence	17	Describe how the strength of the body of evidence will be assessed (such as GRADE)	8

*** It is strongly recommended that this checklist be read in conjunction with the PRISMA-P Explanation and Elaboration (cite when available) for important clarification on the items. Amendments to a review protocol should be tracked and dated. The copyright for PRISMA-P (including checklist) is held by the PRISMA-P Group and is distributed under a Creative Commons Attribution Licence 4.0.**

From: Shamseer L, Moher D, Clarke M, Ghersi D, Liberati A, Petticrew M, Shekelle P, Stewart L, PRISMA-P Group. Preferred reporting items for systematic review and meta-analysis protocols (PRISMA-P) 2015: elaboration and explanation. BMJ. 2015 Jan 2;349(jan02 1):g7647.

For peer review only

BMJ Open

Health seeking behaviours and private sector delivery of care for non-communicable diseases in low- and middle-income countries: protocol for a systematic review

Journal:	*BMJ Open*
Manuscript ID	bmjopen-2022-066213.R2
Article Type:	Protocol
Date Submitted by the Author:	07-Jul-2023
Complete List of Authors:	Brindley, Callum; Erasmus University Rotterdam<br>Wijemunige, Nilmini; Erasmus University Rotterdam; Institute for Health Policy<br>Dieteren, Charlotte; Erasmus University Rotterdam<br>Bom, Judith; Erasmus University Rotterdam<br>Engel, Maarten; Erasmus University Rotterdam<br>Meessen, Bruno; World Health Organization<br>Bonfrer, IEJ ; Erasmus Universiteit Rotterdam,
**Primary Subject Heading**:	Global health
Secondary Subject Heading:	Health economics, Health services research
Keywords:	Health economics < HEALTH SERVICES ADMINISTRATION & MANAGEMENT, Health policy < HEALTH SERVICES ADMINISTRATION & MANAGEMENT, Organisation of health services < HEALTH SERVICES ADMINISTRATION & MANAGEMENT, Quality in health care < HEALTH SERVICES ADMINISTRATION & MANAGEMENT

SCHOLARONE™
Manuscripts

Health seeking behaviours and private sector delivery of care for non-communicable diseases in low- and middle-income countries: protocol for a systematic review

Authors list:

Callum Brindley[1,2 *], Nilmini Wijemunige[1,3], Charlotte Dieteren[1,2], Judith Bom[1,2], Maarten Engel[4], Bruno Meessen[5] and Igna Bonfrer[1,2]

Affiliations:

[1] Erasmus School of Health Policy and Management, Erasmus University Rotterdam, Rotterdam, The Netherlands

[2] Erasmus Centre for Health Economics, Erasmus University Rotterdam, Rotterdam, The Netherlands

[3] Institute for Health Policy, Colombo, Sri Lanka

[4] Medical Library, Erasmus Medical Centre, Erasmus University Rotterdam, Rotterdam, The Netherlands

[5] The World Health Organization, Geneva, Switzerland

*Correspondence author P.O. Box 1738, 3000 DR Rotterdam, The Netherlands. Email: brindley@eshpm.eur.nl

Tel: +31 10 408 8555

Word Count: 1915

For peer review only

For peer review only - http://bmjopen.bmj.com/site/about/guidelines.xhtml

Abstract

Introduction The burden of noncommunicable diseases (NCDs) has increased substantially in low- and middle-income countries (LMICs) and adapting health service delivery models to address this remains a challenge. Many NCD patients seek private care at different points in their encounters with the health system, but the determinants and outcomesof these choices are insufficiently understood. The proposed systematic review will help inform the governance of mixed health systems towards achieving the goal of universal health coverage. This protocol details our intended methodological and analytical approaches, based on the Preferred Reporting Items for Systematic Reviews and Meta-Analyses (PRISMA)

Methods and analysis Following the PRISMA approach, this systematic review will develop a descriptive synthesis of the determinants and outcomesof private health care utilisation for NCDs in LMICs. The databases Embase, Medline, Web of Science Core Collection, EconLit, Global Index Medicus and Google Scholar will be searched for relevant studies published in English between period 1 January 2010 and 30 June 2022 with additional searching of reference lists. The study selection process will involve a title-abstract and full text review, guided by clearly defined inclusion and exclusion criteria. A quality and risk of bias assessment will be done for each study using the Mixed Methods Appraisal Tool (MMAT).

Ethics and dissemination Ethical approval is not required because this review is based on data collected from publicly available materials. The results will be published in a peer-reviewed journal and presented at related scientific events.

PROSPERO registration number CRD42022340059

Keywords health seeking behaviour, private sector, noncommunicable diseases, cardiovascular disease, diabetes, respiratory diseases, cancer, low- and middle-income countries

Strengths and limitations of this study

- This is a systematic review protocol that proposes a timely contribution to the challenge of adapting health care service delivery to prevent, treat and manage NCDs in LMICs.
- We define the private sector broadly to capture its breadth and complexity.
- Although we will consider all LMICs, this systematic review is limited to studies published in English, so does not include relevant literature in other languages.
- We limit our attention to literature from the past decade which we contend is most relevant given recent changes in disease burden, risk factors and demographics in LMICs.
- We will not perform a meta-analysis because we expect highly diverse study characteristics, including design type, setting, intervention and outcome.

For peer review only

For peer review only - http://bmjopen.bmj.com/site/about/guidelines.xhtml

For peer review only

For peer review only - http://bmjopen.bmj.com/site/about/guidelines.xhtml

Introduction

The rising burden of non-communicable diseases (NCDs) in low- and middle-income countries (LMICs) [1,2] calls for policy responses that engage the whole health system, including private providers. While the appropriate role of the private sector in the delivery of health care has been heavily debated [3–6], it is well known that patients seek private care at different points in their encounters with the health system.[7–10] Research into the reasons for visiting private providers and the comparative quality of this care in LMICs has largely focused on infectious diseases, and maternal and child health.[11]

With regard to NCDs, the determinants and outcomes of private health care utilisation in LMICs remains insufficiently understood. Importantly, NCDs have multiple etiologies (including behavioural, environmental, and genetic) that require an organisation of services and delivery of care that is different from that of acute illnesses.[12] Specifically, a focus on prevention and primary care that empowers patients and prioritises information sharing and coordination across levels of care and providers.[13,12] This is imperative for the achievement of Sustainable Development Goal (SDG) target 3.4 – a one-third reduction in premature mortality from NCDs by 2030.

Adapting health service delivery models to respond to NCDs remains a challenge.[14] In many LMICs, national NCD strategies tend to focus on the public sector to the exclusion of the private sector despite the two being entwined elements of the whole health system.[15–17] Many LMICs with universal health coverage (UHC) benefits packages also only offer limited coverage for NCDs, such that patients must pay out-of-pocket not only in private health facilities but even in public ones.[18] The risk is that this discourages and distorts health seeking behaviours, leading to ineffective care, poor health outcomes and catastrophic health spending.[18,19] Understanding better the reality of health seeking behaviours for NCDs, including the possible role taken by private providers, will be key for the design and implementation of appropriate strategies and interventions, across the different building blocks of the health system.[20]

This study aims to synthesize the scattered evidence on how people with NCDs choose their healthcare providers in LMICs, and the outcomes of these choices, in the understudied private sector. This will provide important new insights into the determinants and outcomes of private health care utilisation for NCDs in LMICs. In particular, we concentrate on the contextual and individual factors that influence provider choice, patterns of utilisation, quality of care and financial protection. We use the notion of the patient journey and draw on existing conceptual frameworks of health seeking behaviour and the facilitators and barriers to accessing healthcare.[21–25] This systematic review has been initiated by

For peer review only - http://bmjopen.bmj.com/site/about/guidelines.xhtml

the World Health Organization's (WHO) Department of Noncommunicable Disease, and Department of Health System Financing and Governance. It follows work by a WHO Advisory Group on Governance of the Private Sector and a systematic review of private sector delivery of quality care for maternal, newborn and child health in LMICs.[26,27] This review will contribute to an understanding of health seeking behaviour for NCDs in the private sector in LMICs, which will in turn help inform the governance of mixed health systems in the pursuit of UHC and the achievement of the SDGs.

Methods and analysis

Aim and research questions

This systematic review on the determinants and outcomes of private health care utilisation is built around the questions – *What factors influence private health care seeking behaviour for individuals with NCDs in LMICs? From whom is health care obtained, what are the patterns of utilization, what are the determinants that influence the use of private as opposed to public health care, and what are the outcomes of this health care seeking?* This review allows us to determine the size and nature of the current literature and to identify major knowledge gaps that relate to mixed health care systems with entwined public and private providers of NCD care in LMICs.

Study design and protocol

This study is guided by the Preferred Reporting Items for Systematic Reviews and Meta-Analyses (PRISMA) and its extension for literature searches PRISMA-S.[28,29] PRISMA is a systematic approach to map and synthesise existing evidence, identify knowledge gaps and inform future research. This review was registered with PROSPERO (CRD42022340059) on 15 June 2022. We checked that there were no current or in-progress systematic reviews on the same topic by searching PROSPERO, the Research Registry, and the Open Science Framework.

Inclusion/exclusion criteria

Table 1 outlines the Populations, Interventions, Control, Outcomes, Timeframe, Setting (PICOTS) criteria to be used. We focus on adults aged 18 years or older and define the scope of health care to encompass the prevention, diagnosis, treatment and/or management of disease at all level (e.g.

For peer review only - http://bmjopen.bmj.com/site/about/guidelines.xhtml

primary health clinic to hospital). We restrict our search to four groups of NCDs responsible for over 80% of all premature NCD deaths and identified by SDG target 3.4. Specifically, cardiovascular diseases, cancers, respiratory diseases and diabetes. With regard to cancers, we limit our scope to the top five cancers with the greatest disease burden for each sex.[30] We use the World Health Organization's operational definition of the private health sector as the individuals and organizations that are neither owned nor directly controlled by governments, and are involved in the provision of health services (i.e. formal and informal providers as well as for-profit and non-profit entities.[31] Our outcomes of interest are the determinants and outcomesof health seeking behaviour at both the individual and collective level, which are described in more detailed under the search elements and key terms. We draw on existing theoretical approaches to health seeking behaviour and the facilitators and barriers to accessing healthcare.[21–25] **Error! Reference source not found.** depicts our conceptual framework of the non-linear interaction of the determinants and outcomes along the patient journey of seeking private health care for NCDs. We focus on the timeframe from January 2010 to June 2022, which is defined by large increases in the NCD disease burden in LMICs and the first High-Level Meeting of the UN General Assembly on the Prevention and Control of Non-communicable Diseases.[30,32] This also avoids duplication of a broader systematic review into the private provision of health services in LMICs conducted in 2011.[33] Lastly, the study looks at settings in LMICs as defined by the World Bank classification for 2022 (i.e. countries with a gross national income per capita of $4,095 or less).[34]

Table 1 - PICOTS criteria used in the systematic review

Population	Adults aged 18 years or older and households at risk, or diagnosed with at least one of the following non-communicable diseases: cardiovascular diseases, diabetes, chronic respiratory diseases or cancers (tracheal, bronchus and lung; colon and rectum; pancreas; stomach; breast and prostate)
Interventions	Private sector provision of health care services all levels (e.g. primary health clinic to hospital) that involves the prevention, diagnosis, treatment and/or management of non-communicable disease
Control	Not applicable
Outcomes	Determinants and outcomes of health care seeking behaviour, at the individual and population level
Timeframe	1 January 2010 to 30 June 2022
Setting	Low-income and middle-income countries following the World Bank 2022 classification [34]

For peer review only - http://bmjopen.bmj.com/site/about/guidelines.xhtml

For inclusion, studies must adhere to all elements defined in Table 1. They must be in English and published in a peer-reviewed journal. We will include both qualitative and quantitative studies, including randomized and non-randomized study designs (i.e., cross-sectional, case-control and cohort observational studies). Editorials, commentaries, reviews, and protocols will be excluded.

Studies must address private sector health care and may do so in a comparison with the public sector. However, we will exclude studies that only report aggregated data (i.e., private and public health sector outcome data combined). Lastly, we will also exclude vaccination programmes associated with NCDs (e.g., human papillomavirus) because they are government-led, as well as settings with irregular contextual circumstances (e.g., conflict, economic and political crises) since they are less likely to offer generalisable insights.

Search strategy

Our search strategy was developed with the assistance of an information specialist (ME). The following electronic databases will be searched: Embase, Medline, Web of Science, Econlit, Global Index Medicus.[35] We will also search Google Scholar and download the 200 most relevant references in line with common practice.[36] Table 2 provides the search elements and key terms we will use. Specifically, it contains terms for 1) non communicable diseases, 2) health care providers in LMICs, and 3) the determinants and outcomesof health seeking behaviour and related terms. The search was developed in Embase, optimized for sensitivity, then translated to other databases following the method described by Bramer et al 2018.[37] The search strategies for Embase and Medline use relevant thesaurus terms from Emtree and Medical Subject Headings (MeSH) respectively. In all databases, the terms will be searched in titles, author keywords and major index terms; the search element for setting (health care providers in LMICs) will also be searched in abstracts. The full search strategies of all databases are provided in the Supplementary Material. References will be imported into EndNote and duplicates removed.[38] Reference lists of revelvant reviews and all included articles will also be screened for potential additional sources missed by the search. We do not plan to contact authors or subject expects.

Table 2 - Search elements and key terms

Search element	Key terms

For peer review only - http://bmjopen.bmj.com/site/about/guidelines.xhtml

What: noncommunicable diseases	noncommunicable diseases; cardiovascular disease; heart attack; stroke; myocardial infarction; hypertension; diabetes; cancer (top 5 for each sex: lung, breast, colorectum, prostate, stomach, liver, cervix, thyroid); chronic respiratory disease; chronic obstructive pulmonary disease; asthma)
Where: health care providers in low- and middle-income countries	hospital, health clinic, community health centre, health centre, health post, family practitioner, general practitioner, primary care, secondary care, tertiary care, specialist, home care, nursing home, pharmacy, public provider, private provider, nonprofit provider, faith-based provider, low-income countries, middle-income countries, lower-middle income countries, upper-middle income countries, list of all LMIC countries
Why and what happens: determinants and outcomesof health care decisions / health seeking behaviour	quality; cost; affordability; accessibility; proximity; distance; opening hours; in-network provider; reimbursement; reputation; health service delivery; health outcomes; utilization; out-of-pocket payment; indirect costs; catastrophic health care expenditure; debt; health inequalities; health information; supply; fee for service; empowerment; patient reported experience

Screening process

The articles resulting from the search will be screened by a team of two pairs (CB-JB & NW-CD) working in the Rayyan Reference Manager.[39,40] After removing duplicates, each article will be title-abstract screened independently by two individuals using the eligibility criteria described above. To reduce bias, author names will not be visible during this screening stage. Before commencing screening by pairs, we will also conduct a pilot phase in which a random selection of 100 articles will be reviewed and discussed jointly to harmonise our approach. Articles adhering to all criteria will be selected for full-text review. Title-abstracts will be screened in three lots. Any conflicts will be reviewed within the pair at the conclusion of reviewing each lot, to allow for further harmonization within the pair. If an inclusion/exclusion decision cannot be made based on the title and abstract, the article will be advanced to full-text screening. In the case of a conflicting inclusion/exclusion decision, the other pair

For peer review only - http://bmjopen.bmj.com/site/about/guidelines.xhtml

in the team will also review the article and the supervising researcher (IB) will provide a final adjudication if needed. Articles selected for full-text screening will be reviewed by two individuals and a decision to exclude at this stage will be documented. Confirmed texts for inclusion in the systematic review will be advanced to the next stage of data extraction.

Data extraction

We will pilot and refine a data extraction framework including **study details** such as authors, title and year; and **study characteristics** such as research design, sample size, studied country, disease and provider type. We will extract the **determinants of selecting a health care provider**, grouped into three categories: 1) *individual factors* such as demographics and health; 2) *contextual factors* such availability and accessibility of health care; and 3) *perception of providers* such as quality and competence. Lastly, we extract **outcomes of selecting a health care provider**, which we will group into three categories: 1) *patterns of utilization* including characteristics of patients and equity issues; 2) *quality of care* including the Donabedian model[25] domains of process and outcomes as well as patient satisfaction and empowerment; and 3) *spending and financial protection* as captured by indicators such as catastrophic health spending and impoverishment. We will also consider socio-cultural, economic and political contextual factors, which are important determinants of health-seeking in LMICs. Each full-text article included in the systematic review will be read by two individuals with one person taking the lead to extract the data, and the second person reviewing this work.

In the sub-section 'Quality assessment', we explain how we will use the Mixed Methods Appraisal Tool to assess the methodological quality

Quality assessment

We will use the Mixed Methods Appraisal Tool (MMAT) to assess the methodological quality and risk of bias of the reviewed full-text articles.[41] MMAT is a critical appraisal tool intended for systematic reviews that include diverse study designs (i.e., quantitative, qualitative and mixed methods). Each full-text article will be assessed by a team of two pairs (CB-JB & NW-CD) with one person taking the lead and the second person reviewing for completeness and accuracy. Discrepancies and disagreements will be resolved through discussion.

Synthesis

For peer review only - http://bmjopen.bmj.com/site/about/guidelines.xhtml

The team will meet to discuss and analyse the data extracted, which we will consolidate using a descriptive synthesis including a summary of the evidence, gaps, and limitations. As described above in the data extraction, we will synthesise the results into three categories of determinants of selecting a health care provider (individual factors, contextual factors and perception of providers) and three categories of outcomes of selecting a health care provider(patterns of utilization, quality of care, and spending and financial protection). We will also develop a synthesis of the results that concern the interaction of health seeking determinants andoutcomes. A flow diagram of the inclusion/exclusion pathways and the descriptive statistics of the included studies and their outcomes will be developed to complement the analysis. We will not perform a meta-analysis because we expect highly diverse study characteristics, including design type, setting, intervention and outcome.

Patient and public involvement

None

Ethics and dissemination

Ethical approval is not required because this is a review and collection of data based on publicly available materials. The results will be presented at events attended by policy makers, academics and health care practitioners from LMICs, and later published in a topic relevant journal. Understanding the reality of health seeking behaviours for NCDs will be key for the design and implementation of interventions to strengthen the building blocks of the health system: from regulation of the health workforce and provider payment reform to benefit package design and the digitalisation of patient files. This systematic review will be a timely contribution to the challenge of adapting health care service delivery to prevent, treat and manage NCDs in LMICs.

For peer review only - http://bmjopen.bmj.com/site/about/guidelines.xhtml

Acknowledgements We wish to acknowledge the valuable contributions of Annalise Belloni, Owen O'Donnell, Tom van Ourti and Eddy van Doorslaer who provided input for this systematic review protocol.

Contributions IB, BM, ME and CB conceptualised the protocol. CB wrote the first draft with input from NW, CD, JB, ME and IB. All authors contributed to subsequent revisions and approved the protocol prior to its submission. All authors are accountable for the research presented.

Funding This work was supported jointly by the Non-Communicable Disease Department, and Health System Financing and Governance Department of the World Health Organization (Grant number 202758205). The content of this study is the sole responsibility of the authors and does not represent the official views of World Health Organization.

Competing interests None declared

Patient consent for publication Not applicable

Provenance and peer review Not commissioned, externally peer reviewed.

Data availability statement Data sharing not applicable as no datasets generated and/or analysed for this study

Supplementary material This content has been supplied by the author(s). It has not been vetted by BMJ Publishing Group Limited (BMJ) and may not have been peer-reviewed. Any opinions or recommendations discussed are solely those of the author(s) and are not endorsed by BMJ. BMJ disclaims all liability and responsibility arising from any reliance placed on the content. Where the content includes any translated material, BMJ does not warrant the accuracy and reliability of the translations (including but not limited to local regulations, clinical guidelines, terminology, drug names and drug dosages), and is not responsible for any error and/or omissions arising from translation and adaptation or otherwise.

Open access This is an open access article distributed in accordance with the Creative Commons Attribution Non Commercial (CC BY-NC 4.0) license, which permits others to distribute, remix, adapt, build upon this work non-commercially, and license their derivative works on different terms, provided the original work is properly cited, appropriate credit is given, any changes made indicated, and the use is non-commercial. See: http://creativecommons.org/licenses/by-nc/4.0/.

ORCID iDs

Callum Brindley https://orcid.org/0000-0002-5478-3974

Nilmini Wijemunige https://orcid.org/0000-0002-2241-3194

Charlotte Dieteren https://orcid.org/0000-0002-7809-1657

Judith Bom https://orcid.org/0000-0003-4477-7594

Maarten Engel https://orcid.org/0000-0002-1774-7042

Bruno Meessen https://orcid.org/0000-0002-0359-8621

Igna Bonfrer https://orcid.org/0000-0002-8570-0393

For peer review only - http://bmjopen.bmj.com/site/about/guidelines.xhtml

References

1 Bennett JE, Stevens GA, Mathers CD, *et al.* NCD Countdown 2030: worldwide trends in non-communicable disease mortality and progress towards Sustainable Development Goal target 3.4. *The Lancet* 2018;**392**:1072–88. doi:10.1016/S0140-6736(18)31992-5

2 Bukhman G. The Lancet NCDI Poverty Commission: bridging a gap in universal health coverage for the poorest billion. *The Lancet* 2020;**396**:991–1044. doi:10.1016/S0140-6736(20)31907-3

3 World Health Organization. *The World Health Report 2000. Health systems: improving performance*. Geneva, Switzerland: 2000.

4 World Bank. *World Development Report 2004: Making Services Work for Poor People*. Washington, DC, United States: : The World Bank 2003. doi:10.1596/0-8213-5468-X

5 Marriott A. Blind Optimism: Challenging the myths about private health care in poor countries. Oxfam International 2009. https://policy-practice.oxfam.org/resources/blind-optimism-challenging-the-myths-about-private-health-care-in-poor-countrie-114093/

6 Horton R, Clark S. The perils and possibilities of the private health sector. *The Lancet* 2016;**388**:540–1. doi:10.1016/S0140-6736(16)30774-7

7 Patouillard E, Goodman CA, Hanson KG, *et al.* Can working with the private for-profit sector improve utilization of quality health services by the poor? A systematic review of the literature. *Int J Equity Health* 2007;**6**:17. doi:10.1186/1475-9276-6-17

8 Berendes S, Heywood P, Oliver S, *et al.* Quality of private and public ambulatory health care in low and middle income countries: Systematic review of comparative studies. *PLoS Med* 2011;**8**:12. doi:10.1371/journal.pmed.1000433

9 Basu S, Andrews J, Kishore S, *et al.* Comparative Performance of Private and Public Healthcare Systems in Low- and Middle-Income Countries: A Systematic Review. *PLOS Med* 2012;**9**:e1001244. doi:10.1371/journal.pmed.1001244

10 Devi R, Kanitkar K, Narendhar R, *et al.* A Narrative Review of the Patient Journey Through the Lens of Non-communicable Diseases in Low- and Middle-Income Countries. *Adv Ther* 2020;**37**:4808–30. doi:10.1007/s12325-020-01519-3

For peer review only - http://bmjopen.bmj.com/site/about/guidelines.xhtml

11 Grépin KA. Private Sector An Important But Not Dominant Provider Of Key Health Services In Low- And Middle-Income Countries. *Health Aff (Millwood)* 2016;**35**:1214–21. doi:10.1377/hlthaff.2015.0862

12 World Health Organization. *WHO package of essential noncommunicable (PEN) disease interventions for primary health care*. Geneva, Switzerland: : World Health Organization 2020. https://apps.who.int/iris/handle/10665/334186 (accessed 24 May 2022).

13 World Health Organization. Global action plan for the prevention and control of noncommunicable diseases 2013-2020. Geneva, Switzerland: : World Health Organization 2013.

14 Kabir A, Karim MN, Islam RM, *et al.* Health system readiness for non-communicable diseases at the primary care level: a systematic review. *BMJ Open* 2022;**12**:e060387. doi:10.1136/bmjopen-2021-060387

15 Rani M, Nusrat S, Hawken LH. A qualitative study of governance of evolving response to non-communicable diseases in low-and middle- income countries: current status, risks and options. *BMC Public Health* 2012;**12**:877. doi:10.1186/1471-2458-12-877

16 Nyaaba GN, Stronks K, de-Graft Aikins A, *et al.* Tracing Africa's progress towards implementing the Non-Communicable Diseases Global action plan 2013-2020: a synthesis of WHO country profile reports. *BMC Public Health* 2017;**17**:297. doi:10.1186/s12889-017-4199-6

17 Boudreaux C, Noble C, Coates MM, *et al.* Noncommunicable Disease (NCD) strategic plans in low- and lower-middle income Sub-Saharan Africa: framing and policy response. *Glob Health Action* 2020;**13**:1805165. doi:10.1080/16549716.2020.1805165

18 Jan S, Laba T-L, Essue BM, *et al.* Action to address the household economic burden of non-communicable diseases. *The Lancet* 2018;**391**:2047–58. doi:10.1016/S0140-6736(18)30323-4

19 Kankeu HT, Saksena P, Xu K, *et al.* The financial burden from non-communicable diseases in low- and middle-income countries: a literature review. *Health Res Policy Syst* 2013;**11**:31. doi:10.1186/1478-4505-11-31

20 World Health Organization. Everybody's business - strengthening health systems to improve health outcomes : WHO's framework for action. World Health Organization 2007. https://apps.who.int/iris/handle/10665/43918 (accessed 15 Jun 2022).

21 Andersen RM. Revisiting the Behavioral Model and Access to Medical Care: Does it Matter? *J Health Soc Behav* 1995;**36**:1. doi:10.2307/2137284

For peer review only - http://bmjopen.bmj.com/site/about/guidelines.xhtml

22 Manderson L, Smith-Morris C. Introduction: Chronicity and the Experience of Illness. In: *Introduction: Chronicity and the Experience of Illness*. Rutgers University Press 2010. 1–18. doi:10.36019/9780813549736-002

23 Levesque J-F, Harris MF, Russell G. Patient-centred access to health care: conceptualising access at the interface of health systems and populations. *Int J Equity Health* 2013;**12**:18. doi:10.1186/1475-9276-12-18

24 Brathwaite R, Hutchinson E, McKee M, *et al.* The Long and Winding Road: A Systematic Literature Review Conceptualising Pathways for Hypertension Care and Control in Low- and Middle-Income Countries. *Int J Health Policy Manag* 2020;:1. doi:10.34172/ijhpm.2020.105

25 Donabedian A. *The definition of quality and approaches to its assessment*. Ann Arbor, Mich: : Health Administration Press 1980.

26 World Health Organization. *Engaging the private health service delivery sector through governance in mixed health systems: strategy report of the WHO Advisory Group on the Governance of the Private Sector for Universal Health Coverage*. Geneva: : World Health Organization 2020. https://apps.who.int/iris/handle/10665/341057 (accessed 16 May 2022).

27 Lattof SR, Maliqi B. Private sector delivery of quality care for maternal, newborn and child health in low-income and middle-income countries: a mixed-methods systematic review protocol. *BMJ Open* 2020;**10**:e033141. doi:10.1136/bmjopen-2019-033141

28 Page MJ, McKenzie JE, Bossuyt PM, *et al.* The PRISMA 2020 statement: an updated guideline for reporting systematic reviews. *BMJ* 2021;:n71. doi:10.1136/bmj.n71

29 Rethlefsen ML, Kirtley S, Waffenschmidt S, *et al.* PRISMA-S: an extension to the PRISMA Statement for Reporting Literature Searches in Systematic Reviews. *Syst Rev* 2021;**10**:39. doi:10.1186/s13643-020-01542-z

30 Lim SS, Abbafati C, Abbas KM, *et al.* Global burden of 369 diseases and injuries in 204 countries and territories, 1990–2019: a systematic analysis for the Global Burden of Disease Study 2019. *The Lancet* 2020;**396**:1204–22. doi:10.1016/S0140-6736(20)30925-9

31 Klinton J. The Private Health Sector: An Operational Definition. Geneva, Switzerland: : World Health Organization 2020. https://www.who.int/docs/default-source/health-system-governance/private-health-sector-an-operational-definition.pdf?sfvrsn=5864e1f0_2&download=true (accessed 16 May 2022).

For peer review only - http://bmjopen.bmj.com/site/about/guidelines.xhtml

32 United Nations General Assembly. Political Declaration of the High-level Meeting of the General Assembly on the Prevention and Control of Non-communicable Diseases. New York, United States: : United Nations 2011. https://documents-dds-ny-un-org.eur.idm.oclc.org/doc/UNDOC/LTD/N11/497/77/PDF/N1149777.pdf?OpenElement (accessed 8 Aug 2022).

33 Montagu Dominic, Andrew Anglemyer, Mudita Tiwari, *et al.* Private versus public strategies for health service provision for improving health outcomes in resource-limited settings. San Francisco, United States: : University of California 2011. https://eppi.ioe.ac.uk/cms/Default.aspx?tabid=3307

34 World Bank. World Bank Country and Lending Groups. 2022.https://datahelpdesk-worldbank-org.eur.idm.oclc.org/knowledgebase/articles/906519-world-bank-country-and-lending-groups

35 Bramer WM, Rethlefsen ML, Kleijnen J, *et al.* Optimal database combinations for literature searches in systematic reviews: a prospective exploratory study. *Syst Rev* 2017;**6**:245. doi:10.1186/s13643-017-0644-y

36 Harzing A-W. *Publish or Perish*. https://harzing.com/resources/publish-or-perish (accessed 23 Jun 2022).

37 Bramer WM, de Jonge GB, Rethlefsen ML, *et al.* A systematic approach to searching: an efficient and complete method to develop literature searches. *J Med Libr Assoc JMLA* 2018;**106**:531–41. doi:10.5195/jmla.2018.283

38 Bramer WM, Giustini D, de Jonge GB, *et al.* De-duplication of database search results for systematic reviews in EndNote. *J Med Libr Assoc JMLA* 2016;**104**:240–3. doi:10.3163/1536-5050.104.3.014

39 Van der Mierden S, Tsaioun K, Bleich A, *et al.* Software tools for literature screening in systematic reviews in biomedical research. *ALTEX* 2019;**36**:508–17. doi:10.14573/altex.1902131

40 Harrison H, Griffin SJ, Kuhn I, *et al.* Software tools to support title and abstract screening for systematic reviews in healthcare: an evaluation. *BMC Med Res Methodol* 2020;**20**:7. doi:10.1186/s12874-020-0897-3

41 Hong Q, Pluye P, Fàbregues S, *et al.* Mixed methods appraisal tool (MMAT), Version 2018. http://mixedmethodsappraisaltoolpublic.pbworks.com/w/file/fetch/127916259/MMAT_2018_criteria-manual_2018-08-01_ENG.pdf (accessed 9 Jun 2022).

For peer review only - http://bmjopen.bmj.com/site/about/guidelines.xhtml

Figure Legend:

Figure 1 – Conceptual framework

For peer review only

For peer review only - http://bmjopen.bmj.com/site/about/guidelines.xhtml

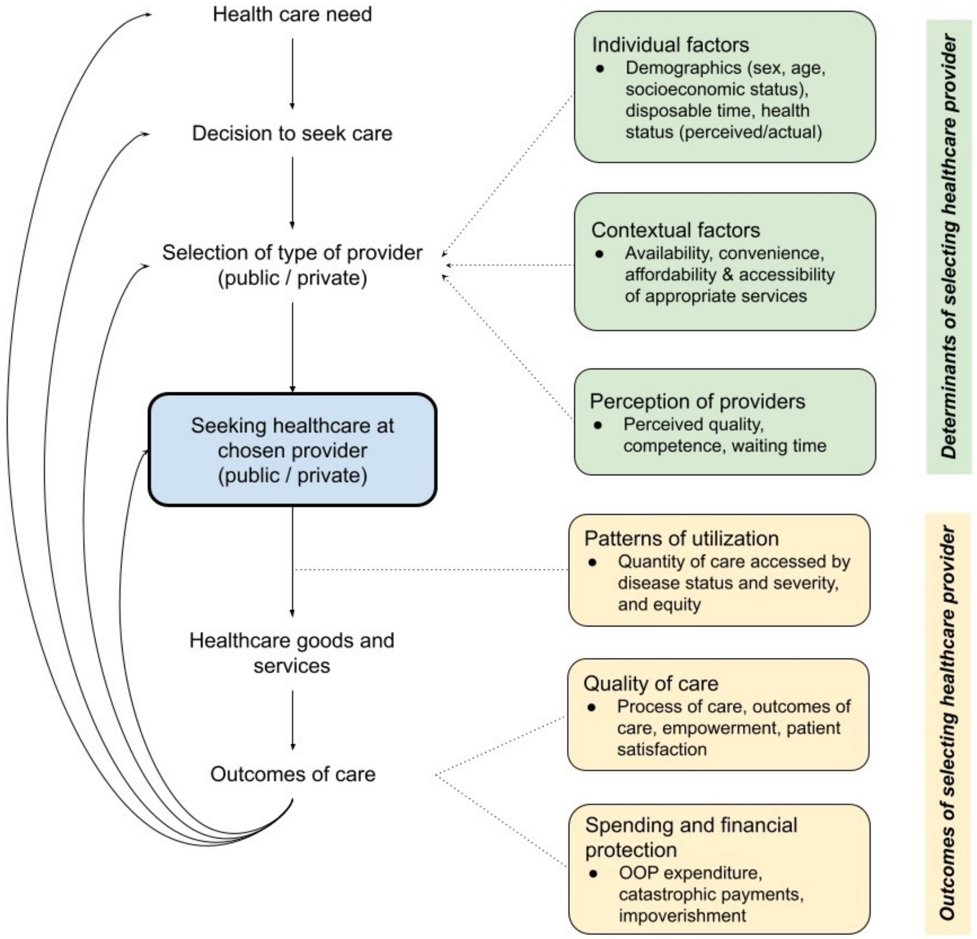

Conceptual framework

75x76mm (300 x 300 DPI)

Health seeking behaviours and private sector delivery of care for non-communicable diseases in low- and middle-income countries: protocol for a systematic review

Supplementary Material - Full Search Strategies

1. Embase

('non communicable disease'/mj/de OR neoplasm/mj/exp OR 'cancer patient'/mj/exp OR cardiology/mj/de OR 'cancer screening'/mj/de OR 'cancer surgery'/mj/de OR 'diabetes mellitus'/mj/exp OR 'cardiovascular disease'/mj/exp OR 'cerebrovascular disease'/mj/exp OR 'obstructive airway disease'/mj/exp OR 'chronic respiratory tract disease'/mj/de OR hypertension/mj/exp OR (((non-communicab* OR noncommunicab*) NEAR/3 disease*) OR cancer* OR ((heart OR cardiac OR cardiovascul* OR cerebrovascul* OR myocardial*) NEAR/3 (disease* OR patient* OR emergenc* OR attack* OR infarct*)) OR cardiolog* OR oncolog* OR diabet* OR (chronic NEAR/3 (respirator* OR lung* OR pulmonar*) NEAR/3 (failure OR disease*)) OR COPD OR asthma* OR bronchitis OR ((lung* OR pulmonar*) NEAR/3 (emphysem*)) OR ((cerebrovascul* OR cerebro-vascul*) NEAR/3 accident*) OR stroke OR hypertens* OR (surg* NEAR/3 emergenc*)):ti,kw) AND ('resource limited setting'/de OR 'low middle income country'/de OR 'low income country'/de OR 'middle income country'/de OR 'developing country'/de OR 'Africa'/exp OR 'Asia'/de OR 'South and Central America'/exp OR ((resource* NEAR/3 (limit* OR constrain* OR low)) OR ((low OR middle) NEAR/3 income* NEAR/3 countr*) OR LMIC* OR (developing NEAR/3 (countr* OR world* OR nation*)) OR third-world* OR 3rd-world* OR countries OR Afghan* OR albania* OR Algeria* OR American-somoa* OR Argentin* OR Armeni* OR asia OR AZERBAIJAN* OR Bangladesh* OR Belarus OR Belize OR Benin OR Bhutan* OR Bolivia* OR Bosnia* OR Botswan* OR Brazil* OR Bulgaria* OR Burkina-Faso OR Burundi OR Cabo-Verde* OR Central-African-Republic OR Chad OR China* OR Chinese* OR Colombia* OR Comoros OR Congo* OR Costa-Rica* OR Croatia* OR Cuba* OR Ecuador* OR Eritrea* OR Eswatini* OR Ethiopia* OR Fiji* OR Gabon* OR Gambia* OR Grenada* OR Guinea* OR Guatemal* OR Guyan* OR Haiti* OR Iran* OR Iraq* OR Jamaic* OR Jordan OR Kazakhst* OR DPR-Korea OR North-Korea OR Liberia* OR Latin-Americ* OR Madagasc* OR Malawi* OR Malaysia* OR Maldives* OR Mali OR malese OR Marshall-Island* OR Mexico* OR Mexican OR Micronesia* OR Mozambique* OR Nepal* OR Niger OR Palestin* OR Panama* OR Romania* OR Rwand* OR Samoa* OR Sao-Tome* OR Senegal* OR Sierra-Leon* OR Somali* OR South-Afric* OR Sudan* OR Syria* OR Tajiki* OR Tanzania* OR Togo* OR Tokolau* OR Tonga* OR Uganda* OR Yemen* OR Zimbabwe* OR Angol* OR Banglad* OR Bhutan* OR Bolivia* OR Cabo-Verd* OR Cambodia* OR Cameroon* OR Côte-d-Ivoir* OR Djibout* OR Dominica* OR Egypt* OR El-Salvador* OR (Georgia NOT (usa OR us OR united-states)) OR Ghan* OR Hondur* OR

For peer review only

India OR Indones* OR Kenya* OR Kiribati* OR Kosov* OR Kyrgyz* OR PDR-Lao OR Laotian* OR Lebanon* OR Lebanese* OR Lesoth* OR Libya* OR Mauritania* OR Mauritius* OR Micronesia* OR Moldova* OR Mongolia* OR Montenegr* OR Morocco* OR Myanmar* OR Namibia* OR Nauru* OR Nicaragua* OR Nigeria* OR Niue OR Macedoni* OR Pakistan* OR Papua* OR Paraguay* OR Peru OR Philippin* OR Russian-Feder* OR São-Tomé* OR Serbia* OR Solomon-Island* OR South-Afric* OR Sri-Lank* OR Saint-Lucia* OR Saint-Vincent* OR Sub-Sahara* OR Subsahara* OR Sudan* OR Surinam* OR Swaziland* OR Thailand* OR Timor-Leste OR Tunisia* OR Turkey OR Turkmenist* OR Tuvalu* OR Ukrain* OR Uzbeki* OR Vanuatu* OR Venezuel* OR Vietnam* OR West-Bank OR Gaza OR Zambia* OR africa):de,ab,ti,kw) AND ('health seeking behavior'/mj/de OR 'health care cost'/mj/exp OR 'health care quality'/mj/de OR 'affordability'/mj/de OR 'health care access'/mj/exp OR 'accessibility'/mj/de OR 'walking distance'/mj/exp OR 'health care delivery'/mj/de OR 'financial stress'/mj/exp OR 'reimbursement'/mj/de OR 'reputation'/de OR 'health care utilization'/mj/exp OR 'out of pocket cost'/de OR 'out of pocket expenditure'/de OR 'out of pocket payment'/de OR 'out of pocket spending'/de OR 'health inequalities'/de OR 'inequalities'/de OR 'cost effectiveness analysis'/mj/de OR 'financial deficit'/mj/exp OR 'patient empowerment'/mj/de OR ('empowerment'/mj/de AND 'patient'/mj/de) OR 'patient-reported outcome'/mj/de OR (((health) NEAR/3 (seeking* OR inequalit*)) OR ((health* OR hospital*) NEAR/3 (cost* OR expenditur* OR econom*)) OR ((health* OR care) NEAR/3 (qualit* OR evaluat* OR access* OR deliver* OR utilization* OR utilisation* OR "use")) OR affordab* OR access OR accessib* OR proximit* OR distance* OR ((financial* OR econom*) NEAR/3 (stress OR burden* OR distress* OR pressure* OR strain*)) OR ((opening* OR after OR out-of*) NEAR/3 (hour*)) OR in-network* OR reimbursement* OR reputation* OR out-of-pocket* OR ((indirect) NEAR/3 (cost* OR expendit*)) OR benefit-incidence* OR ((cost*) NEAR/3 (effectiv* OR efficien*)) OR debt* OR indebt* OR (empowerment* NEAR/3 (self OR patient*)) OR (patient-reported NEAR/3 (outcome* OR experience* OR satisfaction*))):ti,kw) AND ('health care facilities and services'/mj/exp OR 'health care personnel'/mj/exp OR 'private sector'/mj/exp OR 'hospitalization'/mj/exp OR 'pharmacy (shop)'/mj/exp OR 'clinical pharmacy'/mj/de OR (((health* OR care OR public* OR private* OR nonprofit* OR non-profit* OR faith*) NEAR/3 (facilit* OR provider* OR service* OR based OR sector)) OR hospital* OR clinic OR ((health*) NEAR/3 (center* OR centre* OR post)) OR ((family OR general) NEAR/3 (practitioner* OR doctor)) OR ((primary OR secondary OR tertiary OR nursing) NEAR/3 (care)) OR specialist OR specialists OR ((home) NEAR/3 (care OR nursing OR help OR service* OR treatment*)) OR homecare* OR cascade-of-care* OR pharmacy OR pharmacies):ti,kw) AND [2000-2030]/py NOT ('editorial'/de) NOT ([Conference Abstract]/lim OR [Conference Review]/lim) NOT ('case report'/de OR (case-report):ti)

For peer review only - http://bmjopen.bmj.com/site/about/guidelines.xhtml

2. Medline

(*Noncommunicable Diseases/ OR exp *Neoplasms/ OR exp *Cardiology/ OR exp *Early Detection of Cancer/ OR exp *Diabetes Mellitus/ OR exp *Cardiovascular Diseases/ OR exp *Cerebrovascular Disorders/ OR exp *"Pulmonary Disease, Chronic Obstructive"/ OR exp *Hypertension/ OR (((non-communicab* OR noncommunicab*) ADJ3 disease*) OR cancer* OR ((heart OR cardiac OR cardiovascul* OR cerebrovascul* OR myocardial*) ADJ3 (disease* OR patient* OR emergenc* OR attack* OR infarct*)) OR cardiolog* OR oncolog* OR diabet* OR (chronic ADJ3 (respirator* OR lung* OR pulmonar*) ADJ3 (failure OR disease*)) OR COPD OR asthma* OR bronchitis OR ((lung* OR pulmonar*) ADJ3 (emphysem*)) OR ((cerebrovascul* OR cerebro-vascul*) ADJ3 accident*) OR stroke OR hypertens* OR (surg* ADJ3 emergenc*)).ti,kf) AND (Developing Countries/ OR exp Africa/ OR Asia/ OR Latin America/ OR ((resource* ADJ3 (limit* OR constrain* OR low)) OR ((low OR middle) ADJ3 income* ADJ3 countr*) OR LMIC* OR (developing ADJ3 (countr* OR world* OR nation*)) OR third-world* OR 3rd-world* OR countries OR Afghan* OR alban* OR Algeria* OR American-somoa* OR Argentin* OR Armeni* OR asia OR AZERBAIJAN* OR Bangladesh* OR Belarus OR Belize OR Benin OR Bhutan* OR Bolivia* OR Bosnia* OR Botswan* OR Brazil* OR Bulgaria* OR Burkina-Faso OR Burundi OR Cabo-Verde* OR Central-African-Republic OR Chad OR China* OR Chinese* OR Colombia* OR Comoros OR Congo* OR Costa-Rica* OR Croatia* OR Cuba* OR Ecuador* OR Eritrea* OR Eswatini* OR Ethiopia* OR Fiji* OR Gabon* OR Gambia* OR Grenada* OR Guinea* OR Guatemal* OR Guyan* OR Haiti* OR Iran* OR Iraq* OR Jamaic* OR Jordan OR Kazakhst* OR DPR-Korea OR North-Korea OR Liberia* OR Latin-Americ* OR Madagasc* OR Malawi* OR Malaysia* OR Maldives* OR Mali OR malese OR Marshall-Island* OR Mexico* OR Mexican OR Micronesia* OR Mozambique* OR Nepal* OR Niger OR Palestin* OR Panama* OR Romania* OR Rwand* OR Samoa* OR Sao-Tome* OR Senegal* OR Sierra-Leon* OR Somali* OR South-Afric* OR Sudan* OR Syria* OR Tajiki* OR Tanzania* OR Togo* OR Tokolau* OR Tonga* OR Uganda* OR Yemen* OR Zimbabwe* OR Angol* OR Banglad* OR Bhutan* OR Bolivia* OR Cabo-Verd* OR Cambodia* OR Cameroon* OR Cote-d-Ivoir* OR Djibout* OR Dominica* OR Egypt* OR El-Salvador* OR (Georgia NOT (usa OR us OR united-states)) OR Ghan* OR Hondur* OR India OR Indones* OR Kenya* OR Kiribati* OR Kosov* OR Kyrgyz* OR PDR-Lao OR Laotian* OR Lebanon* OR Lebanese* OR Lesoth* OR Libya* OR Mauritania* OR Mauritius* OR Micronesia* OR Moldova* OR Mongolia* OR Montenegr* OR Morocco* OR Myanmar* OR Namibia* OR Nauru* OR Nicaragua* OR Nigeria* OR Niue OR Macedoni* OR Pakistan* OR Papua* OR Paraguay* OR Peru OR Philippin* OR Russian-Feder* OR Sao-Tome* OR Serbia* OR Solomon-Island* OR South-Afric* OR Sri-Lank* OR Saint-Lucia* OR Saint-Vincent* OR Sub-Sahara* OR Subsahara* OR Sudan* OR Surinam* OR Swaziland* OR Thailand* OR Timor-Leste OR Tunisia* OR Turkey OR Turkmenist* OR Tuvalu* OR Ukrain* OR Uzbeki* OR Vanuatu* OR Venezuel* OR Vietnam* OR West-Bank OR Gaza OR Zambia* OR africa).ab,ti,kf) AND (*"Patient Acceptance of Health Care"/ OR exp *Health Care Costs/OR *"Quality

For peer review only - http://bmjopen.bmj.com/site/about/guidelines.xhtml

of Health Care"/ OR *"Costs and Cost Analysis"/ OR exp *Health Services Accessibility/ OR *Delivery of Health Care/ OR *Financial Stress/ OR *"Insurance, Health, Reimbursement"/ OR *Health Expenditures/ OR *Healthcare Disparities/ OR *Cost-Benefit Analysis/ OR *Patient Participation/ OR (*Empowerment/ AND *Patients/) OR *Patient Reported Outcome Measures/ OR (((health) ADJ3 (seeking* OR inequalit*)) OR ((health* OR hospital*) ADJ3 (cost* OR expenditur* OR econom*)) OR ((health* OR care) ADJ3 (qualit* OR evaluat* OR access* OR deliver* OR utilization* OR utilisation* OR "use")) OR affordab* OR accessib* OR proximit* OR distance* OR ((financial* OR econom*) ADJ3 (stress OR burden* OR distress* OR pressure* OR strain*)) OR ((opening* OR after OR out-of*) ADJ3 (hour*)) OR in-network* OR reimbursement* OR reputation* OR out-of-pocket* OR ((indirect) ADJ3 (cost* OR expendit*)) OR benefit-incidence* OR ((cost*) ADJ3 (effectiv* OR efficien*)) OR debt* OR indebt* OR (empowerment* ADJ3 (self OR patient*)) OR (patient-reported ADJ3 (outcome* OR experience* OR satisfaction*))).ti,kf) AND (exp *"Health Care Facilities, Manpower, and Services"/ OR (((health* OR care OR public* OR private* OR nonprofit* OR non-profit* OR faith*) ADJ3 (facilit* OR provider* OR service* OR based OR sector)) OR hospital* OR clinic OR ((health*) ADJ3 (center* OR centre* OR post)) OR ((family OR general) ADJ3 (practitioner* OR doctor)) OR ((primary OR secondary OR tertiary OR nursing) ADJ3 (care)) OR specialist OR specialists OR ((home) ADJ3 (care OR nursing OR help OR service* OR treatment*)) OR homecare* OR cascade-of-care* OR pharmacy OR pharmacies).ti,kf) AND 2000:2030.(sa_year) NOT (Editorial/) NOT (news OR congres* OR abstract* OR book* OR chapter* OR dissertation abstract*).pt. NOT (Case Reports/ OR (case-report).ti.)

3. Web of Science

TI=(((((non-communicab* OR noncommunicab*) NEAR/2 disease*) OR cancer* OR ((heart OR cardiac OR cardiovascul* OR cerebrovascul* OR myocardial*) NEAR/2 (disease* OR patient* OR emergenc* OR attack* OR infarct*)) OR cardiolog* OR oncolog* OR diabet* OR (chronic NEAR/2 (respirator* OR lung* OR pulmonar*) NEAR/2 (failure OR disease*)) OR COPD OR asthma* OR bronchitis OR ((lung* OR pulmonar*) NEAR/2 (emphysem*)) OR ((cerebrovascul* OR cerebro-vascul*) NEAR/2 accident*) OR stroke OR hypertens* OR (surg* NEAR/2 emergenc*)))) AND TS=((((resource* NEAR/2 (limit* OR constrain* OR low)) OR ((low OR middle) NEAR/2 income* NEAR/2 countr*) OR LMIC* OR (developing NEAR/2 (countr* OR world* OR nation*)) OR third-world* OR 3rd-world* OR countries OR Afghan* OR albania* OR Algeria* OR American-somoa* OR Argentin* OR Armeni* OR asia OR AZERBAIJAN* OR Bangladesh* OR Belarus OR Belize OR Benin OR Bhutan* OR Bolivia* OR Bosnia* OR Botswan* OR Brazil* OR Bulgaria* OR Burkina-Faso OR Burundi OR Cabo-Verde* OR Central-African-Republic OR Chad OR China* OR Chinese* OR Colombia* OR Comoros OR Congo* OR Costa-Rica* OR Croatia* OR Cuba* OR Ecuador* OR Eritrea* OR Eswatini* OR Ethiopia* OR Fiji* OR Gabon* OR Gambia* OR

For peer review only - http://bmjopen.bmj.com/site/about/guidelines.xhtml

Grenada* OR Guinea* OR Guatemal* OR Guyan* OR Haiti* OR Iran* OR Iraq* OR Jamaic* OR Jordan OR Kazakhst* OR DPR-Korea OR North-Korea OR Liberia* OR Latin-Americ* OR Madagasc* OR Malawi* OR Malaysia* OR Maldives* OR Mali OR malese OR Marshall-Island* OR Mexico* OR Mexican OR Micronesia* OR Mozambique* OR Nepal* OR Niger OR Palestin* OR Panama* OR Romania* OR Rwand* OR Samoa* OR Sao-Tome* OR Senegal* OR Sierra-Leon* OR Somali* OR South-Afric* OR Sudan* OR Syria* OR Tajiki* OR Tanzania* OR Togo* OR Tokolau* OR Tonga* OR Uganda* OR Yemen* OR Zimbabwe* OR Angol* OR Banglad* OR Bhutan* OR Bolivia* OR Cabo-Verd* OR Cambodia* OR Cameroon* OR Cote-d-Ivoir* OR Djibout* OR Dominica* OR Egypt* OR El-Salvador* OR (Georgia NOT (usa OR us OR united-states)) OR Ghan* OR Hondur* OR India OR Indones* OR Kenya* OR Kiribati* OR Kosov* OR Kyrgyz* OR PDR-Lao OR Laotian* OR Lebanon* OR Lebanese* OR Lesoth* OR Libya* OR Mauritania* OR Mauritius* OR Micronesia* OR Moldova* OR Mongolia* OR Montenegr* OR Morocco* OR Myanmar* OR Namibia* OR Nauru* OR Nicaragua* OR Nigeria* OR Niue OR Macedoni* OR Pakistan* OR Papua* OR Paraguay* OR Peru OR Philippin* OR Russian-Feder* OR São-Tomé* OR Serbia* OR Solomon-Island* OR South-Afric* OR Sri-Lank* OR Saint-Lucia* OR Saint-Vincent* OR Sub-Sahara* OR Subsahara* OR Sudan* OR Surinam* OR Swaziland* OR Thailand* OR Timor-Leste OR Tunisia* OR Turkey OR Turkmenist* OR Tuvalu* OR Ukrain* OR Uzbeki* OR Vanuatu* OR Venezuel* OR Vietnam* OR West-Bank OR Gaza OR Zambia* OR africa))) AND TI=(((((health) NEAR/2 (seeking* OR inequalit*)) OR ((health* OR hospital*) NEAR/2 (cost* OR expenditur* OR econom*)) OR ((health* OR care) NEAR/2 (qualit* OR evaluat* OR access* OR deliver* OR utilization* OR utilisation* OR "use")) OR affordab* OR access OR accessib* OR proximit* OR distance* OR ((financial* OR econom*) NEAR/2 (stress OR burden* OR distress* OR pressure* OR strain*)) OR ((opening* OR after OR out-of*) NEAR/2 (hour*)) OR in-network* OR reimbursement* OR reputation* OR out-of-pocket* OR ((indirect) NEAR/2 (cost* OR expendit*)) OR benefit-incidence* OR ((cost*) NEAR/2 (effectiv* OR efficien*)) OR debt* OR indebt* OR (empowerment* NEAR/3 (self OR patient*)) OR (patient-reported NEAR/3 (outcome* OR experience* OR satisfaction*)))) AND ((((health* OR care OR public* OR private* OR nonprofit* OR non-profit* OR faith*) NEAR/2 (facilit* OR provider* OR service* OR based OR sector)) OR hospital* OR clinic OR ((health*) NEAR/2 (center* OR centre* OR post)) OR ((family OR general) NEAR/2 (practitioner* OR doctor)) OR ((primary OR secondary OR tertiary OR nursing) NEAR/2 (care)) OR specialist OR specialists OR ((home) NEAR/2 (care OR nursing OR help OR service* OR treatment*)) OR homecare* OR cascade-of-care* OR pharmacy OR pharmacies))) AND py=(2000-2030) AND DT=(Article OR Review OR Letter OR Early Access) NOT TI=(case-report)

4. Econlit

TI(non-communicab* OR noncommunicab* OR cancer* OR cardiac*OR cardiolog* OR oncolog* OR diabet* OR COPD OR asthma* OR bronchitis OR stroke OR hypertens*) **AND** ((((health* OR care OR

For peer review only - http://bmjopen.bmj.com/site/about/guidelines.xhtml

public* OR private* OR nonprofit* OR non-profit* OR faith*) N/2 (facilit* OR provider* OR service* OR based OR sector)) OR hospital* OR clinic OR ((health*) N/2 (center* OR centre* OR post)) OR ((family OR general) N/2 (practitioner* OR doctor)) OR ((primary OR secondary OR tertiary OR nursing) N/2 (care)) OR specialist OR specialists OR ((home) N/2 (care OR nursing OR help OR service* OR treatment*)) OR homecare* OR cascade-of-care* OR pharmacy OR pharmacies)) NOT PT (news OR comment* OR editorial* OR congres* OR abstract* OR book* OR chapter* OR dissertation abstract*) **AND** AB,TI((LMIC* OR third-world* OR 3rd-world* OR Afghan* OR albania* OR Algeria* OR American-somoa* OR Argentin* OR Armeni* OR asia OR AZERBAIJAN* OR Bangladesh* OR Belarus OR Belize OR Benin OR Bhutan* OR Bolivia* OR Bosnia* OR Botswan* OR Brazil* OR Bulgaria* OR Burkina-Faso OR Burundi OR Cabo-Verde* OR Central-African-Republic OR Chad OR China* OR Chinese* OR Colombia* OR Comoros OR Congo* OR Costa-Rica* OR Croatia* OR Cuba* OR Ecuador* OR Eritrea* OR Eswatini* OR Ethiopia* OR Fiji* OR Gabon* OR Gambia* OR Grenada* OR Guinea* OR Guatemal* OR Guyan* OR Haiti* OR Iran* OR Iraq* OR Jamaic* OR Jordan OR Kazakhst* OR DPR-Korea OR North-Korea OR Liberia* OR Latin-Americ* OR Madagasc* OR Malawi* OR Malaysia* OR Maldives* OR Mali OR malese OR Marshall-Island* OR Mexico* OR Mexican OR Micronesia* OR Mozambique* OR Nepal* OR Niger OR Palestin* OR Panama* OR Romania* OR Rwand* OR Samoa* OR Sao-Tome* OR Senegal* OR Sierra-Leon* OR Somali* OR South-Afric* OR Sudan* OR Syria* OR Tajiki* OR Tanzania* OR Togo* OR Tokolau* OR Tonga* OR Uganda* OR Yemen* OR Zimbabwe* OR Angol* OR Banglad* OR Bhutan* OR Bolivia* OR Cabo-Verd* OR Cambodia* OR Cameroon* OR Cote-d-Ivoir* OR Djibout* OR Dominica* OR Egypt* OR El-Salvador* OR (Georgia NOT (usa OR us OR united-states)) OR Ghan* OR Hondur* OR India OR Indones* OR Kenya* OR Kiribati* OR Kosov* OR Kyrgyz* OR PDR-Lao OR Laotian* OR Lebanon* OR Lebanese* OR Lesoth* OR Libya* OR Mauritania* OR Mauritius* OR Micronesia* OR Moldova* OR Mongolia* OR Montenegr* OR Morocco* OR Myanmar* OR Namibia* OR Nauru* OR Nicaragua* OR Nigeria* OR Niue OR Macedoni* OR Pakistan* OR Papua* OR Paraguay* OR Peru OR Philippin* OR Russian-Feder* OR São-Tomé* OR Serbia* OR Solomon-Island* OR South-Afric* OR Sri-Lank* OR Saint-Lucia* OR Saint-Vincent* OR Sub-Sahara* OR Subsahara* OR Sudan* OR Surinam* OR Swaziland* OR Thailand* OR Timor-Leste OR Tunisia* OR Turkey OR Turkmenist* OR Tuvalu* OR Ukrain* OR Uzbeki* OR Vanuatu* OR Venezuel* OR Vietnam* OR West-Bank OR Gaza OR Zambia* OR africa)) NOT PT (letter* OR news OR comment* OR editorial* OR congres* OR abstract* OR book* OR chapter* OR dissertation abstract*)

5. Global Index Medicus

TI:("non communicable" OR noncommunicable* OR cancer* OR cardiovascular* OR diabet* OR COPD OR asthma* OR bronchit* OR stroke OR hypertens* OR "heart disease" OR "cardiac disease" OR cerebrovascular* OR myocardial* OR "heart attack" OR "heart infarct" OR "cardiac attack" OR "cardiac

For peer review only - http://bmjopen.bmj.com/site/about/guidelines.xhtml

infarct" OR cardiolog* OR oncolog*) AND ti:("health seeking" OR "health inequality" OR "patient-acceptance" OR "health cost" OR "health evaluation" OR access OR affordabilit* OR "cost analysis" OR accessibility OR "financial stress" OR "patient reported" OR "walking distance" OR "out of pocket" OR "out-of-pocket" OR debt OR "patient empowerment" OR "self empowerment" OR "in network" OR "cost effectiveness" OR "cost efficiency" OR "patient reported outcome") AND TI:("health service" OR "health facility" OR "health provider" OR "health centre" OR "health center" OR "care service" OR "care facility" OR "care provider" OR "public service" OR "public facility" OR "public provider" OR "private service" OR "private facility" OR "private provider" OR hospital* OR clinic* OR specialist* OR homecare* OR non-profit* OR "family practitioner" OR "family doctor" OR "general practitioner")

6. Google Scholar

non-communicable|cancer|cardiovascular|diabetes|COPD|asthma|stroke|hypertension LMIC|'low middle income countries' 'health seeking|cost|quality|access|utilization' 'health provider|facility|center|centre|service'|hospital|clinic|pharmacy

For peer review only

PRISMA-P (Preferred Reporting Items for Systematic review and Meta-Analysis Protocols) 2015 checklist: recommended items to address in a systematic review protocol*

Section and topic	Item No	Checklist item	Page
**ADMINISTRATIVE INFORMATION**			
Title:			
Identification	1a	Identify the report as a protocol of a systematic review	1
Update	1b	If the protocol is for an update of a previous systematic review, identify as such	NA
Registration	2	If registered, provide the name of the registry (such as PROSPERO) and registration number	2
Authors:			
Contact	3a	Provide name, institutional affiliation, e-mail address of all protocol authors; provide physical mailing address of corresponding author	1
Contributions	3b	Describe contributions of protocol authors and identify the guarantor of the review	10
Amendments	4	If the protocol represents an amendment of a previously completed or published protocol, identify as such and list changes; otherwise, state plan for documenting important protocol amendments	NA
Support:			
Sources	5a	Indicate sources of financial or other support for the review	10
Sponsor	5b	Provide name for the review funder and/or sponsor	10
Role of sponsor or funder	5c	Describe roles of funder(s), sponsor(s), and/or institution(s), if any, in developing the protocol	10
**INTRODUCTION**			
Rationale	6	Describe the rationale for the review in the context of what is already known	1-2
Objectives	7	Provide an explicit statement of the question(s) the review will address with reference to participants, interventions, comparators, and outcomes (PICO)	4-5
**METHODS**			
Eligibility criteria	8	Specify the study characteristics (such as PICO, study design, setting, time frame) and report characteristics (such as years considered, language, publication status) to be used as criteria for eligibility for the review	4-5
Information sources	9	Describe all intended information sources (such as electronic databases, contact with study authors, trial registers or other grey literature sources) with planned dates of coverage	6
Search strategy	10	Present draft of search strategy to be used for at least one electronic database, including planned limits, such that it could be repeated	6-7
Study records:			7
Data management	11a	Describe the mechanism(s) that will be used to manage records and data throughout the review	

For peer review only

Selection process	11b	State the process that will be used for selecting studies (such as two independent reviewers) through each phase of the review (that is, screening, eligibility and inclusion in meta-analysis)	7
Data collection process	11c	Describe planned method of extracting data from reports (such as piloting forms, done independently, in duplicate), any processes for obtaining and confirming data from investigators	8
Data items	12	List and define all variables for which data will be sought (such as PICO items, funding sources), any pre-planned data assumptions and simplifications	8
Outcomes and prioritization	13	List and define all outcomes for which data will be sought, including prioritization of main and additional outcomes, with rationale	8
Risk of bias in individual studies	14	Describe anticipated methods for assessing risk of bias of individual studies, including whether this will be done at the outcome or study level, or both; state how this information will be used in data synthesis	8
Data synthesis	15a	Describe criteria under which study data will be quantitatively synthesised	8-9
	15b	If data are appropriate for quantitative synthesis, describe planned summary measures, methods of handling data and methods of combining data from studies, including any planned exploration of consistency (such as I^2, Kendall's τ)	NA
	15c	Describe any proposed additional analyses (such as sensitivity or subgroup analyses, meta-regression)	NA
	15d	If quantitative synthesis is not appropriate, describe the type of summary planned	8
Meta-bias(es)	16	Specify any planned assessment of meta-bias(es) (such as publication bias across studies, selective reporting within studies)	NA
Confidence in cumulative evidence	17	Describe how the strength of the body of evidence will be assessed (such as GRADE)	8

*** It is strongly recommended that this checklist be read in conjunction with the PRISMA-P Explanation and Elaboration (cite when available) for important clarification on the items. Amendments to a review protocol should be tracked and dated. The copyright for PRISMA-P (including checklist) is held by the PRISMA-P Group and is distributed under a Creative Commons Attribution Licence 4.0.**

From: Shamseer L, Moher D, Clarke M, Ghersi D, Liberati A, Petticrew M, Shekelle P, Stewart L, PRISMA-P Group. Preferred reporting items for systematic review and meta-analysis protocols (PRISMA-P) 2015: elaboration and explanation. BMJ. 2015 Jan 2;349(jan02 1):g7647.

For peer review only
